# Triple-Antiretroviral Prophylaxis to Prevent Mother-To-Child HIV Transmission through Breastfeeding—The Kisumu Breastfeeding Study, Kenya: A Clinical Trial

**DOI:** 10.1371/journal.pmed.1001015

**Published:** 2011-03-29

**Authors:** Timothy K. Thomas, Rose Masaba, Craig B. Borkowf, Richard Ndivo, Clement Zeh, Ambrose Misore, Juliana Otieno, Denise Jamieson, Michael C. Thigpen, Marc Bulterys, Laurence Slutsker, Kevin M. De Cock, Pauli N. Amornkul, Alan E. Greenberg, Mary Glenn Fowler, for the KiBS Study Team

**Affiliations:** 1 Division of HIV/AIDS Prevention, US Centers for Disease Control and Prevention (CDC), Atlanta, Georgia, United States of America; 2 Kenya Medical Research Institute (KEMRI)/CDC, Kisumu, Kenya; 3 Kenya Medical Research Institute, Kisumu, Kenya; 4 Provincial Medical Office - Nyanza Province, Ministry of Health, Kisumu, Kenya; 5 New Nyanza Provincial General Hospital, Kisumu, Kenya; 6 Division of Global HIV/AIDS, Center for Global Health, CDC, Atlanta, Georgia, United States of America; 7 Division of Parasitic Diseases, CDC, Atlanta, Georgia, United States of America; National Institute of Child Health and Human Development, United States of America

## Abstract

**Background:**

Effective strategies are needed for the prevention of mother-to-child HIV transmission (PMTCT) in resource-limited settings. The Kisumu Breastfeeding Study was a single-arm open label trial conducted between July 2003 and February 2009. The overall aim was to investigate whether a maternal triple-antiretroviral regimen that was designed to maximally suppress viral load in late pregnancy and the first 6 mo of lactation was a safe, well-tolerated, and effective PMTCT intervention.

**Methods and Findings:**

HIV-infected pregnant women took zidovudine, lamivudine, and either nevirapine or nelfinavir from 34–36 weeks' gestation to 6 mo post partum. Infants received single-dose nevirapine at birth. Women were advised to breastfeed exclusively and wean rapidly just before 6 mo. Using Kaplan-Meier methods we estimated HIV-transmission and death rates from delivery to 24 mo. We compared HIV-transmission rates among subgroups defined by maternal risk factors, including baseline CD4 cell count and viral load.

Among 487 live-born, singleton, or first-born infants, cumulative HIV-transmission rates at birth, 6 weeks, and 6, 12, and 24 mo were 2.5%, 4.2%, 5.0%, 5.7%, and 7.0%, respectively. The 24-mo HIV-transmission rates stratified by baseline maternal CD4 cell count <500 and ≥500 cells/mm^3^ were 8.4% (95% confidence interval [CI] 5.8%–12.0%) and 4.1% (1.8%–8.8%), respectively (*p* = 0.06); the corresponding rates stratified by baseline maternal viral load <10,000 and ≥10,000 copies/ml were 3.0% (1.1%–7.8%) and 8.7% (6.1%–12.3%), respectively (*p* = 0.01). None of the 12 maternal and 51 infant deaths (including two second-born infants) were attributed to antiretrovirals. The cumulative HIV-transmission or death rate at 24 mo was 15.7% (95% CI 12.7%–19.4%).

**Conclusions:**

This trial shows that a maternal triple-antiretroviral regimen from late pregnancy through 6 months of breastfeeding for PMTCT is safe and feasible in a resource-limited setting. These findings are consistent with those from other trials using maternal triple-antiretroviral regimens during breastfeeding in comparable settings.

**Trial registration:**

ClinicalTrials.gov NCT00146380

*Please see later in the article for the Editors' Summary*

## Abstract 2

Timothy Thomas and colleagues report the results of the Kisumu breastfeeding study (Kenya), a single-arm trial that assessed the feasibility and safety of a triple-antiretroviral regimen to suppress maternal HIV load in late pregnancy.

## Editors' Summary

**Background:**

Every year, about half a million children become infected with human immunodeficiency virus (HIV), which causes acquired immunodeficiency syndrome (AIDS). Nearly all these newly infected children live in resource-limited countries and most acquire HIV from their mother, so-called mother-to-child transmission (MTCT). Without intervention, 25%–50% of babies born to HIV-positive mothers become infected with HIV during pregnancy, delivery, or breastfeeding. This infection rate can be reduced by treating mother and child with antiretroviral (ARV) drugs. A single dose of nevirapine (a “non-nucleoside reverse transcriptase inhibitor” or NNRTI) given to the mother at the start of labor and to her baby soon after birth nearly halves the risk of MTCT. Further reductions in risk can be achieved by giving mother and baby three ARVs—an NNRTI and two nucleoside reverse transcriptase inhibitors (NRTIs such as zidovudine and lamivudine)—during pregnancy and perinatally (around the time of birth).

**Why Was This Study Done?:**

Breastfeeding is crucial for child survival in poor countries but it is also responsible for up to half of MTCT. Consequently, many researchers are investigating how various ARV regimens given to mothers and/or their infants during the first few months of life as well as during pregnancy and perinatally affect MTCT. In this single-arm trial, the researchers assess the feasibility and safety of using a triple-ARV regimen to suppress the maternal HIV load (amount of virus in the blood) from late pregnancy though 6 months of breastfeeding among HIV-positive women in Kisumu, Kenya, and ask whether this approach achieves a lower HIV transmission rate than other ARV regimens that have been tested in resource-limited settings. In a single-arm trial, all the participants are given the same treatment. By contrast, in a “randomized controlled” trial, half the participants chosen at random are given the treatment under investigation and the rest are given a control treatment. A randomized controlled trial provides a better comparison of treatments than a single-arm trial but is more costly.

**What Did the Researchers Do and Find?:**

In the Kisumu Breastfeeding Study (KiBS), HIV-infected pregnant women took a triple-ARV regimen containing zidovudine and lamivudine and either nevirapine or the protease inhibitor nelfinavir from 34–36 weeks of pregnancy to 6 months after delivery. They were advised to breastfeed their babies (who received single-dose nevirapine at birth), and to wean them rapidly just before 6 months. The researchers then used Kaplan-Meier statistical methods to estimate HIV transmission and death rates among 487 live-born infants from delivery to 24 months. The cumulative HIV transmission rate rose from 2.5% at birth to 7.0% at 24 months. The cumulative HIV transmission or death rate at 24 months was 15.7%; no infant deaths were attributed to ARVs. At 24 months, 3.0% of babies born to mothers with a low viral load were HIV positive compared to 8.7% of babies born to mothers with a high viral load, a statistically significant difference. Similarly, at 24 months, 8.4% of babies born to mothers with low baseline CD4 cell counts (CD4 cells are immune system cells that are killed by HIV; CD4 cell counts indicate the level of HIV-inflicted immune system damage) were HIV positive compared to 4.1% of babies born to mothers with high baseline CD4 cell counts, although this difference did not achieve statistical significance.

**What Do These Findings Mean?:**

Although these findings are limited by the single-arm design, they support the idea that giving breastfeeding women a triple-ARV regimen from late pregnancy to 6 months is a safe, feasible way to reduce MTCT in resource-limited settings. The HIV transmission rates in this study are comparable to those recorded in similar trials in other resource-limited settings and are lower than MTCT rates observed previously in Kisumu in a study in which no ARVs were used. Importantly, the KiBS mothers took most of the ARVs they were prescribed and most stopped breastfeeding by 6 months as advised. The intense follow-up employed in KiBS may be partly responsible for this good adherence to the trial protocol and thus this study's findings may not be generalizable to all resource-limited settings. Nevertheless, they suggest that a simple triple-ARV regimen given to HIV-positive pregnant women regardless of their baseline CD4 cell count can reduce MTCT during pregnancy and breastfeeding in resource-limited setting.

**Additional Information:**

Please access these Web sites via the online version of this summary at http://dx.doi.org/10.1371/journal.pmed.1001015.

- The accompanying *PLoS Medicine* Research article by Zeh and colleagues describes the emergence of resistance to ARVs in KiBS
- Information on HIV and AIDS is available from the US National Institute of Allergy and Infectious Diseases
- HIV InSite has comprehensive information on all aspects of HIV/AIDS
- Information is available from Avert, an international AIDS charity, on many aspects of HIV/AIDS, including information on children, HIV, and AIDS and on preventing mother-to-child transmission of HIV (in English and Spanish)
- UNICEF also has information about children and HIV and AIDS (in several languages)
- The World Health organization has information on mother-to-child transmission of HIV http://www.who.int/hiv/topics/mtct/en/index.html (in several languages)

## Introduction

UNAIDS and the World Health Organization (WHO) estimate that in 2009 there were 230,000 to 510,000 new HIV infections worldwide among children aged 0–15 y of age [1]. Over 95% of these infections occurred in resource-limited settings, primarily sub-Saharan Africa. Most infections resulted from mother-to-child transmission (MTCT). HIV MTCT rates in the absence of any antiretroviral (ARV) intervention among breastfeeding mothers who tested HIV antibody positive during pregnancy or delivery have ranged from 25% to 48% in various studies [2]. Breastfeeding, which is crucial to infant survival in resource-limited settings, accounts for one-third to one-half of MTCT [3]–[5]. Several trials of short-course/single-dose peripartum ARV regimens have reported 18-mo transmission rates between 6.8% and 15.9% [6]–[8]. However, in most of these studies, transmission events occurred while the infants were breastfeeding and not prophylaxed by maternal or infant ARVs [9].

For HIV-infected pregnant women in situations where replacement infant foods are not affordable, feasible, accessible, safe, and sustainable (AFASS conditions), 1998 WHO guidelines [10] encouraged exclusive breastfeeding for the first months of life followed by rapid weaning. Revised 2006 guidelines [11] recommended that AFASS conditions should be reassessed with the mother at 6 mo, and if not met, breastfeeding should continue with the introduction of complementary feeding.

In recognition of both the benefits and risks of breastfeeding for HIV-exposed infants, several trials have been conducted over the last decade to assess the impact on prevention of MTCT (PMTCT) of various combinations of ARV regimens given to mother and/or infant. We conducted the Kisumu Breastfeeding Study (KiBS) in Kisumu, Kenya; a PMTCT single-arm trial using a maternal triple-ARV regimen given to HIV-infected pregnant women beginning at 34 wk gestation and continuing for up to 6 mo post partum while exclusively breastfeeding. The primary objective of this trial was to assess whether the regimen was feasible, well tolerated, safe, and achieved a lower transmission rate compared to other short-course regimens evaluated among breastfeeding mothers in resource-limited settings.

## Methods

KiBS was a phase IIB open-label single-arm clinical/intervention trial approved by the ethical review committees of KEMRI (protocol 691) and US CDC (protocol 3677). See Protocol (Text S1) and CONSORT statement (Text S2).

### Primary Objectives

The primary objectives of this study using maternal triple-ARV for PMTCT in late pregnancy and during breast feeding were the following: (1) to detect a 50% reduction in the mother-to-child HIV-transmission rate at 18 mo compared to the corresponding rate using the single-dose nevirapine (NVP) regimen in the HIVNET 012 study [8]; (2) to detect a 50% improvement in the infant HIV-free survival rate at 18 mo compared to the corresponding rate using the single-dose NVP regimen in the HIVNET 012 study [8]; (3) to assess safety and toxicity for the mothers with use of the maternal triple-ARV prophylaxis particularly regarding rates of hepatic, hematologic, or dermatologic toxicities; (4) to assess adverse events or evidence of hepatic, hematologic, or dermatologic toxicity for infants exposed to low dose ARVs through maternal breast milk.

### Participants

Enrollment was conducted between July 2003 and November 2006; follow-up was completed in February 2009. Women were recruited through the PMTCT programs in the antenatal clinics of the New Nyanza Provincial General Hospital and the Kisumu District Hospital, both serving lower income populations of Kisumu and its environs. Pregnant women were invited to enroll if they were HIV positive and if, after receiving risk-benefit counseling on infant feeding options, they indicated intent to breastfeed. Enrollment criteria included: age ≥15 y, gestation of 34–36 wk, no previous ARV exposure, hemoglobin (Hb) ≥7 g/dl, absolute neutrophil count (ANC) >1,000 cells/mm^3^, platelets >50,000/ml, creatinine <1.5 mg/dl, and serum alanine aminotransferase (ALT) <2.5 times upper limit of normal.

### Procedures

Potential participants provided written informed consent, and at 32–34 wk gestation they underwent screening evaluations, which included a medical history, a physical examination, laboratory confirmation of HIV status, complete blood count, CD4 cell count, creatinine, and ALT tests. Participants started the triple-ARV regimen at 34–36 wk gestation and continued while breastfeeding, until 6 mo post partum. The initial regimen consisted of two nucleoside reverse transcriptase inhibitors and a non-nucleoside reverse transcriptase inhibitor. Between July 2003 and January 2005, regardless of CD4 cell count, mothers received zidovudine (ZDV) 300 mg and lamivudine (3TC) 150 mg in a fixed-dose combination pill (Combivir, GlaxoSmithKline), taken twice daily, and NVP 200 mg (Viramune, Boehringer Ingelheim), once daily for the first 2 wk and then twice daily thereafter. In January 2005, enrollment was halted because of reports of hepatotoxicity among women who started taking NVP when their CD4 count was ≥250 cells/mm^3^ [12]. Enrollment resumed in July 2005 with a revised regimen; women with a baseline CD4 count of ≥250 cells/mm^3^ received nelfinavir (NFV) (Viracept, Hoffman-La Roche Ltd) 1,250 mg (five 250 mg pills) twice daily instead of NVP. The decision to use NFV as an alternative ARV was based on the safety data available at that time and appropriateness of use in resource-limited settings. All women who met WHO treatment criteria (CD4 count of <200 cells/mm^3^ or WHO stage 3 or 4) [13] at enrollment or before 6 mo post partum remained on ARVs throughout the study; those who subsequently met WHO treatment criteria after stopping ARVs at 6 mo were restarted on treatment ARVs. Upon exiting the study, all participants were referred to facilities providing HIV care and treatment, including free ARVs. All women received trimethoprim-sulfamethoxazole (TMP/SMX) for prophylaxis of opportunistic infections throughout the study, except from 38 wk gestation until delivery because of concerns about neonatal hyperbilirubinemia.

Infants received single dose NVP (2 mg/kg) within 72 h of birth and TMP/SMX from 6 wk of age until they were no longer exposed to HIV through breastfeeding and were confirmed to be HIV negative. HIV-infected infants began ARV treatment when they met WHO infant treatment criteria [13].

Women were counseled to exclusively breastfeed for the first 5.5 mo and then to wean over 2 wk, with complete cessation of breastfeeding by 6 mo. We encouraged use of locally available foods (e.g., porridge, soups), and cow's milk for weaning and replacement feeding. We did not consider evaluating formula as a PMTCT intervention in accordance with guidance from the Provincial Ministry of Health about the risks of formula feeding, and in light of evidence of poor acceptance of free formula provided by UNICEF in Kenya [14].

Before delivery, participants were evaluated weekly. After delivery, each mother and her infant(s) were followed for 24 mo: study visits were scheduled at delivery (0–7 d), 2, 6, 10, and 14 wk post partum, and 6, 9, 12, 15, 18, and 24 mo post partum. Three visits were added at 5, 7, and 8 mo to monitor infant weight around weaning. During study visits, we assessed adherence to drugs and infant feeding recommendations, performed clinical evaluations, and obtained blood for hematologic, biochemical, and virologic monitoring. Adherence to study ARVs was assessed through pill counts, calculated as the percentage of pills dispensed but not returned out of the total number of pills dispensed. The study provided or covered the cost for all outpatient and inpatient care required by participants while on study.

All laboratory testing was done at the KEMRI/CDC laboratory in Kisumu. Hematologic testing was done using a Coulter A^c^.T 5diff CP analyzer (Beckman Coulter). Lymphocyte subsets were analyzed on a FACSCalibur flow cytometer (Becton Dickinson). Biochemistry analysis was done using the Cobas Integra 400 plus biochemistry analyzer (Roche). Plasma viral loads were quantified using the Amplicor HIV-1 RNA Monitor Test v1.5 (Roche Diagnostics). HIV DNA testing by PCR was done using Amplicor HIV-1 DNA PCR assay v1.5 (Roche Diagnostics). ELISA was done using Vironostika HIV Uniform II plus O kit (Organon Teknika) and Enzygnost (Dade Behring Marburg GmbH).

For HIV testing of infants we used dried blood spots collected at birth (0–7 d), at 2, 6, and 14 wk, and at 6, 9, 12, 18, and 24 mo. DNA PCR testing was performed in real time on the 14-wk and 6- and 9-mo specimens. ELISA was performed at 18 and 24 mo, with confirmation of positive results by PCR. After any positive test result, PCR testing was performed sequentially on prior untested specimens in order to identify the first positive specimen.

We graded adverse events according to the 1992 Adult and 1994 Pediatric toxicity tables [15],[16] of the Division of AIDS (DAIDS), US National Institutes of Health (NIH). Serious adverse events (SAEs) were defined as grade 3 or 4 toxicity confirmed on a repeat visit or as illness resulting in death or hospitalization. In addition, grade 2 hepatotoxicity events and grade 2 rashes with signs of hypersensitivity reaction were classified as SAEs. SAEs that occurred while participants were taking ARVs or within 3 mo of their cessation were considered possibly related to an ARV if an increased risk was noted in the prescribing information for the drug [17],[18] or if no alternative cause could be found. We did not rechallenge participants with the potentially causative ARV. We confine this analysis to those SAEs occurring between enrollment and 9 mo post partum. Abnormal laboratory findings were confirmed prior to any change in ARV regimen. Causes of death were determined by review of hospital records (if available) and/or by interviewing the mother, spouse, or caretaker (verbal autopsy).

### Statistical Analysis

We performed a series of sample size and power calculations under a variety of assumptions. Historically, the cumulative HIV-transmission rate in the NVP arm of the HIVNET 012 study [8] was 11.8% at 6 wk and 15.7% at 18 mo of age. We wanted to be able to detect a 50% reduction in the corresponding rate in KiBS using a one-sided exact binomial test at the 0.025 significance level, as an approximation to the test based on the Kaplan-Meier function for low rates. For 95% statistical power, if the true HIV-transmission rate in KiBS was 8%, 9%, or 10%, then 225, 305, or 425 children, respectively, would be needed for analysis at 18 mo of age. We further inflated these sample sizes by a factor of 1/(1−0.2) to account for maternal and child drop-out, as well as other sources of heterogeneity, thus giving a required maternal enrolment of 282, 382, or 532, respectively. Standard summary statistics were used to describe sociodemographic, clinical, laboratory, delivery, and adherence data. All statistical calculations were performed using SAS statistical software (SAS Institute).

For the evaluation of HIV transmission we included only live-born singleton or first-born infants who had any HIV test data. For our purposes, “first-born” means the first infant born in a multiple birth (e.g., the first twin). For infants testing HIV positive, the time of HIV transmission was estimated as the midpoint between the last negative and first positive test result. All infants with PCR-positive samples before 7 d of age were assumed to have been born infected. We used the Kaplan-Meier method to estimate HIV-transmission rates, death rates, and combined HIV-transmission or death rates over the 2-y follow-up period, and constructed 95% confidence interval (CIs) using the log-log transformation. We included all infants regardless of maternal adherence to intervention or maternal death. Serial pregnancies were not included.

For the HIV-transmission analysis, the endpoint was the estimated time of HIV transmission or was censored at the last negative test result. For the death analysis, the endpoint was the time of death or was censored at the last time observed alive. For the combined HIV transmission or death analysis, the endpoint was estimated HIV-transmission time, or death time (if not HIV infected), or was censored at the last negative test result.

In addition, we stratified the HIV-transmission rates by selected variables of interest, including study period (i.e., before or after the introduction of NFV in July 2005), infant gender, maternal baseline CD4 count and viral load, and maternal regimen for those with maternal baseline CD4 counts ≥250 cells/mm^3^. We performed log-rank tests to compare the survival curves obtained in these stratified analyses over the 2-y follow-up period. We also used normal approximation methods to compare differences between the Kaplan-Meier estimates of the stratified HIV-transmission rates at 24 mo. Finally, we calculated relative risks (RRs) with 95% CIs to determine whether hepatotoxicity and rash SAEs were associated with baseline maternal CD4 counts (<250 and ≥250 cells/mm^3^) among the 310 women whose initial triple-ARV regimen included NVP.

## Results

**Figure 1:**
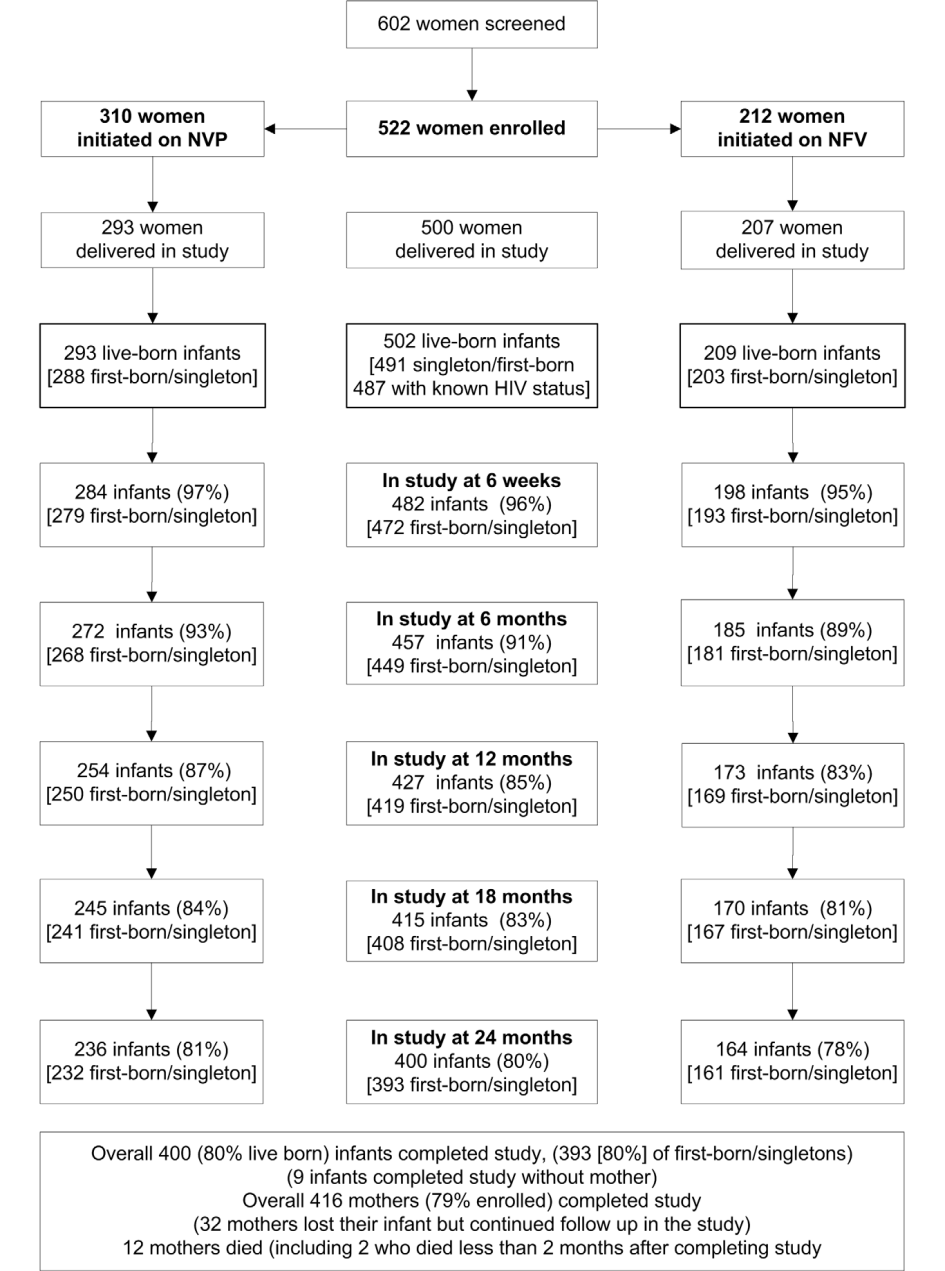
Recruitment, enrollment, and follow-up of participating women and infants. KiBS, 2003–2009.

Between July 2003 and November 2006, we screened 602 HIV-positive women, recruited from antenatal PMTCT clinics; 522 (87%) met eligibility criteria and were enrolled (Figure 1). The main reasons for ineligibility included being over 36 wk gestation (40%), Hb<7 gm/dl (12%), unable or unwilling to comply with study procedures (18%), HIV negative on confirmatory testing (9%), delivered before enrollment (8%), other abnormal laboratory test (5%), and other reasons (8%). The median age of those enrolled was 23 y (range 15–43 y) (Table 1). The median CD4 count was 398 cells/mm^3^; 74 (14%) women had a CD4 count of <200 cells/mm^3^. NVP- or NFV-based triple-ARV prophylaxis was initiated in 310 women and 212 women respectively.

**Table 1:** Maternal baseline sociodemographic, clinical, and laboratory characteristics.

Variable (*n* = 522)	Category	Number (%) or Median (Range)
Ethnic group	Luo	447 (86%)
	Luhya	54 (10%)
	Other	21 (4%)
Median age (y) (*n* = 520)		23 (15–43)
Primigravid	Yes	130 (25%)
	No	392 (75%)
Median parity, among multigravid (*n* = 392)		2 (0–8)
Marital status	Single	69 (13%)
	Married	388 (74%)
	Separated	26 (5%)
	Widowed	39 (8%)
Living with child's father (*n* = 521)	Yes	376 (72%)
	No	145 (28%)
Completed primary education (8 y)	Yes	368 (70%)
	No	154 (30%)
Employed outside home	Yes	174 (33%)
	No	348 (67%)
Median *n* people in household (*n* = 521)		3 (1–14)
Access to water (*n* = 520)	Piped to house	26 (5%)
	Public tap	401 (77%)
	Well, borehole	59 (11%)
	River, lake, rain	34 (7%)
Toilet facility	Private/shared flush toilet	60 (11%)
	Pit, latrine, bush	462 (89%)
Hb (g/dl) (*n* = 520)		9.8 (7.0–13.9)
Median viral load (copies/ml) (*n* = 520)		33,975 (0–2.9 million)
Viral load (copies/ml) (*n* = 520)	Undetectable (<400)	27 (5%)
	400–9,999	120 (23%)
	10,000–49,999	151 (29%)
	≥50,000	222 (43%)
Median CD4 count (cells/mm^3^) (*n* = 520)		398 (32–1,340)
CD4 count (cells/mm^3^) (*n* = 520)	<200	74 (14%)
	200–349	130 (25%)
	350–499	144 (28%)
	≥500	172 (33%)
WHO clinical HIV disease stage	Stage 1	439 (84%)
	Stage 2	45 (9%)
	Stage 3 or 4	38 (7%)
Initial triple-ARV regimen	ZDV/3TC plus NVP	310 (59%)
	ZDV/3TC plus NFV	212 (41%)

Percentages were computed excluding individuals with missing data for that particular characteristic. *n* = 522 unless stated.

A total of 511 infants were delivered, including nine sets of twins and one set of triplets (Table 2). There were nine stillbirths (all singletons), resulting in 502 live births (275 [57%] male, 227 [43%] female). No specimens were obtained from five infants (all males) who died within 1 wk of delivery. Overall, 457 (91%), 427 (85%), and 400 (80%) live-born infants remained in the study at 6, 12, and 24 mo, respectively (Figure 1). There were 491 live-born singletons and first borns.

**Table 2:** Maternal and infant delivery characteristics.

Variable (*n* = 500)	Category	*n* (%) or Median (Range)
Location of delivery	Hospital^a^	392 (78%)
	Other health facility	22 (4%)
	Participant's home	45 (9%)
	TBA home	30 (6%)
	Other/unknown	11 (2%)
Median gestation (wk)		39 (33–48)
Gestation (wk)	<37	72 (14%)
	37–42	355 (71%)
	>42	73 (15%)
Median no. days on ARVs at delivery (*n* = 498)		39 (1–100)
Days on ARVs at delivery (*n* = 498)	<28 d	130 (26%)
	≥28	368 (74%)
Median viral load at delivery (copies/ml) (*n* = 497)		87 (0–1.1 million)
Viral load at delivery (copies/ml) (*n* = 497)	Undetectable (<400)	333 (67%)
	400–9,999	132 (27%)
	10,000–49,999	11 (2%)
	≥50,000	21 (4%)
**Infants delivery characteristics (** ***n*** ** = 511)**		
Infant status	Live births	502
	Stillbirths	9 (all singletons)
	Singletons	481
	Twins	18 (9 sets)
	Triplets	3 (1 set)
Infant sex	Female (8 stillbirths)	235 (46%)
	Male (1 stillbirth)	276 (54%)
Delivery method (*n* = 511)	Spontaneous vertex	386 (76%)
	Breech	8 (2%)
	Cesarean delivery	41 (8%)
	Other/unknown	76 (15%)
Median infant birth weight (g) (*n* = 493)		3,000 (1,000–4,700)
Infant birth weight (g) (*n* = 493)	<2,500	48 (10%)
	≥2,500	420 (90%)

Percentages were computed excluding individuals with missing data for that particular characteristic.

a New Nyanza Provincial General Hospital and Kisumu District Hospital.

TBA, traditional birth attendant.

### Transmission Rates

**Figure 2:**
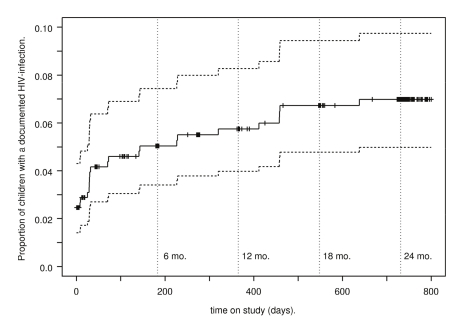
Kaplan-Meier estimates of HIV-transmission rates. Kaplan-Meier estimates of HIV-transmission rates among live-born singletons and first-born infants of mothers who received triple-ARV prophylaxis between 34 wk gestation and 6 mo post partum with 95% CIs.

Among the 487 infants who ever had an HIV test, 12 had positive delivery samples (0–7 d), consistent with a transmission rate of 2.5% (1.4%–4.3%) (Table 3). An additional eight infections occurred by 6 wk for a cumulative transmission rate of 4.2% (2.7%–6.4%). Cumulative transmission rates at 6, 12, 18, and 24 mo were 5.0% (3.4%–7.4%), 5.7% (4.0%–8.3%), 6.7% (4.8%–9.4%), and 7.0% (5.0%–9.7%), respectively (Figure 2). Among 491 singleton or first-born infants, the death rates at 6, 12, 18, and 24 mo were 3.7% (2.4%–5.9%), 8.9% (6.7%–11.9%), 10.0% (7.6%–13.1%), and 10.4% (8.0%–13.6%), respectively. Likewise, among these 491 infants, the combined HIV-transmission or death rates at 6, 12, 18, and 24 mo were 8.5% (6.3%–11.4%), 13.5% (10.7%–16.9%), 15.3% (12.3%–18.9%), and 15.7% (12.7%–19.4%), respectively. Conversely, HIV-free survival at 24 mo was 84.3% (80.6%–87.3%).

**Table 3:** Kaplan-Meier estimates of rates of HIV transmission and infant death.

	HIV Transmission (*n* = 487)			Infant Death (*n* = 491)			Combined Outcome (*n* = 491)		
Age	Events	Rate	95% CI	Events	Rate	95% CI	Events	Rate	95% CI
7 d	12	2.5	(1.4–4.3)	7	1.4	(0.7–3.0)	19	3.9	(2.5–6.0)
14 d	14	2.9	(1.7–4.8)	9	1.8	(1.0–3.5)	23	4.7	(3.1–7.0)
30 d	19	3.9	(2.5–6.1)	9	1.8	(1.0–3.5)	28	5.7	(4.0–8.2)
42 d	20	4.2	(2.7–6.4)	11	2.3	(1.3–4.0)	31	6.4	(4.5–8.9)
2 m	20	4.2	(2.7–6.4)	11	2.3	(1.3–4.0)	31	6.4	(4.5–8.9)
3 m	22	4.6	(3.0–6.9)	12	2.5	(1.4–4.3)	34	7.0	(5.0–9.6)
4 m	22	4.6	(3.0–6.9)	14	2.9	(1.7–4.8)	36	7.4	(5.4–10.1)
6 m	24	5.0	(3.4–7.4)	18	3.7	(2.4–5.9)	41	8.5	(6.3–11.4)
9 m	26	5.5	(3.8–8.0)	35	7.4	(5.4–10.1)	58	12.2	(9.6–15.5)
12 m	27	5.7	(4.0–8.3)	42	8.9	(6.7–11.9)	64	13.5	(10.7–16.9)
18 m	31	6.7	(4.8–9.4)	47	10.0	(7.6–13.1)	72	15.3	(12.3–18.9)
24 m	32	7.0	(5.0–9.7)	49	10.4	(8.0–13.6)	74	15.7	(12.7–19.4)

Kaplan-Meier estimates of the cumulative rates of HIV transmission, infant death, and combined HIV transmission or death at selected ages, from birth to 24 mo, KiBS, 2003–2009. Data are infant ages (in days or months), cumulative events (counts), and Kaplan-Meier estimates of various rates (%) with 95% CIs.

Over the 2-y period from birth to 24 mo, the log-rank test shows a significant difference in overall HIV transmission when stratified by maternal baseline viral load categories (<10,000, ≥10,000 copies/ml, [*p* = 0.03]), but no significant differences in the overall HIV-transmission rates when stratified by study period (*p* = 0.53), maternal baseline CD4 count categories (<500 cells/mm^3^, ≥500 cells/mm^3^, [*p* = 0.10]), and infant sex (*p* = 0.19), and by initial drug regimen among mothers with baseline CD4 count ≥250 cells/mm^3^ (*p* = 0.96), although for many of these variables the magnitude of difference increases gradually over the follow-up period (Table 4). Focusing specifically on differences at 24 mo, HIV-transmission rates were 4.3 percentage points (−0.1 to 5.7 percentage points) higher among infants whose mothers had baseline CD4 counts <500 cells/mm^3^ than those who had baseline CD4 counts ≥500 cells/mm^3^ (*p* = 0.06). No difference was detected in 24-mo HIV-transmission rates when using a CD4 cutpoint of 350 cells/mm^3^ (*p* = 0.30). Likewise, at 24 mo, HIV-transmission rates were 5.7 percentage points (0.3–9.4 percentage points) higher among infants whose mothers had baseline viral loads ≥10,000 copies/ml than those who had baseline viral loads <10,000 copies/ml (*p* = 0.01).

**Table 4:** Stratified Kaplan-Meier estimates of HIV-transmission rates among live-born singletons and first-born infants.

Stratification Variable	Category	*n* at Risk	HIV-Transmission Rates (%) (95% CI)					Log-Rank *p*-Value	Difference at 24-mo *p*-Value
			Birth	6 mo	12 mo	18 mo	24 mo		
Enrollment	July 2003–Jan 2005	233	2.1 (0.9–5.1)	5.8 (3.4–9.7)	6.7 (4.1–10.9)	7.8 (4.9–12.2)	7.8 (4.9–12.2)	0.53	0.53
	July 2005–Nov 2006	254	2.8 (1.3–5.7)	4.4 (2.5–7.8)	4.8 (2.8–8.4)	5.8 (3.5–9.6)	6.3 (3.8–10.2)	—	—
Maternal	<350	193	4.7 (2.5–8.8)	7.4 (4.4–12.1)	8.0 (4.9–12.8)	8.6 (5.3–13.6)	8.6 (5.3–13.6)	0.23	NA
Baseline	350–499	137	0.7 (0.1–5.1)	3.8 (1.6–9.0)	5.5 (2.7–11.2)	7.2 (3.8–13.4)	8.1 (4.4–14.5)	—	—
CD4 count	≥500	157	1.3 (0.3–5.0)	3.3 (1.4–7.7)	3.3 (1.4–7.7)	4.1 (1.8–8.8)	4.1 (1.8–8.8)	—	—
(Cells/mm^3^)	**<500**	330	3.0 (1.6–5.6)	5.9 (3.8–9.1)	6.9 (4.6–10.3)	8.0 (5.5–11.6)	8.4 (5.8–12.0)	0.10	0.06
	**≥500**	157	1.3 (0.3–5.0)	3.3 (1.4–7.7)	3.3 (1.4–7.7)	4.1 (1.8–8.8)	4.1 (1.8–8.8)	—	—
Maternal	<400	27	3.7 (0.5–23.5)	3.7 (0.5–23.5)	3.7 (0.5–23.5)	3.7 (0.5–23.5)	3.7 (0.5–23.5)	0.21	NA
Baseline	400–9,999	112	0.9 (0.1–6.2)	1.9 (0.5–7.2)	2.8 (0.9–8.5)	2.8 (0.9–8.5)	2.8 (0.9–8.5)	—	—
Viral load	10,000–49,999	140	3.6 (1.5–8.4)	5.8 (2.9–11.3)	6.6 (3.5–12.3)	8.3 (4.7–14.6)	8.3 (4.7–14.6)	—	—
(Copies/ml)	≥50,000	206	2.4 (1.0–5.7)	6.5 (3.8–11.0)	7.1 (4.3–11.7)	8.3 (5.2–13.2)	8.9 (5.6–14.0)	—	—
	**<10,000**	139	1.4 (0.4–5.6)	2.2 (0.7–6.7)	3.0 (1.1–7.8)	3.0 (1.1–7.8)	3.0 (1.1–7.8)	0.03	0.01
	**≥10,000**	346	2.9 (1.6–5.3)	6.2 (4.1–9.4)	6.9 (4.6–10.2)	8.3 (5.8–11.9)	8.7 (6.1–12.3)	—	—
Infant sex	Female	223	3.1 (1.5–6.5)	6.5 (3.9–10.7)	7.6 (4.7–12.1)	8.7 (5.5–13.4)	8.7 (5.5–13.4)	0.19	0.21
	Male	264	1.9 (0.8–4.5)	3.8 (2.1–7.0)	4.3 (2.4–7.6)	5.1 (3.0–8.7)	5.6 (3.4–9.3)	—	—
Initial regimen	NVP-based	177	1.1 (0.3–4.4)	5.2 (2.8–9.8)	5.9 (3.2–10.6)	7.2 (4.2–12.4)	7.2 (4.2–12.4)	0.96	0.95
(CD4≥250)	NFV-based	196	3.1 (1.4–6.7)	4.7 (2.5–8.8)	5.3 (2.9–9.6)	6.5 (3.7–11.2)	7.1 (4.2–12.0)	—	—

Data are numbers of singleton or first-born (in a multiple birth) infants initially at risk in each stratification category, Kaplan-Meier estimates of HIV-transmission rates (%) with 95% CIs at selected time points, *p*-values for the log-rank test, and, for stratification variables with two levels, two-sided *p*-values for the test of difference at 24 mo.

### Maternal Adverse Events

Of the 522 enrolled women, 66 (12.6%) had at least one ARV substituted between enrollment and 9 mo post partum (Table 5), including 42 of 310 (13.5%) women initially given NVP and 26 of 522 (5.0%) women initially given ZDV. Of these 66 women, 53 (80%) had an ARV-related SAE, and 13 (20%) had an illness that required treatment incompatible with NVP (e.g., rifampicin for tuberculosis, warfarin for anticoagulation). One participant had NFV substituted due to neutropenia. Most SAEs were due to common causes of morbidity in this region and HIV-related opportunistic infections (e.g., malaria, tuberculosis). SAEs most likely associated with study ARVs included anemia, neutropenia, hepatotoxicity, and rash. Grade 3 or 4 anemia (Hb<7.0 g/dl) and neutropenia (neutrophil count <750 cells/ml measured on two or more consecutive visits), occurred among 31 and 36 participants, respectively, and nine participants had both. The Hb concentration among most participants increased over time: Hb≤10.0 g/dl for 58% at baseline and 9% at 9 mo post partum. In most cases the neutropenia resolved without substitution of ARVs. Overall, 22 of 522 (4.2%) participants required ARV substitution because of anemia and/or neutropenia (Table 5). Hepatotoxicity (≥grade 2) SAEs occurred among 33 participants. Of the 14 with grade 3 or 4 hepatotoxicity, 12 were participants taking NVP. Among participants who initially received NVP, grade 3 or 4 hepatotoxicity occurred in 9 of 196 (5%) women with a baseline CD4 count of ≥250 cells/mm^3^ versus three of 114 (3%) women with CD4 count of <250 cells/mm^3^ (RR = 1.7, 95% CI 0.5–6.3). There were 16 rash SAEs, six with concomitant hepatotoxicity; all occurred among women on NVP. Rash SAEs occurred in 12 of 196 (6%) women with a baseline CD4 count of ≥250 cells/mm^3^ (including three cases of Stevens-Johnson Syndrome, all with CD4 counts of >350 cells/mm^3^) versus four of 114 (4%) women with CD4 counts of <250 cells/mm^3^ (RR = 1.7, 95% CI 0.6–5.3). All rash SAEs resolved after cessation of NVP. Of 12 maternal deaths (five before 9 mo), nine were attributed to opportunistic infections and three to progression of preexisting cardiac disease. No deaths were attributed to ARVs.

**Table 5:** Maternal SAEs during intervention period (enrollment to 9 mo post partum), number resulting in ARV substitution, and proportion of women initiated on ARV who required substitution.

Serious Adverse Event		Total *n* Women with SAE (*n* = 522)	Number of Women Requiring an ARV Substitution for Each SAE/Illness^a^	Number of Maternal ARV Substituted (%)		
				NVP (*n* = 310)	ZDV (*n* = 522)	NFV (*n* = 212)
**Potentially ARV-related**	Neutropenia	36	5	0	4 (0.8%)	1 (0.5%)
	Anemia	31	14	0	14 (2.7%)	0
	Anemia and neutropenia	9	3	0	3 (0.6%)	0
	Hepatotoxicity^b^	27	17	15 (4.8%)	2 (0.4%)	0
	Rash	10	8	8 (2.6%)	0	0
	Hepatotoxicity and rash^c^	6	6	6 (1.9%)	2 (0.4%)	0
**Illness requiring ARV change**	TB treatment^d^	13	10	10 (3.2%)	1 (0.2%)	0
	DVT treatment	2	2	2 (0.6%)	0	0
	Hyperbilirubinemia	2	1	1 (0.3%)	0	0
**Other SAEs**	Malaria	23	0	—	—	—
	Pneumonia	7	0	—	—	—
	Death	6	0	—	—	—
	Gastroenteritis	3	0	—	—	—
	Other	19	0	0	0	0
**Total**		194	66	42 (13.5%)	26 (5%)	1 (0.5%)

a Each participant only reported once for primary reason for ARV substitution between enrollment and 9-mo post partum.

b Includes one participant where SAE attributed to ZDV, but stopped all ARVs.

c Includes two participants who stopped both NVP and ZDV.

d Includes one participant who stopped both NVP and ZDV.

DVT, deep venous thrombosis.

### Infant Adverse Events

The most common causes of child SAEs included diarrhea, malaria, pneumonia, and anemia. Of the 146 reported diarrhea SAEs, 86 (59%) occurred between the 5- and 9-mo study visits (peri-weaning period). By 24-mo 49 (10%) of first-born children had died (two additional deaths occurred among second-born children); 42 (86%) deaths occurred during the first year of life. The three most frequent causes of death were diarrhea (35%), pneumonia (16%), and respiratory failure (12%). Twelve deaths due to diarrhea occurred during the peri-weaning period and thus could be attributed to early weaning; two of these infants were HIV positive. No child deaths or other SAEs were clearly attributable to maternal or child ARVs.

### Adherence to Regimen

Of the 522 enrolled participants, 439 (84%) took triple-ARV prophylaxis through 6 mo post partum, whereas 83 (16%) stopped prematurely due to withdrawal (56%), infant death/stillbirth (19%), breastfeeding cessation (7%), maternal death (5%), noncompliance with drugs/study visits (5%), and other reasons (7%). Among participants on study at 6 mo, 82% (359/439) were ≥95% adherent to the study ARVs. Of those participants with viral load testing results, 5% (27/520) at enrollment, 67% (333/497) at delivery, and 80% (348/435) at 6 mo post partum had an undetectable viral load (defined as <400 RNA copies/ml). Among the 333 participants with an undetectable viral load at delivery, 88% (263/298) at 14 wk and 89% (258/294) at 6 mo post partum (79% [227/288] at both times) had an undetectable viral load.

Among live-born infants, 98% (494/502) received a single dose of NVP at delivery. Mixed feeding before 5 mo was documented for 22% (109/502) of live-born infants. The triplets were never breastfed but were provided formula. Of the HIV-negative infants on study at 6 mo, 87% (379/434) reportedly had stopped breastfeeding by 6 mo, in accordance with study recommendations. Nine infants who tested HIV negative at 6 mo subsequently became HIV infected; none of their mothers, who had been advised to stop breastfeeding, were currently receiving ARVs. When probed about possible causes of infection only two of these mothers acknowledged breastfeeding their infants beyond 6 mo.

## Discussion

### Transmission Rates and Comparison to Other Trials

The KiBS achieved 6-wk and 18-mo HIV-transmission rates of 4.2% and 6.7%, respectively. These rates are less than half the corresponding HIV-transmission rates of 11.8% and 15.7% observed in the HIVNET 012 study [8] conducted in Uganda, using single-dose maternal and infant NVP. Likewise, KiBS achieved a 4-mo transmission rate of 4.6%, a 77% reduction compared with the corresponding rate of 19.9% reported in a study of the impact of maternal malaria on perinatal HIV transmission [19], conducted in Kisumu between 1996 and 2001 without a PMTCT intervention. Several studies have reported comparable 6-mo HIV-transmission rates to that of KiBS (5.0% [3.4%–7.4%]). MITRA Plus [20] in Tanzania, which provided maternal ZDV, 3TC, and either NVP or NFV during late pregnancy through 6 mo of breastfeeding (infants received ZDV+3TC for 1 wk after birth), reported a 6-mo transmission rate of 5.0% (3.2%–7.0%). Two recently completed randomized trials also reported similar transmission rates in the arms comparable to KiBS. In one arm of the BAN study in Malawi [21], breastfeeding mothers with CD4 counts ≥250 cells/mm^3^ and their infants received a short-course regimen including single-dose NVP at birth and ZDV and 3TC for 7 d and then mothers received a maternal triple-ARV regimen (Combivir and either NVP or a protease inhibitor) for 28 wk. At 28 wk post partum the transmission rate among all infants randomized to this arm was 8.2% (6.5%–10.3%). The Kesho Bora study [22] randomized women at 28–36 wk gestation, with CD4 counts 200–500 cells/mm^3^ to either a triple-ARV regimen (ZDV+3TC+Lopinavir/ritonavir [Kaletra, Abbott]) or a short ARV regimen (ZDV through delivery plus single-dose NVP in labor). The 6-mo transmission rate in the triple-ARV arm was 4.9% (3.1%–7.5%). Two other studies using triple-ARV regimens have shown somewhat lower rates. The AMATA study in Rwanda [23], which provided maternal triple ARVs from 28 wk gestation and for up to 6 mo of breastfeeding, reported a 9-mo transmission rate of 1.8% (0.7%–4.8%). The Mma Bana study in Botswana [24], which provided maternal triple ARVs from 28 to 34 wk gestation through 6 mo of breastfeeding, reported a 6-mo transmission rate of 1.1% (0.5%–2.2%).

In KiBS, 62.5% of the infant infections occurred by 42 d, i.e., primarily in utero or intrapartum. The median duration on ARVs antepartum was 5.6 wk and 33% of tested participants had not achieved undetectable viral load at delivery. The duration on ARVs antepartum may explain some of the differences in transmission rates seen in the PMTCT studies mentioned earlier. The Mma Bana [24] and the AMATA study [23] reported median duration on ARVs antepartum of 11 wk and 16.4 wk, respectively, and both had 6-wk transmission rates ≤1.3%. Mitra Plus [20] reported a median duration on ARVs antepartum of 5.4 wk and a 6-wk transmission rate of 4.1%, very similar to KiBS (4.2%). Kesho Bora [22] reported 42.6% of the participants receiving <6 wk of triple ARVs antepartum and a transmission rate of 3.3%. Given these findings and the transmission rates below 2% in countries where triple-ARV prophylaxis is initiated during the second trimester and where breastfeeding is avoided [25], it is likely that initiating ARVs earlier during pregnancy in this study would have further reduced in utero and intrapartum transmission However, timely initiation of ARVs may be a challenge in areas where many women present late for antenatal care. A 2002 study of recently delivered women in rural western Kenya showed that 64% first visited the antenatal clinic in the third trimester [26].

The cumulative transmission between 6 wk and 24 mo, probably attributable to breastfeeding, was 3.2% (95% CI 1.9%–5.5%). Nine infants became infected after 6 mo post partum. We probed the mothers for possible mechanisms of late infection; seven of them denied any breastfeeding beyond 6 mo, two infants underwent a traditional scarring procedure, and no mothers reported premastication of food or breastfeeding by other women (neither of these practices was common among KiBS participants). Thus, unreported continued breastfeeding remains the most likely mechanism of late infection. Cessation of breastfeeding may have been difficult because of insufficient resources, fear of stigma and unintentional disclosure, and the tradition of breastfeeding well beyond 6 mo. Family support and cultural norms, which are important to a mother's decision about when to stop breastfeeding, should be considered when promoting early cessation of breastfeeding [27]. Rapid weaning may also have contributed to infant HIV infection because of its association with elevations in breast milk HIV levels and mastitis, as observed in the Zambia Exclusive Breastfeeding Study [28]. The revised WHO treatment criteria recommend treatment for those with CD4 counts <350 cells/mm^3^ or WHO stage 3 or 4. Whereas we observed lower 24-mo HIV-transmission rates among infants whose mothers had higher CD4 count levels (≥500 cells/mm^3^), we did not find any differences in infant transmission rates for those with maternal baseline CD4 counts between 350–499 cells/mm^3^ and those with CD4 counts <350 cells/mm^3^.

The rates of infant HIV transmission or death at 6, 12, and 18 mo in KIBS were 8.5% (6.3%–11.4%), 13.5% (10.7%–16.9%), and 15.3% (12.3%–18.9%), respectively. These rates are similar to those reported at the same time points in MITRA Plus [20], namely 8.6% (6.0%–11.2%), 12.8% (9.6%–16.0%), and 13.6% (10.3%–16.9%). By comparison, the rate of HIV transmission or death at 18 mo in HIVNET 012 [8] was 20.7% (16.2%–23.8%). Although the study met the objective of a 50% reduction in transmission compared to HIVNET 012 historic data, a corresponding reduction in the combined endpoint of transmission or death was not achieved. The failure to achieve this latter reduction may reflect differences in study location, population, and access to medical care among other possible factors.

### Adherence and Adverse Events

The KiBS study intervention appeared well tolerated and safe. Most (84%) of those enrolled took ARVs until 6 mo post partum, even though most did not meet treatment criteria for themselves. The implications of initiating triple ARVs when not needed for their own health and then stopping ARVs after several months require further study. Women who participated in this trial are currently being enrolled into a study to evaluate their subsequent response to ARV treatment. The development of ARV resistance among infants is reported elsewhere in this issue [29]. Of those who completed the intervention, 82% were ≥95% adherent. Furthermore, an evaluation of viral load levels demonstrated that for most women whose viral load was suppressed at delivery, suppression was maintained at 6 mo post partum. There were no unexpected ARV-related SAEs, although infant gastroenteritis SAEs increased during the weaning period. The rates of NVP- and ZDV-related SAEs were generally consistent with those reported in the prescribing information from the drug manufacturers [17],[18], although we did not find a significant increase in NVP-related hepatotoxicity and rash among mothers with CD4 counts above 250 cells/mm^3^ as had been reported in nonpregnant women in other trials. Our findings are consistent with findings from a recent analysis of HIV-infected pregnant women on ARVs [30] in the US where a significant differential by CD4 count was likewise not evident. Observed neutropenia was attributed primarily to lower normal physiologic levels for African women [31]; the use of US-based toxicity tables may have resulted in overestimation of neutropenia-related SAEs.

### Advantages

One advantage of this study was the use of a simple regimen that did not require different drugs at different time points in the pregnancy-delivery-breastfeeding continuum, and which could be provided to all HIV-infected women regardless of CD4 count. In many resource-limited settings one may not have the time to wait for a CD4 count before deciding what regimen to start for PMTCT. While the study regimen was changed in response to concerns about NVP toxicity, our subsequent analysis shows that women with higher CD4 counts on NVP treatment are not at increased risk compared to women with lower CD4 counts.

### Limitations

A limitation of this study is the lack of a concurrent control group. This decision was based on cost constraints and anticipated difficulty in enrolling adequate numbers for a randomized trial. Another limitation impacting the generalizability of this study includes the intensity of visits, particularly during the intervention period. The Kenya Ministry of Health currently schedules vaccinations and vitamin A dosing in children ≤2 y of age at birth, 6, 10, and 14 wk, and 6, 9, 12, 18, and 24 mo [32]. The study visit schedule was initially designed to follow the vaccination schedule with only two additional visits at 2 wk and 15 mo. We modified the protocol in 2005, adding visits at 5, 7, and 8 mo because of concerns about the increased incidence of diarrhea during the peri-weaning period. These visits focused on infant feeding, weight, and health. No testing for HIV was done at the 10-wk, and 5-, 7-, and 8-mo visits. Other limitations include the difficulty in accurately assessing gestational age, the predominantly urban population, participant self-selection bias, and possibility that some participants may have given socially desirable responses to questions about adherence to ARVs or infant feeding recommendations because of the strong emphasis on these issues by study staff.

### Summary

The KiBS demonstrates the feasibility and safety of the use of triple-ARV prophylaxis from 34–36 wk gestation through 6 mo of breastfeeding for PMTCT and achieved low transmission rates among HIV-positive women who choose to breastfeed in Kisumu, Kenya, a resource-limited setting. This study reinforces the findings of other similar trials. The study findings were presented at the WHO expert consultation on new and emerging evidence on the use of ARV drugs for PMTCT in November 2008 [33] and have added to the body of evidence supporting the recent WHO guidelines [34], which recommend either the mother receiving triple ARVs or the infant receiving ARV prophylaxis. Further studies are still needed to determine the most appropriate strategies and optimal length of breastfeeding; these questions are being considered in the large PROMISE study (NIH/IMPAACT) [35]. Promoting HIV testing among pregnant women and expectant fathers and ensuring that those who test positive receive effective and timely PMTCT services and treatment remains a critical worldwide challenge; however, the demonstration of effective interventions now provide greater leverage for women to test and enroll into PMTCT programs. In addition, the greater availability of ARVs, especially combination pills that simplify regimens, vastly improves the prospects for widespread implementation of triple ARVs during pregnancy and breastfeeding for PMTCT in sub-Saharan Africa, the heart of the current AIDS pandemic.

## Supporting Information

Text S1 Protocol.
(PDF)

Text S2 CONSORT Checklist.
(DOC)

## Abbreviations

3TC: lamivudine
ARV: antiretroviral
CI: confidence interval
Hb: hemoglobin
KiBS: Kisumu Breastfeeding Study
NFV: nelfinavir
NVP: nevirapine
PMTCT: prevention of mother-to-child transmission
SAE: serious adverse event
ZDV: zidovudine
