# Supplementary material for: Triple-Antiretroviral Prophylaxis to Prevent Mother-To-Child HIV Transmission through Breastfeeding—The Kisumu Breastfeeding Study, Kenya: A Clinical Trial
Source: PLoS Med. 2011 Mar 29;8(3):e1001015. doi: 10.1371/journal.pmed.1001015 (PMC3066129; doi:10.1371/journal.pmed.1001015)
Supplement: Text S1 — Protocol. (PDF) [file pmed.1001015.s001.pdf]

A PHASE II OPEN LABEL CLINICAL TRIAL OF MATERNAL ZIDOVUDINE/LAMIVUDINE AND EITHER NEVIRAPINE OR NELFINAVIR FOR MAXIMAL REDUCTION OF MOTHER–TO-CHILD HIV TRANSMISSION IN RESOURCE LIMITED SETTINGS AMONG BREASTFEEDING POPULATIONS

[Amendment 10]

Sponsored by: The Centers for Disease Control and Prevention, KEMRI and Kenyan Ministry of Health

Principal Investigator: Timothy Thomas[1]

Co-Principal Investigators: Pauli Amornkul[1], Dorothy Mbori-Ngacha[2]

Collaborators: Kevin De Cock[10], Larry Slutsker[6], John Vulule[4], Juliana Otieno[3], Ambrose Misore[5]

KEMRI/CDC Kenya Co-investigators: Clement Zeh[1], Rose Masaba[4]

CDC Atlanta Co-Investigators, Medical Officers, and Project Coordinator: Michael Thigpen[6], Craig Borkowf[6], Allan Taylor[6], Paul Weidle[6], Eleanor McLellan[6]

Consultants: Annemieke van Eijk[1], Barbara Marston[6], Denise Jamieson[6], Marc Bulterys[6], Douglas Taren[7], Mark Mirochnick[8], Mary Glenn Fowler[9] Larry Marum[6], Anja van't Hoog[1] Dan Rosen[6], Ya Ping Shi[6], Robert Quick[6], John Odondi[3], Christopher Oyoo[3]

1 Centers for Disease Control and Prevention, Kisumu, Kenya
2 Centers for Disease Control and Prevention, Nairobi, Kenya
3 Provincial Hospital, Kisumu, Kenya
4 Kenya Medical Research Institute, Kisumu, Kenya
5 Kenya Ministry of Health, Provincial Medical Office, Nyanza
6 Centers for Disease Control and Prevention, Atlanta, GA, USA
7 University of Arizona
8 Boston University
9 Makerere University Johns Hopkins University, Kampala, Uganda
10 World Health Organization

Funding Source: Centers for Disease Control and Prevention (CDC)

1. ABSTRACT **4**
2. SCHEMA/ENDPOINTS **6**
3. INTRODUCTION/BACKGROUND **10**
4. JUSTIFICATION **37**
5. HYPOTHESES **42**
6. OBJECTIVES **42**
6.1 PRIMARY OBJECTIVES 43
6.2 SECONDARY OBJECTIVES 43
7. DESIGN AND METHODOLOGY **45**
7. 1 STUDY SITE DESCRIPTION 45
7.2 STUDY POPULATION 46
7.2.1 Study Inclusion Criteria-Women *49*
7.2.2 Study Exclusion Criteria-Women *50*
7.2.3 Infants *52*
7.3 SAMPLING 52
7.4 PROCEDURES (*SEE APPENDIX III FOR ENROLLMENT FLOW DIAGRAM*) 53
7.4.1 *Study Treatment – Drug Regimen* *55*
7.4.2 Drug Dosage and Administration *56*
7.4.3 Drug Formulation *59*
7.4.4 Concomitant Medications and Basic Package of Prenatal and Infant Care *60*
7.4.5 Criteria for Holding Intrapartum Study Drug Dosing or for Study Discontinuation *61*
7.4.6 Evaluations *63*
7.4.7 Sample Storage and testing *70*
7.4.8 Biohazard containment *71*
7.4.9 Counseling *71*
7.4.10 Weaning *72*
7.4.11 Completion of study evaluations *73*
7.4.12 Follow-up *74*
7.4.13 Consent Process *75*
7.4.14 Toxicity Management of Mothers and Infants *76*
7.4.15 Criteria for Discontinuation of ARVs (Mother and Neonate) *85*
8. DATA MANAGEMENT **86**
8.1 QUALITY CONTROL 87
8.2 DATA ANALYSIS 89
8.3 DATA MONITORING 95
8.4 ADVERSE EVENT REPORTING 96
9. TIME FRAME/DURATION OF PROJECT **98**
10. ETHICAL CONSIDERATIONS **99**
11. EXPECTED APPLICATIONS OF THE RESULTS **109**
12. REFERENCES **110**
APPENDICES **115**
KEY FACTS SHEET **115**
SCHEDULED VISITS FOR MOTHER **117**
SCHEDULED VISITS FOR BABY **118**
MAIN STUDY CONSENT FORM **119**

MAIN STUDY ASSENT FORM 129
BREAST MILK SUBSTUDY 138
BREAST MILK SUBSTUDY CONSENT FORM 143
BREAST MILK SUBSTUDY ASSENT FORM 148
GUIDE TO HYGIENE DISCUSSION WITH KIBS MOTHERS 153
STUDY DRUG INFORMATION SHEET 154
NELFINAVIR RECALL INFORMATION 161
CONSENT FOR CONTINUED FOLLOW UP FORM 163

1. Abstract

Background: Approximately 800,000 HIV-infected infants are born each year, two thirds of them in sub-Saharan Africa.[1] The rate of HIV transmission from mother to infant is estimated at 13-48% in the absence of antiretroviral treatment. Interventions currently available to prevent mother to child transmission in resource-limited settings among breastfeeding populations are few and only partially effective.[2,3]. Because safe alternatives to breastfeeding are not currently a viable option for many HIV-infected women in sub-Saharan Africa, it is important to identify interventions to decrease transmission to the infant during this period.

Objective: To demonstrate that a regimen using highly active antiretroviral therapy (HAART) to maximally suppress maternal viral load in the late antenatal period and during the first six months of lactation is safe, effective and can be implemented in resource poor settings in order to reduce the risk of HIV transmission to the infant.

Study Design: The study will be an open label Phase II trial of Zidovudine/Lamivudine and depending on maternal CD4 count, either Nevirapine or Nelfinavir to assess the safety, tolerance and activity of maternal HAART to reduce the risk of transmission among breastfeeding HIV infected women in Kisumu Kenya[2,3].

Justification: Transmission through breastfeeding accounts for 25-40% of all mother-to-child transmission of HIV. This trial will assess the safety and activity of a potent triple drug combination to lower maternal viral load prenatally, intrapartum and during breast feeding. The regimen will be given to HIV infected women from 34 weeks gestation through 6 months of breastfeeding postpartum. Infants will receive a single dose of NVP. Women will be encouraged to breastfeed exclusively and wean abruptly at 6 months. Mother and infant will be followed for 24 months. Clinical and laboratory evaluations will be performed periodically to determine infection status of the infant and side effects of medications to

mother and infant. The estimated sample size needed to address both transmission reduction of HAART in comparison to single dose NVP as well as related safety of the two HAART regimens is 520 mother-infant pairs. Comparison will be made with data from previous studies (HIVNET O12 in Uganda and the malaria vertical transmission study in Kisumu) and with data from current Prevention of Mother to Child Transmission (PMCT) programs in Kisumu.

Expectations and significance: A HAART regimen of this nature may potentially show a dramatic decrease in transmission when compared to existing PMCT regimens. Given the continued decreasing costs of ARVs and the relatively low efficacy of existing PMCT regimens, such a regimen would be seriously considered for wide implementation in resource poor settings and elsewhere.

2. SCHEMA/ENDPOINTS

A PHASE II OPEN LABEL CLINICAL TRIAL OF MATERNAL ZIDOVUDINE/LAMIVUDINE AND NEVIRAPINE FOR MAXIMAL REDUCTION OF MOTHER–TO-CHILD HIV TRANSMISSION IN RESOURCE LIMITED SETTINGS AMONG BREASTFEEDING POPULATIONS

Design: The study will be an open label Phase II trial of maternal Zidovudine/Lamivudine and either Nevirapine or Nelfinavir given to HIV-1-infected mothers from 34 weeks gestation through 6 months of breastfeeding postpartum.

Population:

Mothers: HIV-1-infected pregnant women in Western Kenya who meet eligibility criteria and agree to take part in the trial.

Neonates: Born to HIV-1-infected mothers enrolled in the study.

Sample Size: **520** evaluable mother- infant pairs (takes into account 10% loss to follow up)

Treatment Regimen

ZDV = Zidovudine (all women)
3TC = Lamivudine (all women)
NVP = Nevirapine (for women with CD4<250/mm^3 at screening)
NLF= Nelfinavir (for women with CD4>250/mm^3 at screening)

Antenatal Regimen (34-36 weeks to onset of labor) for women with CD4 < 250/mm^3 at screening		
	Combivir Dosing Regimen	*Nevirapine Dosing Regimen*
Mother	1 Tablet BID Combivir (150mg 3TC and 300mg ZDV)	Initial 2 week lead in with 200mg NVP po QD; thereafter NVP po 200 mg BID
Labor, delivery and neonatal period		
	Combivir Dosing Regimen	*Nevirapine Dosing Regimen*
Mother<br><br>Infant	1 Tablet BID Combivir (150mg 3TC and 300mg ZDV)	NVP po 200 mg BID<br><br>One dose 2 mg/kg NVP suspension po within 72 hours of birth (up to 7 days postpartum)
Postpartum Regimen (for 6 months unless infant weaned before 6 months)*		
	Combivir Dosing Regimen	*Nevirapine Dosing Regimen*
Mother	1 Tablet BID Combivir ** (150mg 3TC and 300mg ZDV)	NVP po 200 mg BID

* Mothers who meet WHO criteria for treatment will continue drug treatment and be referred post-study.
** Mothers who stop drugs at 6 months will continue Combivir for 21 days beyond the last NVP dose in order to cover the long half life of NVP.

Antenatal Regimen (34-36 weeks to onset of labor) for women with CD4 > 250/mm³ at screening		
	Combivir Dosing Regimen	***Nelfinavir Dosing Regimen***
Mother	1 Tablet BID Combivir (150mg 3TC and 300mg ZDV)	1250 mg po BID
Labor, delivery and neonatal period		
	Combivir Dosing Regimen	***Nefinavir Dosing Regimen***
Mother<br><br>Infant	1 Tablet BID Combivir (150mg 3TC and 300mg ZDV)	NLF po 1250mg BID<br><br>One dose 2 mg/kg NVP suspension po within 72 hours of birth (up to 7 days postpartum)
Postpartum Regimen (for 6 months unless infant weaned before 6 months)*		
	Combivir Dosing Regimen	***Nelfinavir Dosing Regimen***
Mother	1 Tablet BID Combivir (150mg 3TC and 300mg ZDV)	NLF po 1250 mg BID

* Mothers who meet WHO criteria for treatment will continue drug treatment and be referred post-study.

Study duration: **48-54** months
Enrollment: **24-30** months
Follow-up:
Neonates/infants: 24 months post birth
Mothers: 24 months postpartum

Primary Endpoints:

1. The cumulative risk of infection in infants born to study participants, determined at 6 weeks, 9 months, and 18 months of age. Infection to be determined by a positive plasma HIV-1 DNA-PCR result and confirmed by a second positive HIV-1 DNA-PCR or plasma HIV-1 RNA PCR on a different specimen for infants <18 months of age. Infants ≥ 18 months of age will be considered infected if two separate EIA's are positive; (and considered negative if two EIA's are negative and breastfeeding has ceased at least 3 months prior).
2. Infant HIV-free survival rates at 24 months.

3. Safety and tolerance of ZDV, 3TC, and NVP or NLF given to women beginning at 34-36 weeks gestation through 6 months of breastfeeding and safety of infant exposure to maternal antiretrovirals through maternal breast milk.

Secondary Endpoints:

1. **Infant survival (regardless of HIV infection status) at 24 months**
2. **Changes in maternal viral load and CD4 counts from initiation of HAART, prior to delivery to 6 and 14 weeks, 6, 9, 12, 18 and 24 months post- partum; and the relationship of maternal CD4 and viral load at these time points to risk of transmission post partum**
3. **Rates of serious adverse events requiring change of drug for Nevirapine compared to Nelfinavir among women with CD4 counts >250/mm^3 enrolled into the KiBS trial.**
4. **Rates and types of antiretroviral resistant mutations in the plasma of study women and in the plasma of their infected infants at delivery, 6 weeks, 14 weeks, 6, 9, 12, 18 and 24 months postpartum.**
5. **Maternal acceptance of and adherence to antiretroviral regimens based on self-report measures, pill count and use of electronic medication vials.**
6. **Maternal acceptance of and adherence to exclusive breastfeeding based on response to a modified UNAIDS feeding questionnaire.**
7. **Negative and positive social consequences for mothers taking HAART based on maternal self- report questionnaire.**
8. **Breast milk sub study: Assess viral load, resistance patterns, and drug levels in maternal blood and breast milk at delivery, 2 weeks, 6 weeks, 14 weeks, and 6 months based on a sub study of 150 women and infants receiving HAART followed for 24 months postpartum who agree to participate in the sub study.**

9. **Infant growth, stratified by infant infection status, during breastfeeding and following weaning.**
10. **Maternal nutritional status using height, weight and mid upper arm circumference measured within first week after delivery and then at 6 weeks, 14 weeks, 6, 9, 12, 18 and 24 months postpartum.**
11. **Maternal mortality rates at delivery, 6 and 14 weeks, 6, 9, 12, 18 and 24 months using hospital records and/or information from relatives.**

3. INTRODUCTION/BACKGROUND

Literature Review and Current State of Knowledge

The HIV pandemic in sub-Saharan Africa has resulted in maternal seroprevalence rates of 15-30% in many parts of Eastern and Southern Africa and continues to infect women in increasing proportions.[1] The rate of HIV transmission from mother to infant is estimated at 13-48% in the absence of antiretroviral treatment.[1,2,3] Because most cases of pediatric HIV-infection are the result of mother-to-child-transmission, HIV prevalence among children is also on the rise. Over two-thirds of the nearly 700,000 HIV-infected infants born each year reside in sub-Saharan Africa. [1,4, 5]

Transmission through breastfeeding is estimated to account for one third to one half of all mother-to-child transmission of HIV.[1,5-9] However, few data exist on effective strategies to prevent mother-to-child HIV transmission during the breastfeeding period. Because safe and culturally acceptable alternatives to breastfeeding are not currently a viable option for many HIV-infected women in sub-Saharan Africa, it is important to identify interventions to decrease HIV transmission to the infant during the breastfeeding period. Thus, one of the unanswered and urgent research challenges in resource-limited settings is the prevention of HIV transmission during breastfeeding.

Reducing Perinatal HIV Transmission: Antiretroviral Trial Results to Date

Based on the PACTG 076 trial results,[10] an intense regimen of Zidovudine (ZDV) was demonstrated to reduce vertical transmission by approximately 67% in non-breastfeeding settings. In this randomized blinded trial, Zidovudine or placebo was given to pregnant women starting at 14-34 weeks gestation, during labor and then to the infant for 6 weeks. A study in

Thailand,[11] which gave a short course of ZDV starting at 36 weeks and then during labor only, reduced transmission by 50% among non-breastfeeding women. In resource limited settings where women generally breast feed through infancy, short course antenatal ZDV, ZDV/3TC and single dose Nevirapine given to the mother and infant can reduce early transmission by about 40%.[4] Follow up to 18-24 months showed the long-term effectiveness of the short course ZDV and other ZDV regimens was reduced by ongoing transmission through breastfeeding; the efficacy of Nevirapine was maintained at about 40%.

In two short course Zidovudine trials in West Africa among breastfeeding women in Cote d'Ivoire and Burkina Faso, HIV seropositive pregnant women were randomized to receive placebo or ZDV starting at 36 weeks, and continuing during labor and delivery and also for 7 days postpartum in one of the two trials . In a pooled analysis,[12] the estimated risk of transmission in the placebo and ZDV groups respectively were 24.8% and 14.7% at 6 weeks, and 26.1% and 16.8% at 3 months, showing an early efficacy of 41% at 6 weeks and 36% at 3 months. Long-term follow-up at 24 months showed a transmission rate of 22.5% and overall late efficacy of 26% which was primarily seen among women with CD4 counts >500 cells/mm^3 at delivery.

The PETRA trial evaluated the efficacy of short course ZDV/3TC for the prevention of perinatal transmission. In a factorial trial design carried out in South Africa, Tanzania and Uganda, HIV-infected women were randomized to one of the following 4 intervention arms:

	Pre-partum	Intra-partum	Post-partum
Arm A	ZDV / 3TC	ZDV / 3TC	ZDV / 3TC
Arm B	Placebo	ZDV / 3TC	ZDV / 3TC
Arm C	Placebo	ZDV / 3TC	Placebo
Arm D	Placebo	Placebo	Placebo

A total of 1,792 women were enrolled in the PETRA study and 1457 subjects were included in the analyses presented at the 2001 Global Strategies Meeting in Kampala. At 6 weeks and 18 months postpartum, the following transmission rates were reported based on DNA PCR:

	Transmission Rate at 6 weeks	Rate at 18 months
Arm A	5.9 %	14.9%
Arm B	8.9 %	18.1%
Arm C	14.2 %	20.3%
Arm D	15.3 %	22.2%

Follow up transmission rates at 18 months in the PETRA study[13] revealed statistical loss of efficacy for the treatment arms although the trend continued for reduced transmission in arms A and B compared to placebo. Late transmission occurring between 6 weeks and 18 months and presumably related to breast-feeding transmission ranged between 6 to 9% across study arms.

The HIVNET 012 study[14] compared single dose NVP to Ugandan mothers and their neonates to intrapartum maternal ZDV followed by 1 week of ZDV to the infant postpartum. Administration of a single oral 200 mg dose of NVP to the mother at onset of labor and a single 2 mg/kg dose to the infant within 72 hours after birth resulted in a 42% reduction in perinatal transmission rate at 6 weeks compared with a short course of ZDV (600 mg orally plus 300 mg every 3 hrs thereafter during labor and 4 mg/kg orally twice a day to her infant for 7 days after birth). Final transmission results at 6 weeks, and 18 months for the ZDV and NVP groups were as follows:

Estimated Transmission Rates

	NVP (N=309)	ZDV	**Relative NVP Efficacy to short course ZDV**
**(N=307)**			
by day 3	8.2%	10.4%	--
at 6-8 weeks	11.8%	20.3%	42%
at 14-16 weeks	13.5%	22.1%	47%
18 months	15.7%	25.8%	41%

Later transmission occurring between 6 weeks and 18 months was 3.8% for the NVP arm and 5.5% for the ZDV arm.[15]

In 2004, data from an efficacy trial found that ZDV given from 28 weeks gestation plus single dose NVP to the mother and neonate plus one week ZDV to the infant was associated with a transmission rate of 2% among non breast feeding HIV infected women in Thailand. Another trial supported by CDC and the Thai MOH reported a transmission rate of 4.9% when women began ZDV at 34 weeks followed by single dose NVP to the mother and neonate, and 1 week ZDV to the infant. Likewise, results from the West Africa perinatal DITRAME Plus trial which gave short course ZDV given from 34 weeks, single dose NVP to the mother and newborn followed by 1 week of ZDV to the infant reported a transmission rate of 5.9% at 6 weeks. In that study about half of the women breast fed. These three trial results support the use of maternal combination antiretrovirals for PMCT.

HIV Transmission via Breastfeeding

Studies in breast-feeding settings have estimated the rate of vertical transmission of HIV from 25-40%, depending on the timing of testing and differing definitions of when transmission occurred; overall it is estimated that one third to one half of transmission occurs via breastfeeding. Bertolli *et al.*[8] monitored infants born to HIV-infected mothers in Kinshasa, Zaire and reported overall vertical transmission rates as 25%: 6% in utero, 18% intrapartum/early postnatal, and 4% late postnatal. A study by Ekpini *et al.* [9] in Cote d'Ivoire reported the rate of transmission attributable to breastfeeding to be 24% among infants by 6 months of age. An additional 7% seroconverted after 6 months. The estimated probability of late (>6months) postnatal transmission with account taken for loss to follow-up and observed pattern of weaning was 12% for all children and 20% for those that were breastfed for at least 24 months. A meta-analysis[16] of studies from four developing countries found that late (>2.5 months) postnatal transmission occurred in 5% of 902 children. For the 20 children for whom complete data were available, all 20 cases of late postnatal transmission could have been prevented if weaning from breastfeeding had occurred at 4 months of age. Additionally, weaning at 6 months of age would have prevented 17 of the 20 transmissions. Such studies indicate that early weaning may be a potentially effective intervention to decrease the risk of HIV transmission through breastfeeding

Two recent studies suggest that a large proportion of transmission through breast milk occur within the first 6-weeks of life. In the Nairobi, Kenya trial,[17] HIV-infected women were randomized to breastfeeding and non-breastfeeding groups. The overall rate of HIV-1 transmission via breast milk was 16.2%; 63% of the difference in transmission between breastfeeding and formula arms occurred by 6 weeks postpartum and 75% by 6 months. Breastfeeding accounted for 44% of all HIV-1 transmissions in the breastfeeding group.

Likewise, the SAINT trial[18] in South Africa which compared intrapartum and one week post partum ZDV/3TC to single dose NVP intrapartum and to the neonate also evaluated differences in transmission between birth and 8 weeks for breast fed versus formula fed infants. The investigators found a 5.6% lower transmission rate at 8 weeks between formula fed compared with breast fed infants. This again supports a high rate of transmission in the first 6-8 weeks of lactation.

Moreover, a study in Malawi[19] estimated the monthly hazard rates for HIV transmission at 0.7% during months 1-5 months postpartum, 0.6% during 6-11 months postpartum, and 0.3% during 12-17 months postpartum[22]. Taken together, these results suggest a high rate of transmission in the early months of lactation. Thus, substantial reductions of post-natal transmission through breastfeeding could be achieved either by use of breast milk substitutes from birth, very early weaning or use of antiretrovirals to reduce viral load in breast milk or to prophylax the infant during lactation. In areas where very early replacement feeding is not an option, use of maternal antiretroviral therapy during the first 4- 6 months of life to lower viral load during breastfeeding followed by weaning around 6 months could substantially reduce the risk of mother-to-child HIV transmission through breast milk.

Breastfeeding substitution and early weaning may not be currently feasible for the majority of HIV- infected women in rural sub-Saharan Africa and might potentially lead to increased infant morbidity and mortality from other causes such as diarrhea because of the lack of clean, safe water to prepare formula or use of breast milk substitutes with inadequate nutrition.[20] The benefits of decreasing the risk of HIV transmission must, therefore, be weighed against other risks associated with artificial feeding, particularly in countries with high background rates of infant

mortality from infectious diseases compounded by malnutrition. Mathematical modeling has been used to evaluate the risk of HIV transmission from breastfeeding versus the risk of all cause mortality from not breastfeeding. Several models similarly concluded that in communities with high baseline rates of infant mortality, breastfed infants have a lower risk of child mortality than their non-breastfed counterparts. However, the optimal time to abruptly wean from breastfeeding ranged from 3-7 months depending on the assumptions made in the models. More recently, data from South Africa[21] demonstrate that exclusive breastfeeding for 6 months postpartum carried a lower risk than mixed feeding where breast milk was supplemented with other liquid or solid feeds.

The Joint Policy Statement on HIV and Infant Feeding,[22] issued in 1996 by UNAIDS, UNICEF, and WHO, and updated in 1998 and October 2000, [23,24] recognized the need for appropriate alternatives to breastfeeding for HIV-infected women while continuing to promote breastfeeding for women who are HIV-negative or of unknown HIV status. The guidelines recommend that HIV-infected women be informed about the risk associated with breastfeeding and HIV transmission to allow the women to make informed decisions with respect to feeding their infant. For HIV-infected women who choose to and can safely use breast milk substitutes, this should be recommended; otherwise exclusive breastfeeding is recommended during the first months of infancy. To minimize HIV transmission, breastfeeding should be discontinued as soon as feasible given the woman's individual situation. Specific guidance and support should be given to HIV-infected women who choose not to breast feed or stop breastfeeding, to ensure that adequate replacement feeding is available. For HIV-infected women in resource-limited settings who do choose to breast feed, counseling and information on exclusive breast-feeding, nutrition and breast care should be provided, and the women should be supported in their decision but encouraged to wean early. The availability, safety, efficacy, and sociocultural appropriateness of

each option will need to be thoroughly considered by governments in order to assist mothers in effectively and safely implementing their choice of infant feeding method.

Rationale for Proposed Study

Efforts to minimize HIV transmission to the infant throughout the breastfeeding period while allowing the infant to continue to receive the benefits of breastfeeding (improved nutrition, decreased risk of diarrhea, increased birth spacing, enhanced passive immunity from mother, etc.) need to be studied in parts of the world such as sub-Saharan Africa. We propose an innovative perinatal prevention strategy using highly active antiretroviral therapy (HAART) to maximally suppress maternal viral loading the late antenatal period and during the first six months of lactation in order to reduce the risk of HIV transmission via breast milk for HIV-infected women in resource-limited settings who opt to breast feed. Antiretrovirals (ARVs) would be given up to 6 months postpartum with weaning by that time. Neonates would also be given Nevirapine at birth according to the HIVNET 012 protocol. With this proposed protocol, infants would receive the benefits of breastfeeding while presumably being protected from the risk of HIV transmission antenatally, intrapartum, and postpartum during the first 6 months of breastfeeding, when the risk of transmission is highest. This study would also allow the assessment of safety and tolerance of these combination ARVs in this population. Weaning will be strongly encouraged at six months.

A HAART regimen of this nature may potentially show a dramatic decrease in transmission when compared to existing PMCT regimens. Given the continued decreasing costs of ARVs and the relatively low efficacy of existing PMCT regimens of around 30-50% in

breastfeeding populations, such a regimen would be seriously considered for wide implementation in resource poor settings and elsewhere.

Rationale for support of breastfeeding rather than formula

The proposed study area has a high infant mortality rate over 10% due to high rates of infectious disease. In keeping with the recommendations from WHO, UNICEF and UNAIDS, mentioned earlier, the Kenya government and the Provincial Medical Officer have continued to promote breastfeeding for HIV-infected women who have no access to clean, safe water. Alternatives to breastfeeding will be discussed per WHO guidelines and promoted in this study for those who can assure a safe supply of water, but formula will not be supplied based on Provincial Ministry of Health Policy [See accompanying letter, Appendix XII]. This is for several reasons; the supply of safe water in the study area is inadequate (Cholera outbreaks in this region are not uncommon); there is concern that offering formula will send a mixed message to HIV-negative women resulting in these women also choosing to formula feed; and the acceptance of formula feeding is low. A recent study in this area provided formula for HIV-infected women choosing not to breastfeed and ZDV for those choosing to breastfeed. (Personal communication: Elizabeth Juma; Medical Officer for JIKA study) Many of those who chose formula took it initially but breastfed anyway and did not return for more formula. Infants of these women therefore did not receive the added protection of ARV; moreover, may have been at increased risk of HIV infection due to mixed feeding. In two UNICEF supported pilot sites in Kenya (one of which is also in Nyanza province, and covers the same ethnic group as the study area) , formula is available at no cost to the women, but the acceptance of formula among HIV positive women who are aware of their status is 30% at best. In contrast, acceptance of ARVs is 70-90%. Reasons for this are complex but are thought to include: the value placed on breastfeeding; fear of being identified as HIV-

positive; stigma; social and cultural considerations; denial of HIV status; and water and sanitation issues. In the context of a culture and environment where formula is generally unacceptable as a widespread intervention, it is critical to test strategies which can reduce the risk of transmission for HIV infected women who choose to breastfeed in resource poor settings.

There has been growing concern about diarrhea among KiBS infants. A recent analysis compared rates of gastroenteritis of HIV-negative infants in KiBS with HIV-negative infants of HIV-positive mothers following traditional feeding practices in an HIV vertical transmission (VT) study conducted from 1996-2001 in Kisumu before PMTCT programs were established. Overall diarrhea incidence in KiBS was significantly lower at 6.1/100 infant-months of observation versus 8.9 in VT [RR = 0.70 (0.57-0.87)]. However KiBS had higher rates of diarrhea during the weaning period, ages 6-7 months [RR=1.62 (1.12-2.34)]. Higher rates of hospitalization of KiBS infants for gastroenteritis have also been observed. (Unpublished). In light of the high rates of diarrhea around weaning introduction of the following interventions are proposed:

1. Structured and repeated discussion with mothers on food and water hygiene to be done as group and/or individual sessions and to start during antepartum visits and continue through duration of the study (See Appendix XV)
2. Women will be offered a method to purify water; either a locally produced sodium hypochlorite solution, i.e. WaterGuard (dilute bleach) and/or a combination purification/flocculant product called Pur for those women who have turbid water.
3. Women will be offered a safe water vessel (a modified clay pot) with a narrow mouth and lid which does not allow people to contaminate the water by dipping a cup into the water container

Rationale for one-arm study

This is a one-arm study for several reasons. We believe there is not equipoise prenatally regarding reducing the risk of perinatal transmission by offering single dose NVP or short course prenatal AZT (both regimens being in wide use at the time the study was initiated and still in use) as a randomized comparison arm when compared to use of maternal HAART. In addition, given the rapid upscaling of HIV/AIDS treatment, it will be important to rapidly gather data from a phase II trial which can assess the safety, tolerance and adherence of a HAART regimen among HIV infected breast feeding women in resource limited settings; as well as to gather data on HIV transmission rates through 18 months postpartum. Safety, and tolerance data gleaned from the phase II trial will help inform the design and monitoring for phase III studies that will be comparing a peri-HAART regimen to the current WHO recommended ZDV plus SD NVP or to infant antiretroviral prophylaxis. Historical comparison of transmission rates will be made with data from previous studies (HIVNET O12 in Uganda and the placental malaria and HIV vertical transmission study in Kisumu) and with data from current Prevention of Mother to Child Transmission (PMCT) programs in Kisumu that are based on a single dose NVP to mother and infant. The vertical transmission study was conducted in the same population of women as the KiBS study. The PMCT programs are also providing services to the same population.

Choice of Antiretrovirals (ARVs): Characteristics and Rationale for Use of ZDV/3TC as Part of HAART During the Lactation Period

The two widely used nucleoside reverse transcriptase inhibitors, ZDV and 3TC have proven safe and highly effective as part of combination treatment regimens for HIV-1 infected adults. The combination has a relatively wide therapeutic window and good patient tolerance with few serious complications. The most frequent side effects are nausea, headache, myalgias and insomnia. The most common serious adverse events are anemia and abnormal liver function. Nucleoside analogue drugs are know to induce

mitochondrial dysfunction that can result in a variety of clinical disorders including hepatic steatosis and lactic acidosis. Although ZDV and 3TC are not as likely as some other NRTIs to induce mitochondrial toxicity, pregnancy may predispose women to this toxicity.[25] The safety profile has been quite good based on experience with tens of thousands of adult patients including several thousand pregnant women followed in U.S., European, and African cohorts. No congenital malformations or malignancies have been found in follow-up of children perinatally exposed to ZDV in the PACTG 076 trial.[25]

The half-life of 3TC is approximately 7 hours with relatively modest intersubject variability in patients with normal renal function. A small study of 20 subjects examined the pharmacokinetics of 3TC in HIV-infected pregnant women given the recommended dose of 150 mg twice daily in combination with ZDV as well as a higher dose of 300 mg twice daily given as monotherapy. Clearance, area under the curve (AUC), and half-life in HIV-infected pregnant women at both doses were similar to previous studies in non-pregnant adults, and no unexpected adverse effects occurred. These data support using the same dose and schedule for HIV-infected pregnant women that has been shown to be safe and effective for infected non-pregnant adults.

Transmission Rates with ZDV and 3TC

With respect to perinatal prophylaxis, a number of recent studies suggest that the use of combination ZDV/3TC during the latter part of pregnancy results in transmission rates as low as 1-3% among non-breastfeeding HIV-infected pregnant women compared with historical rates of 5-8% for women receiving ZDV only. A French study by Mandelbrot[26] found that adding 3TC to ZDV at 32 weeks resulted in transmission rates of 1.5%. Likewise recent data[27] from PACTG 316 (a randomized trial which looked at efficacy of single dose NVP to the mother and neonate in the background of prenatal ARVs to the mother and ZDV to the neonate and PACTG 367 (an observational study of pregnant women enrolled in PACTG trials looking at ARVs received and infant outcome) reported similar rates of HIV-transmission to the

infant [personal communication, R. Tuomala] In breastfeeding populations, the PETRA data[13] for Arm A (late antenatal, intrapartum, and 1 week postpartum ZDV/3TC) indicated an early transmission rate of 5.9% at 6 weeks; or about twice the early transmission rates noted with Zidovudine/3TC administered in non breastfeeding populations.

The rationale for adding a 3rd drug for transmission prophylaxis is based on possible further reduction in maternal viral load at delivery relative to the use of only two drugs. There would also potentially be a reduction in the risk for the emergence of drug-resistant virus. Initially NVP was chosen as the third drug for prevention of HIV transmission among breast feeding women based on available data on safety at that time, and that it was one of the first line regimens for treatment recommended by WHO for treatment in resource limited settings.

New Safety concerns regarding NVP in January 2005 lead to Amendment to KiBS study.

In January 2005 there was a revision to the NVP drug label warning[28] and a related FDA advisory[29] that indicated heightened early safety concerns with use of NVP among women with higher CD4 counts. On January 11, 2005 Boerhinger Ingelheim revised its drug warning label to highlight the increased risk hepatic-cutaneous sensitivity reaction for women >250/mm^3 CD4 count. The FDA released a related advisory on January 19, 2005 to reflect this increased risk and both the revised label and FDA advisory state that NVP should only be started among women with CD4 counts >250/mm^3 where the benefits clearly outweigh the risks. The FDA Advisory and revised Boehringer Ingelheim label also emphasized that the hepatic cutaneous hypersensitivity adverse events were most likely to occur within the first 18 weeks after NVP drug initiation, particularly the first 6 weeks. In follow up phone communication BI also indicated that after the first 6 weeks, the risk were substantially greater between 7-12 weeks than between 13-18 weeks (personal communication, Marita McDonough, BI). In reviewing the relevant KiBS safety data, four of the 22 SAE's occurred after 7 weeks. The rate of NVP toxicity requiring

stopping of NVP drug after12 weeks on was 3/ 241 or 1.2% and after 7 weeks was 4/214 or 1.7%. This compares to hepatic lab toxicity for NLF of 1% based on data reported from 2 trials.

In response to the revised drug label and the FDA advisory, the Division of HIV/AIDS Prevention, NCHSTP, CDC temporarily halted new enrollment into KiBS on January 21, 2005. Both CDC and KEMRI IRBs were notified. A consultation was convened on January 25, 2005 to discuss the implications of the revised label and FDA advisory for the KiBS study. Based on recommendations from that consultation and further discussions between Division staff and the Kenya KiBS team, the KiBS protocol was urgently amended to respond to the January 2005 revised label and the related FDA advisory . The key points of the KiBS amendment are as follows: 1) newly enrolled women with CD4 counts of $250/mm^3$ or higher at screening, will be offered a HAART regimen of ZDV/3TC and Nelfinavir; 2) newly enrolled women with CD4 counts $<250/mm^3$ at screening will be offered ZDV/3TC and Nevirapine; 3) women already enrolled into KiBS who are more than 6 weeks on NVP and who are tolerating the drug well based on clinical and laboratory findings will continue on NVP but with close monitoring already in place in KiBS. Women on NVP will be shifted off NVP to NLF for any clinical hepatitis, grade 2 or higher asymptomatic ALT elevation, grade 2B rash or other serious adverse event attributed to NVP. At this point in the study, there are no enrolled women on NVP-HAART .who have been on for less than 6 weeks of study drug, the highest risk period for hepatic-rash hypersensitivity reactions.

Safety concerns regarding Roche-manufactured Nelfinavir in June 2007

On the evening of June 5, 2007, the European Medicine Agency (EMEA) was notified by Roche Pharmaceuticals (official title Roche Registration Limited) that they had detected a potentially genotoxic compound (ethyl mesylate, EMS) in some batches of Nelfinavir. In animal studies EMS has been found to be teratogenic, mutagenic, and carcinogenic, but no data from humans exists. However, as such EMS is

considered a Class 2B carcinogen. As the extent of the contamination of nelfinavir was not known, the EMEA ordered an immediate recall of all stocks of Nelfinavir from the European Union market. As Roche manufactures Nelfinavir for widespread use in all global markets except for the United States, Canada, and Japan, Roche took steps to recall all Nelfinavir worldwide. The company notified the World Health Organization of their intent to recall global stocks of Nelfinavir on June 8, 2007. The WHO issued a statement describing this recall on June 11, 2007. As the potential long-term adverse effects of this potentially genotoxic medication on breastfeeding mothers and their infants are not known, steps were put in place to attempt to obtain Nelfinavir manufacture by Pfizer, marketed in the United States and not affected by the recall, specifically for KiBS participants who were on Nelfinavir at the time of the recall.

As of June 12, 2007 21 KiBS participants were on NLF: 14 had yet to complete the intervention for PMTCT (i.e. have not reached 6 months postpartum) and 7 were on NLF as part of their HIV treatment regimen. Through an existing mechanism the CDC-Kisumu site obtained enough NLF manufactured by Pfizer Pharmaceuticals for the 14 participants who were scheduled to complete NLF by August 2007. This supply was shipped to Kenya and arrived June 16, 2007. All participants were switched from Roche manufactured NLF to Pfizer manufactured NLF between June 19 and June 20, 2007.

As noted above seven of the participants needed antiretrovirals long-term for advanced HIV infection, but were unable to take nevirapine due to adverse effects with this medication previously. As NLF is not widely available within Kenya, we elected to initiate another protease inhibitor, Lopinavir/ritonavir (LPV/r or Kaletra®) used in the United States, Kenya, and multiple other locations worldwide for these 7 participants only. LPV/r is currently the first line protease inhibitor recommended by the Kenyan Ministry of Health. By switching these participants who needed long-term antiretrovirals to LPV/r-based regimens, this would make the transition for these participants to Kenyan HIV Care-and-Treatment programs easier. By June 21, 2007 all seven participants had been switched to LPV/r.

During the course of their investigation, Roche has noted EMS levels in some lots of NLF manufactured between March and June 2007 were more than 1000 times the maximum concentrations allowed by EMEA (0.6 – 1.0 ppm allowed). These lots were distributed to the following countries: Botswana, Burkina-Faso, Cameroon, Egypt, France, Germany, Iran, Italy, Kenya, Mali, Mexico, Mozambique, Nigeria, Portugal, South Africa, Spain, Taiwan, Uganda, Ukraine, and the United Kingdom. While some KiBS participants received Roche-manufactured NLF during the months of March and June 2007, the levels of EMS were one log concentration lower than that noted above (maximum EMS concentration among NLF taken by KiBS participants – 117 ppm). Using specific data on EMS levels for each NLF lot used during the course of the KiBS, we are now able to provide information to all exposed participants on their extent of exposure. Although the effects of short- or long-term exposure to elevated EMS concentrations in humans remain unknown, this information may help to gauge potential risk and help direct the extent of long-term follow-up of participants. All KiBS participants (including those who have completed the study) will be contacted and provided information about the recall if they received EMS contaminated NLF during any period of their participation in KiBS. An information sheet (See "Information to Participants in the Kisumu Breastfeeding Study: Why Nelfinavir [Viracept] was Recalled") explaining many of the issues regarding this recall and their exposure to EMS will be given to all participants, after which they will be given an opportunity to ask any questions they may have.

Roche continues to discuss plans for long-term monitoring of patients exposed to EMS-contaminated NLF in resource-limited settings. Thus far Roche has proposed two patient registries for long-term monitoring of any malignancies potentially associated with EMS exposure:

- One registry for patients who were exposed to NLF made from highly contaminated batches distributed from March to June 2007. This includes the twenty countries listed above (Botswana, Burkina-Faso, Cameroon, etc.)

- The other registry would include women who took the medicine during pregnancy and children who have taken NLF at any time or were exposed to it in the womb. This registry will include all patients receiving NLF since 1999 when NLF was first put on the market in the European Union.

Although the logistics of long-term follow-up of patients in resource-limited settings remain uncertain, Roche has proposed all patients included in the registries will be followed up every six months for a minimum of five years. The patients will be seen by their doctors, who will carry out a health status check. Once Roche finalizes their plans for ensuring long-term monitoring of all patients potentially exposed, the KiBS team will ensure all participants are included in these registries. However, as participants continue to complete 24 months of follow-up post-partum, participants will be asked to consent to allow KiBS staff to re-contact them once additional information becomes available. (See "Consent for continued follow up after completing participation in the Kisumu Breastfeeding Study")

Additionally, in response to the Roche-manufactured recall, the U.S. Food and Drug Administration requested Pfizer to assess EMS levels in Pfizer-manufactured Nelfinavir. Substantially lower levels of EMS (although still present) were noted in Pfizer-manufactured Nelfinavir. The U.S. Department of Health and Human Services (DHHS) issued a statement as of September 11, 2007 that the benefit-risk ratio of continuing Nelfinavir for the treatment of HIV in pediatric and adult patients remains favorable. For pregnant women the DHHS Panel recommended switching any patients on Nelfinavir to alternate regimens. Of note, all KiBS participants who received Pfizer-manufactured Nelfinavir were at least 4-5 months post-partum. At this stage it is not known how much EMS was present in the Pfizer batch of NLF which these KiBS participants received. We will update this section as we have more information.

Rationale for using NVP dosing as the third drug in Perinatal Prophylaxis

Nevirapine's potent antiretroviral properties[14] and ease of administration make it an excellent candidate for use with ZDV/3TC. HIV-infected pregnant women on this regimen would need to take only two tablets b.i.d. compared to 4-6 tablets b.i.d. if protease inhibitors were used. NVP is rapidly absorbed and decreases viral loadwithin a few days of administration. The drug is rapidly transferred across the placenta to the infant *in utero* when given during labor and delivery. Additionally, its long half-life permits simple dosing making it more feasible and affordable to implement in developing countries.

Nevirapine Characteristics

Nevirapine is a non-nucleoside reverse transcriptase inhibitor that binds directly to reverse transcriptase and blocks the RNA-dependent and DNA-dependent DNA polymerase activities by causing a disruption of the enzyme's catalytic site. NVP is widely distributed in the body. It readily crosses the placenta and is found in breast milk. Bioavailability following oral administration of NVP is excellent.[30]

Cytochrome P450 and glucuronide conjugation are the primary routes of NVP biotransformation. NVP has been shown to be an inducer of hepatic cytochrome P450 metabolic enzymes. The autoinduction is characterized by an approximately 1.5- to 2-fold increase in the apparent clearance of NVP as treatment continues from a single dose to two to four weeks of dosing with 200 - 400 mg/day (Manufacturer's package insert). [28]

No observable teratogenicity was detected in reproductive studies performed in pregnant rats and rabbits. In rats, a significant decrease in fetal body weight occurred at doses providing systemic exposure approximately 50% higher, based on AUC, than that seen at the recommended human clinical dose. In reproductive toxicology studies, evidence of impaired fertility was seen in female rats at doses providing systemic exposure, approximately equivalent to that provided with the recommended clinical dose of NVP (Manufacturer's package insert). [28]

Nevirapine for Treatment of HIV infection in Adults and Children

NVP in combination with other anti-retrovirals is used for treatment of HIV-infected adults and children in the U.S. and Europe. There is also some limited use in resource poor settings in Africa under the UNAIDS treatment pilot studies. The drug is well tolerated with rapid increases in CD4 cell count and reduction in viral load. Use of NVP monotherapy has been associated with rapid emergence of resistance by 6 weeks and viral levels returning to or exceeding baseline by as early as 4 weeks after starting therapy.[31] However, when NVP is given in combination with other antiretroviral drugs, striking reductions in viral loads are observed and maintained. NVP has been used in a number of pediatric clinical trials with data available on chronic administration including infants as young as 2 months.

Toxicity associated with Nevirapine

The most common mild to moderate clinical adverse effects related to NVP therapy are rash, fever, nausea and headache. The most significant clinical toxicities of NVP are rash and elevation of hepatic transaminases. NVP-attributable rash occurs in 17% of patients in combination regimens in Phase II/III controlled studies. Most rashes are mild to moderate maculopapular eruptions with or without pruritus located on the trunk, face, and extremities. Severe or life-threatening rash occurred in 7.6% of NVP treated patients compared to 1.2% of patients treated in control groups. The majority of severe rashes occurred within the first 28 days of treatment. (NVP package insert[28]) The rate of Stevens-Johnson syndrome is estimated at 0.3% based on data from 2861 adult patients receiving chronic NVP. Rash severity may be diminished by a two-week lead-in period with a lower starting dose.[32]

NVP chronic treatment can be associated with significant hepatic toxicity. In controlled trials, symptomatic hepatic events irrespective of grade occurred in 4% in patients on NVP versus 1.2% of patients in control groups. Asymptomatic elevations of GGT levels are more frequent in NVP recipients than in controls. Cases of severe, life-threatening hepatotoxicity and fatal fulminant hepatitis have been reported in patients, including pregnant women, on chronic NVP treatment; this toxicity is more common

during the first 18 weeks of therapy; and particularly the first 6 weeks. The early symptomatic hepatic-cutaneous hypersensitivity reaction has been noted to occur more frequently among women with >250/mm^3 CD4 counts and men >400/mm^3 CD4 counts reaction. In a retrospective review by Boehringer Ingelheim, "women with CD4 counts >250 cells/mm3 had twelve 12 fold higher risk of symptomatic hepatic adverse events compared to those with CD4 counts <250/mm^3. (11.0% versus 0.9%)."[28] It is not known whether the risk of hypersensitivity hepatic cutaneous reactions is the same among pregnant compared to non-pregnant women.

Co-infection with other infectious agents such as hepatitis B, C or cytomegalovirus increases the risk of liver toxicity. In clinical trials, NVP-associated clinical hepatitis has been reported in 1% of patients.[32] Careful monitoring of liver transaminases is essential, and NVP treatment should be interrupted in patients experiencing moderate or severe liver function test abnormalities (Manufacturer's package insert).[28]

The most frequently reported adverse events related to NVP in pediatric patients were similar to those observed in adults, with the exception of granulocytopenia, which was more commonly observed in children.[32,33] Serious adverse events were assessed in ACTG 245, in which pediatric patients received combination treatment with NVP. In this trial 2 out of 432 patients were reported to have experienced Stevens-Johnson toxic epidermal necrolysis transition syndrome.[34] Cases of allergic reactions, including one case of anaphylaxis, were also reported.

Nevirapine Use in Perinatal Trials

Nevirapine's potent antiviral affect, rapid oral absorption, good bioavailability, long half-life and ability to cross the placenta make it an ideal candidate for a single dose antiretroviral intervention during labor. The clinical trials using NVP during pregnancy have initiated dosing during the intrapartum period. More limited observational experience is available for use of NVP during pregnancy in combination with other

antiretrovirals.[35] Potential maternal toxicity associated with NVP use initiated during the last trimester of pregnancy, continued during the intrapartum period, and for 6 months postpartum would include rash, hepatic abnormalities, and hematologic adverse reactions. Safety data as of December 31, 2004 from KiBS was recently reported[36]. Of the first 241 women enrolled, 7.9% had an adverse event requiring cessation of NVP, with 3.7% due to hepatotoxicity, 2.9% due to rash and 1.2% due to rash/hepatotoxicity. Palombi et al[37] reported on safety and effectiveness of ZDV/3TC/NVP among 515 women during the last month of pregnancy and up to 6 months post partum in the DREAM project. Safety findings were that about 6% had grade 3 or higher hepatic toxicity and 3% had rash. No life threatening cases were reported related to drug. About 30% of women breast fed and the rest used formula. The transmission rate reported with this regimen was 4.1% at one month and 6.1% at 6 months.

Phanuphak et al[38] reported on the safety of ZDV/3TC/NVP among 157 HIV infected pregnant Thai women; and 93 nonpregnant women in the PMTCT plus program. Women with CD4 counts <200/mm^3 received the NVP- HAART regimen from 14 weeks gestation and continued post delivery. Those with CD4 counts >200/mm^3 received the regimen from 28 weeks gestation and stopped at delivery. Overall, 9.4% of women in PMTCT and PMTCT plus programs had either rash or liver enzyme elevation. 4/157 (2.5%) of pregnant women had grade 3-4 hepatitis and 6/157 (3.8%) had any grade hepatitis, Grade 3-4 rash was seen in 2/157 (1.3%) of pregnant women; and any rash was seen in 7% of pregnant woman. Grade 1-2 rash was more common in women >200/mm^3 CD4 counts (6/76) versus 0/81 among women <200/mm^3 CD4.

The Pediatric ACTG Protocol 250 team evaluated the safety, toxicity, and pharmacokinetics of intrapartum and early newborn NVP in 17 HIV-1-infected women in labor and their newborns.[30] No adverse effects of Nevirapine were noted in any study mothers or infants.

In the HIVNET 006 protocol, a phase I/II study evaluated the safety and pharmacokinetics of NVP in HIV-infected pregnant women and their infants in Uganda.[39] NVP 200 mg was given as a single oral dose during labor to 21 HIV-infected pregnant women, and13 infants received a single 2 mg/kg oral dose at 72 hours of age Nevirapine was well tolerated by women and infants, and no serious adverse events related to NVP occurred.

In the follow up phase II HIVNET 012 study in Uganda, Guay and colleagues determined that the two-dose NVP regimen decreased the risk of perinatal HIV transmission by 42% at 6 weeks and 41% at 18 months, with respective transmission rates of 11.8% at 6 weeks and of 15.7% at 18 months.[14,15] Similarly, the SAINT study in South Africa, which compared intrapartum and 1 week postpartum ZDV/3TC to single dose intrapartum NVP given to the mother and to the neonate, found similar 6-week transmission rates of 9.3% and 12.3 % respectively. In the U.S., where there is widespread use of maternal combination antiretrovirals during pregnancy, the PACTG 316 reported extremely low transmission rates of 1.5%. They found no difference in transmission risk for mothers and neonates receiving single dose NVP in the treatment arm (transmission rate 1.5%) and those in the NVP placebo arm.

Information reported by Stek *et al.* [35] at the 2001 Global Strategies Meeting in Kampala on the use of antenatal NVP in combination with ZDV/3TC among 99 U.S. women showed 79% of the women had RNA levels of < 400 copies/ml at delivery. NVP-attributed toxicities included 7 events of rash and 4 events of elevated liver enzymes and hepatitis. Of the 4 women with only a rash, 3 continued taking NVP with subsequent resolution of the rash; NVP was stopped in the fourth woman. Two women had both rash and hepatitis with maximal AST levels of 302 and 201; treatment was stopped in one woman, and interrupted and then resumed without complication in the other. Two women had liver AST elevation (to 306 and 322) only; both of which resolved after treatment interruption. All infants received 6 weeks of ZDV without evidence of complications. No HIV infections occurred among the study infants.

Emergence and Fading of Resistance with Intrapartum/Neonatal ARVs

Emergence of NVP resistance can occur after a singe dose of NVP and is induced by a single point mutation. This is thought to be due to unmasking of background quasi species having K103N or Y181C mutations when wild virus replication of NVP-sensitive virus is sharply inhibited.[40] In the HIVNET 012 study, emergence of the K103N mutation was seen at 6 weeks postpartum in 19% of 111 women evaluated in a sub study. By 12 months, there was reversion to wild-type of this mutation in 5/6 women for whom subsequent samples were available. In the HIVNET 012 study[41], risk factors for maternal development of NVP-resistant virus included longer half-life and slower clearance of NVP, lower CD4 counts, and increased maternal RNA levels. Of the 24 infected infants in the NVP treatment group, 11 (46%) demonstrated resistant mutations at 6-8 weeks, of which the majority were Y181C. At the 14-16 week follow up, 4 of 9 infants with available samples no longer had detectable levels of mutant virus; none of the 3 infants followed out to 12 months had evidence detectable mutant virus. Mortality rates were similar for HIV-infected infants with and without NVP resistant virus.

In the PACTG 316 study[42], where most women received combination therapy during pregnancy, a sub study of 89 women with replicating detectable virus noted NVP resistant mutations at 6 weeks postpartum among 13% (12/89) despite receipt of combination antiretrovirals antenatally. Resistant mutations to 3TC were noted among >70% women at 6 weeks postpartum.

In Thailand, short course prenatal ZDV for four weeks followed by single dose NVP at delivery to mothers was associated with a 15% rate of detection of maternal NVP resistant mutation at 6 weeks post partum.[43] In another Thai trial that included Zidovudine (ZDV) from 28 weeks through delivery plus single dose NVP to the mother and newborn and 1 week of infant ZDV, Jourdain reported K103N resistance to NVP was 20% at 6 weeks post partum.[44]

In another DITRAME Plus trial reported in 2005 from W. Africa,[45] women who received Combivir from 32 weeks through 3 days post delivery; single dose NVP at delivery had a transmission rate of 4.7 % and a NVP resistance rate of 1% at 6 weeks post partum.

Because the half life of NVP is much longer than that of other ARVs there is concern that discontinuation of NVP HAART all at one time may encourage development of resistant virus as the NVP will persist as a single drug for several days to weeks. To counter this concern, continued use of Combivir or other drugs for 1-2 weeks has been attempted.
Implications for future treatment options based on development of transient resistance to either single dose NVP or to short course 3TC are not known at the present time but are under study by CDC and other groups.

Resistance with Chronic NVP Treatment

Emergence of high-level NVP resistant mutants, usually Y181C and K103N, can be detected as early as 2 weeks after initiation of therapy among children who received NVP as a single drug regimen.[33] Among adults on chronic treatment, resistance was similarly noted within several weeks when administered in combination with ZDV.

Nelfinavir Characteristics[46]

Nelfinavir mesylate (NLF) is an HIV-1 protease inhibitor. It acts by inhibiting viral protease which prevents cleavage of the gag and gag-pol pro-proteins and leads to immature nonfunctional virion production. NLF is metabolized primarily in the liver by multiple cytochrome P-450 enzymes. Half life averaged 3.5-5 hours in adult volunteers. The adult dosage is 1250mg BID; or 750mg TID; and no significant pharmacokinetic gender differences have been found.

No observable teratogenicity has been observed with Nelfinavir and it is FDA category B. NLF has been used during pregnancy in the U.S. with a benign safety profile and generally mild gastrointestinal side effects. No evidence of a tumorigenic effect has been seen in mice with systemic exposures up to 9 fold of dosages used for human therapeutics. Thyroid follicular cell adeomas and carcinomas were increased in rats at doses that were 1-3 fold higher systemic exposures than in humans. There was no effect of NLF on fertility or embryo survival in rats at systemic exposures comparable to dosing equivalent to that used in human treatment

NLF has no significant drug interactions with didanosine, lamivudine, stavudine, zidovudine, efavirenz, nevirapine, or ketoconazole; or with trimethorim/sulfamethoxazole.. It should not be co-administered with antiarrhymics amiodarone, or quinidine; rifampin, ergotamine, St. John's wort, lovastatin, midazolam, triazolam, or pimozide.

Nelfinavir for Treatment of HIV infection in Adults and Children[46]

Nelfinavir in combination with other antiretrovirals has been used for the treatment of HIV infected adults and children. In protocol 511, among a randomized trial of 297 adults, use of NLF in combination with ZDV and 3TC was associated with 2/3 of patients achieving a viral load reduction <400 copies/ml at 48 weeks; and in a phase II AVANTI 3, 54% of patients had HIV viral load levels of <50 RNA copies/ml at 1 year or less.

In adult studies of >5000 patients, the most common clinical adverse effects were mild to moderate in intensity with diarrhea being most frequent. In two Agouron trials 511 and 542 involving 852 participants the most common moderate or severe adverse effects included; gastrointestinal adverse effects of diarrhea, nausea and flatulence with diarrhea were reported in 20% of adult patients receiving NLF 1250mg BID; rash was reported in 2% of patients when NLF was used in conjunction with 2 nucleoside

reverse transcriptase inhibitors; lab ALT elevations were noted in 1% of patients on NLF 750mg TID plus ZDV/3TC; hemoglobin abnormalities were found in 3% of adult patients and neutropenia in 2% of patients on NLF, ZDV, 3TC. A similar profile was seen in 400 pediatric patients. Other adverse events occurring in less than 2% of patients receiving NLF in all phase II/III clinical trials reviewed for the drug label and possibly related or of unknown relationship to the drug included redistribution/accumulation of body fat, elevated liver enzymes, and hyper and hypoglycemia. In post marketing experience, QTc prolongation and torsades de points have rarely been reported as have hypersensitivity, metabolic acidosis, jaundice and hyperbilirubinemia.

The safety and effectiveness of NLF have been studied in two clinical trials among 322 children 2-13 years. In infants and toddlers < 2years of age, NLF safety has likewise been shown among 78 infants enrolled in two phase I studies, although the optimal dosing has not been established, and responses appear poorer than among older children and adults. This may be related to highly variable drug exposure and increased clearance.

NLF Use in Perinatal Trials

Since 1996-97, combination antiretroviral regimens with 2 nucleoside reverse transcriptase inhibitors and either a non nucleoside reverse transcriptase inhibitor or a protease inhibitor (PI) such as NLF have been increasingly used in the U.S. for both treatment of pregnant women and for perinatal HIV prevention. PACTG 353[47] was a Phase I study that assessed the safety and pharmacokinetics of Nelfinavir among pregnant women when used in combination with ZDV and 3TC; and begun between 14-34 weeks gestation. The trial consisted of two cohorts. Cohort 1 included 10 women and assessed the safety and pharmacokinetics of 750mg tid; and cohort 2 assessed the safety and pharmacokinetics of 1250mg given bid to 23 women through the prenatal period on study drug, at delivery and at 6 weeks post partum .

Nelfinavir was well tolerated and no women stopped drug due to toxicity. The 1250 mg dosing had a pharmacokinetic advantage with increased AUC, trough and peak concentrations compared to the 750mg TID dosing. As with other PIs, there was little transfer of NLF across the placenta. The median ratio of the NLF concentration in cord blood to plasma at delivery was only 0.05. Drug levels in breast milk were not assessed. Significant increases in CD4 were noted between starting drug and 6 weeks post partum (average increase 139 cells during time on study drug) . In addition maternal viral load decreased during time on study drug NLF with 70% of women's plasma being below 400 copies at delivery and 83% at 6 weeks post partum. .

NLF is one of the most commonly used protease inhibitors during pregnancy due to its established safety and low transmission rates. It was the most common background PI that mothers received who took part in the PACTG 316 efficacy trial, which was carried out from 1997-2000 in the U.S. Europe, Brazil and the Caribbean. In this large trial of close to 1200 women, about 40% received PIs and NLF was the most common PI used with a good safety and tolerance profile.

In PACTG 1022[48], NLF-HAART was to be compared to NVP-HAART in an efficacy trial. However that study was stopped prematurely due to excess toxicity in the NVP arm including one maternal death from fulminant liver failure that was thought to be related to NVP. An upcoming phase II randomized trial planned in the PACTG, (concept sheet 4057), will assess the virologic effects of 2 alternative PI based HAART regimens (NLF or lopinavir/ritonavir plus ZDV/3TC) for PMCTC among women who are receiving antiretroviral only for PMTCT. Total sample size planned will be 480 women (240 per arm).

Resistance with Chronic NLF Treatment

HIV-1 isolates with reduced susceptibility to NLF have been selected in vitro. Cross resistance to other protease inhibitors has been reported.

4. JUSTIFICATION

There is an urgent need to identify a safe, effective means of preventing mother to child HIV transmission during the breastfeeding period in developing countries where a large number of HIV infected women choose to breast feed, and where the baseline rates of infant mortality are high due to multiple factors including malaria, diarrhea, other infectious diseases, malnutrition, poor sanitation and public health. In these countries, breastfed infants have a lower risk of infant and under 5 year mortality than their non-breastfed counterparts. Effective interventions, also feasible in the developing world, to reduce perinatal HIV transmission have been identified, but the infant remains at risk for HIV infection throughout the breastfeeding period.

The continued risk of transmission during breastfeeding is emphasized in the follow up of the short course ZDV and ZDV/3TC trials out to 18 months with late (postnatal) HIV transmission rates of 16-22%. These findings are sobering and stand in stark contrast to the overall transmission rates of <5% in the U.S. and Europe among non-breastfeeding HIV-infected women who receive combination antiretrovirals during pregnancy. They highlight the need to find effective interventions to reduce late transmission during breastfeeding among HIV-infected women in resource-limited settings. A variety of strategies are currently planned including antiretroviral prophylaxis to infants, exclusive breastfeeding followed by early weaning, and use of active vaccines and passive immune therapy to protect infants during the breastfeeding period. The goal should be maximal reduction of HIV transmission to rates of 5% or less, which are currently being achieved in the U.S. and Europe.

This study proposes to maximally suppress maternal viral load from 34-36 weeks of gestation through 6 months of exclusive breastfeeding by providing HAART (either ZDV+3TC+NVP or ZDV+3TC+NLF).

Such viral suppression should translate to maximal reduction in HIV transmission to the infant or at least a 50% reduction in transmission expected at 6 weeks, 9 and 18 months of age compared to transmission rates of either the currently available single dose NVP regimen or to transmission rates for historical controls in the same setting. The proposed trial will specifically test the hypothesis that administration of HAART during the last 4-6 weeks of pregnancy and continued for up to 6 months postpartum during lactation followed by early weaning will substantially reduce maternal viral loadin plasma and breast milk. HIV transmission rates among the trial cohort will be compared to those of historical controls. In addition, the transmission rates in the study group will be compared to the rates among contemporaneous HIV-infected pregnant women who are not in the trial but who receive a single dose of NVP during labor and whose infants also receive a single dose of NVP within 72 hours of birth as part of the Kenya Ministry of Health/CDC Global AIDS Program (GAP) activities.

This unique and innovative approach to reduce transmission has not been assessed in trials among breast feeding HIV infected women in resource limited settings such as East Africa. This research proposal is timely because reductions in drug pricing and increased funding from external international groups have now put combination therapy within reach of a larger proportion of HIV-infected individuals in Africa. The project, if successful, will demonstrate that combination ARVs can be effectively and safely dispensed to HIV infected pregnant women and monitored in resource-limited African settings.

The study will also allow evaluation of ARV acceptability, adherence, toxicity and the development of viral resistance. Information will be obtained that may help determine minimal patient monitoring requirements for future interventional programs. In addition, the study can identify and characterize potential social and cultural ramifications for women taking ARVs. This information is crucial in developing programs for distributing and monitoring ARVs once they are more accessible nationwide. In short, this study has the potential to change international policy involving mother-to-child HIV transmission and to guide policy development for accessing and monitoring ARVs.

In the intervention, Combivir b.i.d. (ZDV 300mg/ 3TC 150 mg) and either 200mg NVP p.o. bid or NLF 1250mg p.o. bid will be given to HIV-infected pregnant women volunteers who have chosen to breastfeed after having been counseled on the risks and benefits of breastfeeding and other alternatives. Women with CD4 counts <250/mm^3 at entry will be given NVP/ZDV/3TC unless they test positive for hepatitis B or C infection in which case they will be started on NLF/ZDV/3TC; and those with CD4 counts >250/mm^3 at entry will be given NLF/ZDV/3TC. Use of NLF for those women with hepatitis infection is based on findings that those with hepatitis were at higher risk or hepatotoxicity if started on NVP. Use of NLF-HAART among women with CD4 counts >250/mm^3 in the trial was done in response to a January 19, 2005 FDA advisory indicating a higher risk of hepatic-cutaneous hypersensitivity reactions among women starting NVP who had initial CD4 counts >250/mm^3 ; and because the safety experience with NLF among pregnant women in the U.S. suggests a very benign safety profile for NLF. In addition, those women who were recently enrolled with CD4 counts >250/mm^3 and began NVP in the KiBS trial, and are still within the early highest risk period for this hypersensitivity reaction (first 6 weeks) with NVP, will be switched from NVP-HAART to NLF-HAART based on the January 2005 FDA advisory. Since the KiBS study is approximately halfway enrolled, this will afford the opportunity to assess and compare rates of toxicity of the NVP and NLF based HAART regimens. Such information will help inform programmatic activities regarding the type and intensity of laboratory monitoring required for the two HAART regimens.

Due to the worldwide recall of NLF by Roche all participants had been receiving NLF (either for PMTCT during breastfeeding or for their own health) at the time of the recall were switched to NLF manufactured by Pfizer Pharmaceuticals (at the same dose – 1250 mg po bid) or to LPV/r. As noted previously only 21 KiBS participants were on NLF at the time of the recall: 14 had yet to complete the intervention for PMTCT (i.e. have not reached 6 months postpartum) and 7 were on NLF as part of their HIV treatment regimen. The 14 participants who were taking NLF for PMTCT were switched from

Roche manufactured NLF to Pfizer manufactured NLF on June 19-20, 2007. The remaining seven participants on NLF for treatment of their HIV had been switched to LPV/r by June 21, 2007.

Women who agree to take part in this open label phase II study will be enrolled between 28-35 weeks gestation. ARVs will be initiated between 34-36 weeks gestation and then continued while breastfeeding up to 6 months with abrupt weaning at that time. Infants will be given one 2 mg/kg dose of NVP p.o. within 72 hours of birth. Both women and their infants will be followed for 24 months after delivery.

In accordance with the WHO/UNAIDS/UNICEF guidelines, HIV-infected mothers will be advised of the risks and benefits associated with breastfeeding at pre-entry as part of the Ministry of Health (MoH)/GAP/PMCT program at the New Nyanza Provincial Hospital, which has been ongoing since May 2001. This program has also started at Kisumu District Hospital also. Recent data from the PMCT program indicate that 98% of the HIV-infected women who deliver in the hospital chose to breastfeed. All HIV-infected women obtaining antenatal care will be approached regarding this research study. The women will be counseled on the risks and benefits of breastfeeding and breastfeeding alternatives. Options for prevention of transmission will be presented along with their strengths and weaknesses, these will include: 1) enrolling in the PMCT program where 2 options are currently being offered; either single dose of NVP for mother and neonate (reduces risk of transmission to the child by 48%), or receiving ZDV from 28 weeks, NVP to mother and infant at birth and then 1 week of ZDV to infant; or 2) enrolling in the study and receiving HAART regimen for 7 months which is expected to decrease HIV transmission even further but is more complicated and has inherent side effects; and/or 3) using formula (for which no assistance programs exist) or other form of breast milk substitute. Those who enroll in the study will be advised to stop breastfeeding as soon as they can switch to safe breast milk substitutes with nutritional counseling on appropriate nutrition and local breast milk substitutes/alternatives. Women will be strongly counseled and encouraged to wean over a several week period during the sixth month postpartum if they

have not weaned before that time. They will be informed of the potential for increased risk of HIV transmission to their infant if they continue to breastfeed after the ARVs have been stopped.

HAART will be stopped at 6 months or sooner if the woman has discontinued breastfeeding earlier. Mothers who wean at an earlier time point will be counseled on safe use and preparation of formula or other breast milk substitute. Their infants will be closely monitored for growth and nutrition associated with this feeding method. All infants in this study will be followed to monitor growth, and survival, and to detect late postpartum transmission. Efficacy in the reduction of HIV-transmission to the infant will be assessed at 3 primary time points—6 weeks, 9 months, and 18 months.

Women who meet the current UNAIDS criteria for combination antiretroviral treatment (either with CD4 count <200/mm^3, or with clinical symptoms of AIDS (WHO Stage 4 disease) as well as those with WHO Stage 3 disease and CD4 <350/mm^3) either at initiation or subsequently through the study period, will be continued on HAART study drugs for the time they are in the study. (These criteria will be amended to reflect any changes made by UNAIDS). After completion of their participation in the study, these women will be referred to services through the MoH/GAP HIV/AIDS Care and Treatment programs and/or PMCT-Plus. PMCT -Plus will provide ARVs for women and members of their family who meet criteria for treatment. Funding has been approved to offer this program through the Provincial Hospital. Kenya is also one of the countries that receives substantial funding through President Bush’s International Mother and Child HIV Prevention Initiative and the related Presidential Emergency Plan for AIDS Relief (PEPFAR). Part of this initiative includes provision of combination antiretroviral therapy to women who meet WHO criteria for treatment. As women complete the KiBS study, those who meet treatment criteria, will be referred for treatment to the MoH/GAP HIV/AIDS Care and Treatment programs. If at the time the trial is completed treatment programs are no longer available through PMCT-Plus or the PEPFAR/GAP Care program, the study clinic will continue to provide ARVs to women who met criteria during the study until referral treatment programs again become available.

5. HYPOTHESES

The proposed intervention of ZDV/3TC and NVP or NLF to the mother from the late antenatal period through 6 months of breastfeeding will result in the following:

1. No unexpected toxicities or safety issues for the mothers; rates of hepatic or hematologic or dermatologic toxicities will be no more than 50% higher than those reported in the U.S. on similar regimens
2. No serious adverse events or evidence of hepatic, hematologic or dermatologic toxicity for the infants exposed to low dose antiretrovirals through maternal breast milk
3. At least a 50% reduction in mother-to-child HIV-transmission compared to rates using the single dose NVP regimen in the PMCT implementation program at Provincial General Hospital (PGH) and Kisumu District Hospital (KDH) or the HIVNET 012 trial results or to historical controls from the pregnancy and malaria study at PGH
4. At least a 50% improvement in infant HIV-free survival, compared to rates using the single dose NVP regimen in the PMCT implementation program at the PGH and KDH or to historical controls from the pregnancy and malaria placental study at PGH
5. Maternal adherence to HAART will be positively correlated with disclosure to her partner.

6. OBJECTIVES

The overall aim of the trial is to demonstrate that a regimen using highly active antiretroviral therapy (HAART) to maximally suppress maternal viral load in the late antenatal period and during the first six months of lactation is safe, well tolerated and effective; and that the intervention can be practically

implemented among HIV-infected pregnant and lactating women in resource poor settings in order to reduce the risk of HIV transmission to the infant.

6.1 Primary Objectives

1. To estimate the cumulative risk of infant infection at 6 weeks, 9months, and 18 months of age among breast fed infants in an open label trial whose mother's breastfeed for up to 6 months and have been on the described ZDV/3TC and NVP or NLF regimen. The anticipated outcome is a transmission rate of <6% at 6 weeks and <8% at 18 months of age.

2. To determine infant HIV-free survival rates at 24 months of age.

3. To evaluate infant and maternal safety, and tolerance of a regimen of ZDV/3TC and Nevirapine or Nelfinavir given to HIV-infected pregnant women beginning at 34 weeks gestation and continuing for up to 6 months postpartum while exclusively breastfeeding.

6.2 Secondary Objectives

1. To determine infant survival regardless of HIV infection status at 24 months

2. To characterize changes in maternal CD4 counts and plasma viral load from the baseline at HAART initiation, delivery and then at 6 weeks, 14 weeks, 6, 9, 12, 18 and 24 months; and to characterize the relationship of maternal CD4 counts and viral load at these time points to the risk of HIV transmission to the infant.
3. Comparison of rates of serious adverse events requiring change of drug for Nevirapine compared to Nelfinavir among women with CD4 counts >250/mm^3 enrolled into the KIBS trial.

4. To identify and quantify the rates and types of antiretroviral resistant mutations of virus at delivery, 6 weeks, 14 weeks, 6, 9, 12, 18 and 24 months postpartum in maternal plasma and infant plasma.

5. To document maternal acceptance of and adherence to exclusive breastfeeding during the period of lactation.

6. To document maternal acceptance and adherence to taking ZDV/3TC and NVP or NLF in the third trimester and during the first 6 months of lactation.

7. To identify and characterize any negative and/or positive social consequences on women taking ARVs in this cultural context.

8. Breast milk sub study: To quantify the viral load, emergence and fading of resistant mutations, and drug levels in blood and breast milk collected at delivery, 2 weeks, 6 weeks, 14 weeks, 6, and 9 months among a subsample of women and their infants. The specimens are to be collected on 150 mothers and infants enrolled in the overall phase II study who agree to take part in this substudy and who early on in the study show that they are adherent to drugs and study schedule in order to give reliable, accurate self-report of the exact time of drug ingestion.

9. To assess infant growth during breastfeeding and after weaning as stratified by infant HIV-infection status.

10. To assess maternal nutritional status during the study. This will be assessed using height, weight and mid upper arm circumference measured within first week after delivery and then at 6 weeks, 14 weeks, 6, 9, 12, 18 and 24 months postpartum.

11. Maternal mortality rates at delivery, 6 and 14 weeks, 6, 9, 12, 18 and 24 months using hospital records and/or information from relatives.

7. DESIGN AND METHODOLOGY

This is a phase II, open-label, one-arm trial designed to assess the effectiveness, safety, and tolerability of highly active antiretroviral combination therapy (HAART) used to maximally suppress maternal viral load in the late antenatal period and during lactation in order to reduce mother-to-child transmission among breastfeeding HIV-infected women in a resource-limited setting.

Representatives from the Ministry of Health (i.e. Provincial Medical Officer, Provincial Pediatrician, Hospital Medical Superintendents, hospital and clinic staff, etc.) have actively provided and will continue to provide support and input into the study design, logistics, feasibility, cultural appropriateness, etc. of this study**.** Focus group discussions are being held to aid in the assessment of community and individual acceptance of antiretroviral treatment of HIV infected pregnant women and women postpartum; and acceptance of exclusive breastfeeding during the first 6 months of life with abrupt weaning at that time. Other issues being addressed will include infant feeding practices, attitudes toward participation in research, how best to ensure compliance, who is best to do follow-up, how to minimize loss to follow-up etc. (See Appendix X for Focus Group Discussion Protocol)

7. 1 Study site description

Kisumu is a city with an estimated population of 300,000 situated in western Kenya on the shores of Lake Victoria. It serves as the provincial headquarters for Nyanza Province and is the largest city in this part of

Kenya. Kisumu is served by two main government hospitals; the Provincial General Hospital (PGH) and the Kisumu District Hospital (KDH). CDC/KEMRI has established a Clinical Research Center at PGH. Studies investigating a variety of topics, including malaria and tuberculosis, have been conducted at this site.

Western Kenya has the highest prevalence of HIV in the country. The HIV-1 prevalence among pregnant women in Kisumu is estimated to be 25-36%, and HIV transmission from an HIV-infected mother to her infant is estimated to be 22% at 2 years. The supply of water in Kisumu is unreliable and not safe. (Outbreaks of cholera are not uncommon in this area).

As part of the PMCT program, CDC/GAP in collaboration with the Kenya Ministry of Health is providing 2 regimens of either short course ZDV/NVP or single dose Nevirapine as described earlier to HIV-infected pregnant women in Kisumu to decrease mother-to-child-HIV-transmission rates in infants.

7.2 Study population

As described in the background section, the HIV-1 prevalence among pregnant women in Kisumu is estimated to be 25-36%, and HIV transmission from an HIV-infected mother to her infant is estimated to be around 22% in a healthy, asymptomatic population. Extrapolating from these rates to all 2,200 deliveries at New Nyanza Provincial Hospital per year, at least 120 HIV-infected infants are born annually. Assuming similar rates of transmission for the estimated 1,200 deliveries at the Kisumu District Hospital per year, at least another 80 HIV-infected infants are born there annually. Similarly, these two hospitals would see an estimated combined total of at least 850 deliveries from HIV-infected women per year, assuming a 25% HIV prevalence rate. The actual prevalence and perinatal transmission rates may be higher, because the placental malaria study excluded all clinically ill women.

Over 60% of the women who deliver at PGH come from 3 neighborhoods in the slum areas of Kisumu (43% from Manyatta, 10% from Nyawita/Obunga, and 11% from Nyalenda). Most women from Nyalenda choose to deliver at KDH instead of PGH because of location and cost. Existing networks of community health volunteers, traditional birth attendants, supervisors, etc. will be strengthened and reinforced in these neighborhoods in which most of the study participants reside.

It is anticipated that 30-40 women would be enrolled in the study per month, leading to early 6-week outcome data in approximately 24 months and completion of data collection in approximately 48 months. Experience with previous prospective HIV intervention studies in other East African settings has shown that minimal loss to follow-up rates (10% per year) can be maintained with extensive home visiting and outpatient clinic services. Maximal effort will be made to minimize loss to follow-up, including identification and assignment of caseworkers, frequent communication and home visit support, and strengthening the existing health care delivery and reporting network in strategic neighborhoods.

Hospital staff will carry out voluntary counseling and testing services at PGH and KDH as part of the ongoing PMCT program. Most women (80%) present to the antenatal care clinic (ANC) by 32 weeks gestation for services. All ANC attendees receive group education about various medical issues. They are educated about HIV and the risks of transmission to the infant. They will be introduced to the options that are available to them if they test positive. Hospital staff will further educate mothers who are identified as HIV-infected about the risk of HIV transmission to their infant and the various options available to help minimize that risk. Women will be informed about the risks of breastfeeding but will be supported in whatever feeding option they choose. Women who have decided to breastfeed and meet eligibility criteria will be invited to participate in the study. After explanation of the study and obtaining informed consent, we will enroll all women who meet eligibility criteria into the study. In all cases, women will be advised, counseled on exclusive breastfeeding and strongly encouraged to discontinue breastfeeding at 6 months or

earlier unless the infant is found to be HIV-infected. The average duration of breastfeeding in Kenya is currently 17 months with over 95% discontinuing by two years.[49]

Eligibility

Participants will be recruited from the pregnant women presenting to the ANC clinics at PGH or KDH in Kisumu Kenya before 34 weeks gestation (see Appendix III for Enrollment Flow diagram). These women will have access to voluntary confidential counseling and HIV-1 antibody testing by trained nurses/counselors. Those who provide informed consent, agree to be tested, to receive their test results, to receive post-test counseling, and are found to be HIV-infected will be further screened for study eligibility by a nurse or counselor and invited to participate in the clinical trial. HIV-infected women who meet all other eligibility criteria including having already decided to breast-feed will be offered the opportunity to participate in the study at 26-34 weeks gestation. It is currently standard of care at PGH that HIV-infected women be able to receive the two dose NVP regimen for the prevention of HIV transmission to their offspring as part of the PMCT program. HIV-infected women will have the opportunity to choose between participating in the HAART trial or taking one of the regimens being promoted by the MoH at the 2 hospitals. If they agree to participate in the trial, women will have pre-entry evaluations between 26 and 34 weeks gestation to determine eligibility. Enrollment clinic physical examinations and laboratory assessments will then be repeated between 32-35 weeks to ensure continued eligibility prior to starting ARVs. Women who meet the inclusion eligibility criteria will be assigned a study-specific identification number. As part of consenting for the study, the enrolled will agree to allow a member of the study staff to accompany her from the clinic to her residence to document the location of her residence and gather directions to other locations where she may reside (including homes of extended family members). Recruitment, screening, and follow-up procedures will be detailed in the study manual of operations.

7.2.1 Study Inclusion Criteria-Women

To be eligible for inclusion in the study, women MUST at screening:

1. Be $\geq$18 years of age, or $\geq$15 and $\leq$18 years of age and able to assent as well as obtain consent of a parent or legal guardian

2. Be a pregnant HIV-infected female presenting prior to 34 weeks gestation who has already chosen to breastfeed after receiving counseling on infant feeding choices according to UNAIDS guidelines which includes counseling and education about the overall benefits of breast feeding as well as the risks of HIV transmission to the infant inherent in breastfeeding.

3. Report that they plan to reside in Kisumu for the next 2 years

4. Be able to give competent, informed consent if $\geq$18 or have a parent or guardian who can do the same in the case of a minor.

5. Be willing to comply with study requirements if they meet study eligibility criteria.

6. Meet the following laboratory criteria at enrollment (Efforts will be made to address potentially correctable abnormalities such as anemia, prior to enrollment)
    a. Documentation of HIV-1 infection according to the National PMCT testing algorithm. The policy for Voluntary Counseling and Testing in Kenya requires that no document carrying a person's HIV result be given out. In the event that a potential participant is referred to the study recruiter by the hospital PMCT nurse or counselor without any documentation, all effort will be made to obtain some indication of HIV status either

from the laboratory form, the record of the log book where results are supposed to be recorded or the HIV result section on the Antenatal Card. If none are available, recruitment will take place and a confirmation test will be done. (This is done for all participants)

b. Serum creatinine <1.5 mg/dl

c. Hgb ≥7.0 g/dL

d. Absolute neutrophil count > 1000 cells/ml

e. Platelet count >50,000/ml

f. SGPT < 2.5 times upper limit of normal

g. Documentation of CD4 count results prior to beginning study drug; which will be used to determine the appropriate HAART regimen –i.e ZDV/3TC/NVP or ZDV/3TC/NLF

h. Documentation of Hepatitis B and C infection status (Hepatitis B surface antigen and Hepatitis C antibody)

7. Have signed consent and met clinical and laboratory eligibility criteria in order to be enrolled in the trial by 34-36 weeks gestation (preferably at 34 weeks).

Once the woman has met eligibility criteria and given signed, informed consent (or assent with parental consent in the case of a minor) to take part in the study, she is considered to be enrolled in the trial.

7.2.2 Study Exclusion Criteria-Women

Patient volunteers will be excluded from participation in this study if they have any of the following characteristics:

1. Is participating in other HIV vaccine or antiretroviral trials.

2. Has substantial hypersensitivity to any benzodiazepine, including Nevirapine.

3. Has history of prior substantial intolerance or severe allergic reaction to Nevirapine, Zidovudine, Lamivudine or Nelfinavir.

4. For women who will be placed on NVP, ongoing treatment with rifampin, anticoagulants, benzodiazepines, and magnesium sulfate at time of planned enrollment. For those women who will be placed on NLF, ongoing treatment with amiodarone, quinidine, ergot derivative drugs, rifampin, pimozide, St John’s wort, lovastatin, simvastatin, midazolam or triazolam

5. Has evidence of clinically significant cardiac, respiratory, hepatic, gastrointestinal, endocrine, hematologic, psychiatric, neurologic, or allergic disease that would compromise the ability of the participant to complete the study or the study requirements as determined by the principal investigator or designated associate. The clinical significance of any abnormality is to be evaluated in the context of the safety of the patient volunteer and the objectives of this study.

6. Has a history of cytotoxic chemotherapy within one month prior to study entry or current diagnosis of malignancy for which systemic therapy is expected to be required during the period of study.

7. Blood pressure $\geq$ 160 mm Hg systolic or $\geq$ 110 mm Hg diastolic.

8. Chronic alcohol or illicit drug use.

9. Women who become pregnant again during the study follow-up will NOT be eligible for re-enrollment in the trial if they were enrolled for their previous pregnancy.

7.2.3 Infants

All infants born to HIV-infected women enrolled in this trial will be included and followed in the study. Infants born to HIV-infected women who were not enrolled will not be included in the trial. Infants may be excluded from receiving the neonatal dosing of NVP according to the criteria listed in section 8.62 but will continue to be followed with all clinical and laboratory evaluations as required to complete the study protocol.

7.3 Sampling

Sample size determination

Cumulative infection risks in the HIVNET 012 study in the NVP arm at 6 weeks, 9 months, and 18 months of age were approximately 12%, 14%, and 16%, respectively. Infant HIV-free survival at 18 months was 79%. We wish to be able to detect a 50% reduction in cumulative infection risk and in the cumulative risk of infection or death.

Based on previous experience, the variance from a Kaplan-Meier estimate for cumulative risk will be similar to the variance of a binomial proportion. Therefore, we calculate study power based on comparing binomial proportions (after an arcsine-square root transformation). There were 310 children evaluable for HIV-free survival in the NVP arm of HIVNET 012, of whom two had no HIV test. With a sample size of 400 evaluable mother-infant pairs in this study, a Type I error rate of 5%, and no loss to

follow-up, we could detect a 50% reduction (i.e. <6% transmission at 6 weeks and <8% at 18 months) in cumulative transmission risk with power 80%, 86%, and 91% at 6 weeks, 9 months, and 18 months, respectively. We can detect a 50% reduction in the cumulative risk of infection or death at 18 months with power 97%.

Loss to follow-up will reduce power somewhat. However, because we use the Kaplan-Meier procedure and because most infected children have a positive test by age 3 months, children lost to follow-up after 3 months contribute substantial information to our estimates. Therefore, with 10% loss to follow-up, conducting this study based on 480 children should give even better power than described above.

7.4 Procedures *(see Appendix III for Enrollment Flow diagram*)

All women attending the ANC clinic are offered voluntary counseling and HIV testing as per the routine standard of care offered at the clinic. As part of the voluntary counseling and testing program, a woman identified as HIV-infected are counseled regarding infant feeding options that might protect her unborn child from becoming infected. She will be counseled according to WHO guidelines regarding the risks of HIV transmission through breastfeeding, and her plans regarding type of infant feeding will be reviewed. Additionally, the woman will have an opportunity to have her questions regarding HIV. After the woman has indicated to the counselor that she will breastfeed, she will be referred to the KiBS nurse recruiter. An appointment will be made for her to see the KiBS nurse recruiter within the next week.

Those choosing to be considered for participation in the KiBS will go through pre-enrollment screening. If eligible at this point, the study will be explained and consent obtained if the woman agrees to participate. Once consent is obtained blood will be drawn to determine full eligibility. Eligibility must be determined before 35 weeks gestation. The laboratory results will then be reviewed to determine if the woman meets study criteria. Women who have abnormal results will be referred back to the hospital

antenatal care clinics for appropriate care. HIV-infected women who are fully eligible will be enrolled in the study. At enrollment, photographs of participants will be taken. These photographs will be used primarily to verify the participant's identity at clinic visits. However, they will also be used to track participants who become lost to follow-up. Blood will be drawn at this time for baseline laboratory assessment of HIV status, malaria, and to confirm normal renal and hepatic function. Open label study drugs ZDV/3TC and NVP or NLF will be initiated at about 34 weeks gestation (36 weeks gestation at the latest).

Breast milk pharmacokinetics substudy (See Appendix). Recruitment into this breast milk substudy will be offered consecutively to the women as they enroll in the main study until 150 women have been enrolled. A separate consent will be obtained for this substudy. MEMS caps will be used to measure adherence in this substudy. In addition 30 women in the breast milk study will be asked a brief (20 minute) questionnaire regarding the acceptability of the MEMS CAPS, and whether going over the visual print out of their pill taking based on MEMS helped them adhere to the regimen. Interviews will be taped unless consent for taping is not given, in which case notes of the interview will be taken. Women will be offered a token of appreciation for participating, either a small bag of rice or sugar. See appendix XIII for questionnaire on MEMS. Participants will be asked to bring their medication bottles to each study visit. At the end of the study, participants who return their bottles and MEMS caps will receive 1000 Kenyan shillings (14 USD) This is to provide them an incentive to complete the substudy and to return the Cap holding important data on time of opening and closure of the pill bottle. At several points during the study breast milk will be obtained from these women and stored. This will be used to:

1. Quantify viral load, emergence and fading of resistant mutations in breast milk and concurrent plasma samples weekly from delivery, 2 weeks, 6 weeks, 14 weeks, and 6 months.

2. Quantify drug levels in maternal breast milk and maternal and infant plasma using population pharmacokinetics techniques and electronic medication vials (MEMS) to document the timing of dosing in relation to blood draw as well as adherence.

3. Quantify the infant exposure to antiretrovirals through breast milk among women taking highly active antiretroviral therapy.

7.4.1 *Study Treatment – Drug Regimen*

The regimen for ZDV/3TC (Combivir) and NVP or NLF is as follows:

1. Antenatal (34-36 weeks to delivery)

   Women

   NVP will be given to all new enrollees with screening CD4 <250 . Study NVP will be started between 34-36 weeks gestation with a lead in dose for two weeks of 200mg QD (one pill daily), followed by 200mg bid (1 pill twice a day) . These women will also receive Combivir (300mg ZDV plus 150 mg 3TC) as one tablet bid po to start between 34-36 weeks gestation

   For all new enrollees with screening CD4 equal to or greater than 250, or those with CD4 <250, but testing positive for Hepatitis B surface antigen or Hepatitis C antibody, NLF 1250 mg bid will be given and Combivir (300mg ZDV plus 150 mg 3TC) as one tablet bid po to start between 34-36 weeks gestation.

2. Labor, delivery and Neonatal period:

   Women

NVP 200 mg p.o. bid; or NLF 1250 mg p.o. bid depending on maternal drug assignment

Combivir one tablet bid.

Infants - Single dose of 2 mg/kg NVP po at 72 hours of birth or just prior to discharge if discharge occurs before 72 hours of age. Mothers who deliver at home will be encouraged to bring their infants into the study center in the first week. The infant dose of NVP will be given up to 7 days after delivery.

3. Postnatal (for 6 months or through weaning if this occurs before 6 months)

Women-

NVP 200 mg p.o. bid or NLF 1250mg p.o. bid depending on maternal drug assignment

Combivir one tablet bid. For women on NVP, Combivir will be continued for 21 days after cessation of NVP in an attempt to reduce risk of emergence of NVP-resistant virus.

Infants—no postnatal prophylaxis given after the single neonatal dose of NVP

Treatment modification before the end of the study will take place for the following reasons:

Patient intolerance; deterioration of immune/clinical status (i.e. Declining CD4 count that remains below 200/mm^3 on two occasions or occurrence of AIDS defining condition); occurrence of serious adverse events felt to be related to the treatment. Reasons for treatment interruption or modification will be reported. The woman will be switched to another highly active regimen according to drug availability and the best clinical judgment of the study clinician.

7.4.2 Drug Dosage and Administration

Maternal ZDV/3TC and NVP or NLF

ZDV/3TC and NPV or NLF will be administered starting at 34 (no later than 36 weeks) gestation according to the regimens described above. Women will be instructed to take the Combivir and NVP or NLF pills about every 12 hours (bid). They will return to the clinic for routine antenatal care every week with study-related clinical and laboratory evaluations every 2 weeks until delivery. If they develop a generalized rash or any serious problems they think may be related to the drugs, they will be instructed to come to the clinic within the next 12-24 hours for evaluation and to discontinue therapy until they have been evaluated by a study clinician.

At onset of labor, women will be instructed to come to the PGH or KDH for delivery. They will be continued on study ARVs during their admission stay. They will be instructed on appropriate breast care to reduce the risk of mastitis and bleeding or cracked nipples. Women who deliver at another facility or at home will be instructed to come to the study clinic within the first week.

At discharge, women will be continued on Combivir and NVP bid or NLF bid for up to 6 months or until weaning, if this occurs sooner. At 6 months, a woman may be continued on drugs for a maximum of 2 weeks if weaning off the breast has not been completed or the baby is sick and unable to tolerate replacement food. Asymptomatic women with CD4 >200 will then be instructed to abruptly stop treatment.

Women who meet the current WHO/UNAIDS criteria for combination antiretroviral treatment (either with CD4 count <200/mm^3, or with clinical symptoms of AIDS [WHO Stage 4 disease] as well as those with WHO Stage 3 disease and CD4 <350/mm^3) either at initiation or subsequently through the study period, will be continued on study ARVs for the time they are in the study. (These criteria will be amended to reflect any changes made by WHO/UNAIDS). After completion of their participation in the study, these women will be referred to services through the recently funded PMCT-Plus program. This

program will provide ARVs for women and members of their family who meet criteria for treatment. Funding has been approved to offer this program through the Provincial Hospital. Kenya is also one of the countries that is to obtain funding through the Presidential Emergency Plan for AIDS Relief, (PEPFAR). One arm of this initiative will be to provide combination antiretroviral therapy to pregnant women. Provision of ARVs in Kisumu is currently available through the Kisumu GAP Care program. In the unlikely event that local treatment programs are no longer available at the time of completion of KiBS either through PMCT-Plus or the PEPFAR supported Kisumu GAP Care program, the study clinic will continue to provide ARVs to women who met criteria during the study until such programs become available.

Instructions will be given to the mother as to possible side effects and toxicities of ZDV/3TC and either NLF or NVP with particular attention to NVP associated hypersensitivity rash and hepatic toxicity. In the case of generalized rash women on NVP will be instructed to stop the NVP and come to the clinic within 12-24 hours for evaluation. Laboratory monitoring will be done routinely while the patient is on medication and subsequently, if clinically indicated.

<u>Maternal Adherence Assessment.</u>

The mother's adherence to Combivir and NVP or NLF will be measured at every visit by asking her to recall her intake over the previous 3 days. Electronic medication vials (MEMS caps) and pill counts will also help provide a more objective measure of adherence in the breast milk substudy. Counseling regarding the importance of adherence to the regimen will be reinforced at each visit, and women will be counseled about ways to remember to take the medications based on her individual situation. Participants will be encouraged to involve a partner or friend in helping her adhere to the regimen. Home visits will be done weekly during the pregnancy and for the first 2 weeks following delivery to help with adherence to the ARV regimen and to address questions on exclusive breastfeeding. Home visits will be done periodically between clinic visits during the rest of the study to help ensure adherence, follow up and to

address maternal questions related to infant feeding and to treatment. (See Appendix II for Schedule of Evaluations and Home Visits.) Home visits by community health workers are a common feature in this area. The staff doing home visits will address multiple issues, including breastfeeding, weaning, and breast milk substitutes, so women will not be identified as being HIV-positive.

Nevirapine (Infant)

A single dose of NVP oral suspension (2 mg/kg body weight) will be administered by study staff to all infants, preferably within 72 hours of birth, but at a maximum of 7 days after birth. The dose will be given at 72 hours or just prior to discharge from the hospital, if discharge occurs before 72 hours of age. The infant will receive a repeat dose only if vomiting occurs within 30 minutes of dosing. If vomiting occurs after 30 minutes of dosing, no repeat dose will be given. Mothers who deliver at home will be encouraged to bring their infants into the study center as soon as possible within the first week for NVP, BCG and OPV. The infant dose of NVP will be given up to 7 days after delivery.

Preparation

NVP, NLF and Combivir will be prepared in labeled bottles by a pharmacy technician at the CDC Clinical Research Center (CRC) next to the Provincial Hospital. Drugs will be dispensed to the women in the study at the CRC. During antenatal follow-up, a two-week supply of both drugs will be dispensed to mothers at 34, 36, and 38 weeks. Following delivery, mothers will be discharged with a 1-month supply of the study drugs. Home visits will be made by study staff to support mothers in adhering to their antiretroviral drug regimen, to check for drug side effects, and to answer questions by the mother.

7.4.3 Drug Formulation

The maternal Combivir will be supplied as single tablets with 150mg 3TC and 300mg ZDV by Glaxo-Smith-Kline. They can be stored at room temperature.

The maternal NVP doses will be obtained from Boehringer-Ingleheim in 200 mg tablets and can be stored at room temperature.

The NVP oral suspension will be obtained from Boehringer-Ingleheim in a concentration of 10 mg/ml. It can be stored at room temperature but requires protection from light.

Nelfinavir for much of this study has been supplied by Roche using brand drug Viracept, 1250mg BID. In light of the world-wide Nelfinavir recall of all Roche-manufactured product, Nelfinavir was also obtained from Pfizer Pharmaceuticals in the United States also under the same brand name Viracept, 1250mg BID.

Study Drug Accountability

The on-site study investigators are required to maintain complete records of all study drugs received from CDC Atlanta and subsequently dispensed. All unused study drug must be returned to the manufacturer or destroyed according to their specifications after the study is completed or terminated.

7.4.4 Concomitant Medications and Basic Package of Prenatal and Infant Care

Any concomitant medication will be permitted for either the mother or the neonate while in the study with the exception of those listed under study drug dosing exclusions. All concomitant medications will be recorded on the study participant source documents and recorded on the CRF forms if received within six weeks of the receipt of study medications. All immunizations will be given according to the standard of care for pregnant women and children in Kenya.

The basic package of maternal care given as part of the study will include iron, folate, multivitamins, tetanus toxoid, presumptive treatment for malaria with sulfadoxine-pyrimethamine (Fansidar), cotrimoxazole prophylaxis for those with CD4 < 200 or with clinical symptoms of AIDS and CD4 <350, and INH prophylaxis for those with PPD $\geq$ 5mm. Syphilis testing is done routinely at the hospital antenatal clinic. Infants will receive cotrimoxazole prophylaxis starting at 6 weeks, vitamin A, and routine immunizations. CDC will provide medications during the follow up care of the mothers and infants in the Clinical Research Center.

7.4.5 Criteria for Holding Intrapartum Study Drug Dosing or for Study Discontinuation

Maternal

Participants in the study will have study drugs held intrapartum for the conditions listed below until the problem resolves as determined by the study medical officer and the consultant obstetrician.

Pregnancy-induced hypertension requiring intrapartum magnesium sulfate; or severe preeclampsia as defined by blood pressure of $\geq$160 mm Hg systolic or $\geq$110 mm Hg diastolic

Severe maternal infection with sequelae of shock (e.g., ARDS, hypotension)

Intrapartum complication requiring anticoagulation therapy

Intrapartum seizure(s)

Disseminated intravascular coagulation.

Other life threatening conditions

Participants will then be restarted on study drugs post delivery when their condition allows based on the judgment of the study medical officer and the consultant obstetrician.

Once enrolled, patients and their infants will be followed for the length of the study except for voluntary patient withdrawal or if the study is prematurely terminated by the sponsor or appointed safety review group.

<u>Neonate</u>

All single, live births (or the first, live born in a multiple birth) of a participating mother will remain in the study and complete all study monitoring and clinical follow-up and evaluation of study outcomes regardless of where they were born or whether or not the neonate has received NVP. Neonates delivered at PGH or who are born elsewhere but present to the study clinic within 7 days, will not be given the single dose of NVP if any of the following conditions are present at birth or develop prior to dosing.

Neonatal drug dosing contraindications:

Known severe congenital malformations or other condition(s) not compatible with life

Known severe anemia or hypovolemia requiring volume replacement and/or blood product therapy

Known severe hyperbilirubinemia necessitating transfusion or volume replacement. Neonates receiving phototherapy are allowed to receive study drug.

Documented or suspected serious infectious, cardiac, respiratory, or metabolic illness, or other immediate life-threatening condition that does not resolve within 7 days.

SGPT grade 2 ($\geq$ 2.5 times the upper limit of normal) adjusted for age

Inability to tolerate or take oral medications (for NVP dosing)

Note: The initial infant dose of NVP may be held, stored, or kept at the hospital for mothers to pick up or study staff to bring out to the infant, for a maximum of 7 days postpartum. If there are strong indications that a participant is unlikely to deliver at PGH and that the infant may not be seen by study staff within 7 days of birth, infant NVP will be dispensed to the mother to give to the baby at home. In such cases, there may be no blood tests to determine if it is safe to give NVP to the baby.

7.4.6 *Evaluations*

See Appendix II, Schedule of Evaluations

The laboratory evaluations that are included in this study include hematological, biochemical, and HIV related tests. On visits when laboratory tests are done we will take a maximum of 15cc blood from women and 5cc from infants. Many of these tests are routine components of evaluation and monitoring of patients with HIV; others are specific for HIV research. The hematological tests include complete blood count (CBC) and CD4 counts. The biochemistry tests include liver function tests and urine protein and glucose. The HIV related tests include the rapid HIV tests (these are done as part of the Ministry of Health PMCT and VCT programs), HIV DNA PCR and HIV viral load. Most of the laboratory tests are already being done at the CDC/KEMRI labs in Kisian. The capability to determine viral load using Roche kits will be available soon. Testing for viral mutations will be done at CDC labs in Atlanta and elsewhere until the technique is available at CDC/KEMRI labs in Kenya. In order to transfer this technology and

build the CDC/KEMRI laboratory capacity, we plan to sponsor one or two CDC/KEMRI laboratory staff to go to Atlanta to acquire the technical skills to conduct this testing. This training will then be followed up by visits to the CDC/KEMRI laboratory from senior CDC/ATLANTA staff for quality assurance.

Mothers: Evaluations to determine eligibility

Consent will be obtained prior to obtaining blood for determining eligibility. Enrollment in the study will be obtained after eligibility has been confirmed.

Clinical evaluations

+ History [general, potential allergic reactions]
+ HIV counseling
+ Assessment of inclusion and exclusion criteria

Laboratory evaluations

+ Hematology (CBC + diff, platelet count)
+ Screening CD4 count
+ Urine analysis for protein and glucose
+ Serum Chemistries [ALT (SGPT), bilirubin, and creatinine]
+ Hepatitis B surface antigen and Hepatitis C antibody (if CD4 <250; this is done to avoid use of NVP in women with Hepatitis B or C)
+ HIV testing: two rapid tests Determine and Unigold; if discordant, then Capillus rapid test

Mothers: Evaluations at enrollment between 32-35 weeks gestation

After informed consent has been obtained and eligibility has been ascertained including screening CD4 count in order to determine use of NVP versus NLF, the participant can be enrolled in the study. The patient will have an extensive evaluation including demographics, socio-economic status and medical and obstetric history. A complete physical exam will be performed to evaluate for any illness and any AIDS defining conditions.

Enrollment clinical evaluations

+ History [general, potential allergic reactions]
+ HIV-related history and AIDS-defining symptoms
+ Physical exam (including ht, wt, and mid-upper arm circumference (MUAC) for nutritional status assessment
+ Staging of disease based on WHO clinical criteria

Enrollment laboratory evaluations

+ Hematology (CBC, platelet count, MCV, and CD4 cell count)
+ Serum Chemistries (ALT [SGPT], bilirubin, and creatinine, total cholesterol, triglycerides, random blood glucose)
+ Blood smear for malaria
+ Viral load (Stored plasma samples for later assay; 6-7cc total frozen at -70^{0}C)
+ Peripheral Blood Mononuclear Cells (PBMCs)

Mothers: Evaluations at time of HAART initiation at 34 weeks gestation (by 36 weeks latest)

At each visit the patient will be questioned about any new illness or adverse event that may be related to medication. Adherence to the regimen will be evaluated using recall (and medication bottles).

Clinical evaluations including routine antenatal check will be done every week after starting HAART.

+ History (general, potential allergic reactions)
+ HIV-related history and AIDS-defining symptoms
+ Assess adherence to regimen once initiated
+ Physical exam
+ Staging of disease based on WHO clinical criteria (at enrolment and then every alternate visit till delivery)

Laboratory evaluations to be repeated every two weeks until delivery:

+ Hematology (CBC, platelet count, MCV and CD4 cell count)
+ Serum Chemistries (ALT [SGPT}, bilirubin, and creatinine, random blood glucose)
+ Stored plasma samples 6-7cc total (frozen at -70^{0}C)
+ Filter Paper Storage using Dried Blood Spots (DBS)
+ PBMCs

Mothers: Evaluations during/after delivery

Clinical evaluations

- active labor and delivery or within 24 hr postpartum
- 2, 6, 14 weeks, 6, 9, 12, 18 and 24 months postpartum

+ History (general, potential allergic reactions)

+ HIV-related history and AIDS-defining symptoms
+ Adherence to regimen
+ Physical exam [will include ht, wt, MUAC and examination of breasts to assess breast and nipple health]
+ Staging of disease based on WHO clinical criteria

Laboratory evaluations

- active labor and delivery or within 24 hr postpartum
- 2, 6, 14 weeks, 6, 9,12, 18, and 24 months postpartum

+ Hematology (CBC + diff, platelet count, MCV)
+ CD4 cell count [real time but not at 2 weeks]
+ Viral load
+ Serum Chemistries (ALT [SGPT]-real time) and random blood glucose, at above time points till 9 months unless abnormalities noted, in which case follow up as clinically needed
+ Total cholesterol and triglycerides at 6 weeks, 6 months and then if clinically indicated for those on Nelfinavir
+ Filter Paper Storage using Dried Blood Spots (DBS)
+ Stored plasma 6-7cc total (frozen at -70^{0}C)
+ PBMCs
+ Stored breast milk (up to 20 cc total per visit) for 150 women enrolled in breast milk substudy at delivery, 2 weeks, 6 weeks, 14 weeks, and 6 months post partum. In those women that continue to breastfeed after 6 months we will collect breast milk at 9 months, 12 months, 18 months and 24 months.

Preweaning counseling will take place at 5 months

Infants: Clinical and Laboratory Evaluations

Infant Clinical evaluations

- birth (labor and delivery history recorded)
- 1 or 2, 6, 10 and 14 weeks post-birth
- 6, 7, 8, 9, 12, 15,18 and 24 months post-birth

+ History: taken by study nurse
+ Physical exam including weight**,** length and head circumference, MUAC and modified dubowitz or Ballard score (at birth)

For infants in the study, immunizations will be provided through the Provincial Hospital Infant Welfare clinic according to the MOH immunization schedule. Clinical evaluations will be performed by trained medical officers, clinical officers and nurses with an obstetrician and pediatrician as back up. Study assistants will be trained to complete the forms and trained to obtain weight, length, MUAC and head circumference using standardized procedures.

Infant Laboratory evaluations

+ Hematology* (CBC with differential, platelet count)
  - birth or prior to discharge, 6 and 14 weeks, and 6, 12, 18 and 24 months
+ CD4 cell count*
  - 6 wks, and then at 14 weeks, 6, 12, 18 and 24 months if found to be HIV-infected
+ Serum Chemistries* (ALT [SGPT], bilirubin, and creatinine)
  - birth prior to discharge

- at 2, 6 weeks and thereafter only if clinically indicated

+ Qualitative HIV DNA PCR using Dried Blood Spots (DBS) or venipuncture specimens (run after the infant has weaned and mother has stopped ARVs except 14 week and 6 month samples which will be run within 1-2 weeks in order to appropriately advise mother on whether she should discontinue breastfeeding, and to guide clinicians so that timely and appropriate evaluation and care of an HIV infected infant can be done)

  - birth before discharge

  - 2, 6 and 14 weeks post-birth

  - 6, 9, 12, 18 and 24 months post birth (if infected based on two separate positive DNA specimens), will change to quantitative HIV RNA* for all subsequent bloods)

+ HIV antibody EIA* at 18 months, confirmed by repeat EIA test within 1-2 weeks, if no previous positive PCR

+ Filter paper storage using DBS

  -birth, 2, 6, and 14 weeks, 6, 9, 12, 18, and 24 months post-birth

+ Stored plasma and PBMCs (if adequate sample obtained has been obtained for all other tests required for each visit)

  -birth, 2, 6, and 14 weeks, 6, 9, 12, 18, and 24 months post-birth

* Require venipuncture. Bloods for DBS and Hb can be done by heel stick or fingerstick.

Deliveries outside hospital

If the participant delivers at home she will be encouraged to bring the baby to the hospital within the first 7 days. The initial clinical evaluation will be done and blood will be drawn for the first set of laboratory evaluations. The single dose of Nevirapine will be given to the infant up to 7 days after delivery. The mother will also undergo the “delivery” clinical and laboratory evaluations.

Amount of blood to be drawn from infants.

Blood banks typically consider it is safe to withdraw up to 7-9% of whole blood volume on a single occasion in a healthy individual with hematocrit not less than 38% volume. For purposes of this study we will take 3-5 mls of blood (5mls is equivalent to one teaspoon) at visits requiring laboratory tests. This falls safely below 2% of total blood volume for most infants in Kenya, even accounting for varying degrees of anemia and body weight.

Since removal of blood from an iron deficient child could be potentially harmful if the iron deficiency is not appropriately treated, children that undergo venipuncture will receive vitamins with iron supplementation if they are anemic.

We will *not* perform venipuncture on a child with Hb less than 5 g/dL *unless* the blood is removed immediately prior to transfusion.

7.4.7 Sample Storage and testing

The lab studies listed in the protocol and Appendix will be run either real time (e.g. chemistry, hemoglobin, CD4) or as batched runs on specimens stored at the CDC/KEMRI labs in Kisumu (e.g. viral load, infant DNA PCR, RNA PCR). Some specialized testing listed in the protocol such as resistance testing and pharmacokinetics drug levels will be done at CDC Atlanta or other specialized labs when capability does not exist at the KEMRI labs. Attempts will be made to develop capability in Kenya when appropriate, and/or Kenyan laboratory staff will be sent for training.

In addition, a limited amount of plasma, PBMCs, blood, or breast milk samples will also be stored as repository specimens for future studies which may include the following general categories of lab studies: 1)Virologic assays (e.g. viral characteristics, subtype, infectivity); 2) Immunologic studies (e.g. host innate resistance, immunogenetic studies, humoral or cellular immunity); and 3) studies of copathogens and factors related to HIV transmission and disease progression). Participants in the study will be asked to sign a separate consent for storage of samples for future testing [see Appendix VII(v)].

The KEMRI Scientific Steering Committee and Ethics Review Committee will be consulted for approval for any additional test that is not included in the current protocol.

7.4.8 Biohazard containment

As the transmission of HIV and other blood-borne pathogens can occur through contact with contaminated needles, blood, and blood products, appropriate blood and secretion precautions will be employed by all personnel in the drawing of blood and shipping and handling of all specimens for this study, as currently recommended by the Centers for Disease Control.

7.4.9 Counseling

Counseling will be done at each visit. Issues covered will include; proper breastfeeding technique, breast and nipple health and the avoidance of mastitis and nipple cracking, the risk of transmission, the importance of adherence to the medication - stressing the risk of increased resistance and decreased efficacy if the medication is not taken appropriately or if it is shared, the importance of exclusive and appropriate breastfeeding and the importance of weaning at 6 months when the regimen is to be stopped. At the time medications are stopped participants will be counseled about the potential for increased

transmission to the infant if they continue to breastfeed and the increased risk of transmission to the partner if condoms are not used. Counseling on methods to prevent unwanted pregnancies will be offered also. Participants will need counseling and trained staff will be available to support them as they deal with family comment regarding infant feeding decisions such as exclusive breast feeding and early weaning; and help them to work through possible stigmatization as they decide whether to disclose their HIV status to other family members. In an effort to assist with disclosure participants will be encouraged to come to the study clinic with their spouses or other family members. Couple counseling and testing will be offered along with appropriate referral of family members for care and treatment if required. In the event that a spouse or family member is acutely ill, initial care may be offered through the study clinic.

Counseling will take place individually through the clinical staff seeing the participant as well as through more formal group counseling sessions mainly focusing on infant nutrition, exclusive breastfeeding and weaning at 6 months. These sessions will be held by trained CDC counselors at the CDC Research Center and will be open to women participating in both this study and the PMCT evaluation study. Incentives will be provided for women to attend these group sessions.

7.4.10 Weaning

The mothers in the study will be counseled by study staff who have received training in nutrition counseling to wean the infant abruptly over a several week period in the 6th month, a time that is judged nutritionally to be safe for weaning in resource limited settings. Complementary locally available foods will be introduced in the 6th month and the infant will be switched to replacement food entirely by the end of the 6th month. The mother will be counseled and encouraged to minimize breastfeeding to console the infant and have the infant sleep in a separate bed. Occasional home visits will be offered to help with the process and to determine if weaning has taken place. A visit to the study center will be scheduled around 5 months post partum to counsel and provide encouragement for the weaning process. Infants will be

tested for HIV with DNA PCR at 14 weeks and 6 months in order to appropriately advise the mother on whether she should discontinue breastfeeding. Several home visits will be done during and after weaning to monitor the infants health, nutritional status and growth. In the event that weaning or weaned infants are seen to be dropping off the growth curve, the study will intervene on a case-by-case basis with offering nutritional support as determined by the study nutritionist. It may include locally available foods including locally available breast milk substitutes.

As stated above, the supply of water in Kisumu is unreliable and not safe and a high incidence of diarrhea has been noticed in the KiBS infants during the weaning period. The following interventions are to be introduced:

1. Structured and repeated discussion with mothers on food and water hygiene to be done as group and/or individual sessions and to start during antepartum visits and continue through duration of the study (See Appendix XV)
2. Women will be offered a method to purify water; either a locally produced sodium hypochlorite solution, i.e. WaterGuard (dilute bleach) and/or a combination purification/flocculant product called Pur for those women who have turbid water. WaterGuard is a 1% sodium hypochlorite solution (diluted bleach), which is used to disinfect water. The product's shelf life is 12 months if unopened. Pur is a combination of purification and flocculant solution. These products are being promoted widely in the study area; they are relatively inexpensive, and easily available.
3. Women will be offered a safe water vessel (a modified clay pot) with a narrow mouth and lid which does not allow people to contaminate the water by dipping a cup into the water container

7.4.11 Completion of study evaluations

All mothers completing the 24-month evaluation will be considered to have fulfilled the maternal clinical and laboratory evaluation requirements for this study.

All infants completing the 24-month evaluation will be considered to have fulfilled the neonate clinical and laboratory evaluation requirements for this study.

7.4.12 Follow-up

A study home health worker will be assigned to each participant. The caseworker will establish contact with a participant immediately she is enrolled. He/she will be responsible for establishing a relationship with the participant by making home visits. The health worker will help in reminding the participant of upcoming follow-up appointments at the study clinic, finding the participant should she or her infant miss a follow-up appointment, answering questions, helping with adherence to the ARVs, assessing for possible adverse events, and encouraging exclusive breastfeeding and subsequent weaning at 6 months. During the home visits, the follow-up staff will also evaluate water safety in the participant's household both through self reported use of safe vessel and water purification products, and if possible through visual inspection. (See Appendix II for Schedule of Evaluations and Home Visits). In Kisumu, home visits are routinely carried out by health care workers, so visits by the study team's home health care worker will not be perceived as unusual. Confidentiality will be maintained at all times by the home health care worker in the community.

Maximal effort will be made to minimize loss to follow-up. After enrollment, during one of the prenatal visits, women will be accompanied to their home location by a study staff member. A map to the residence will be drawn and kept on file. Because of the highly mobile nature of the population, addresses and directions to the residences of close family members and friends with whom the study participant may live or visit in future will be collected, filed and updated regularly. If a participant moves out of Kisumu, every effort will be made to find her and her infant through the addresses she provided and through friend and family contacts she provided to the study staff. Additionally, the participant's

photograph (taken at enrollment) will be used to track her in the community. Photographs have been used for verification and tracking of participants in other studies conducted in the same facility and are considered culturally acceptable.

7.4.13 Consent Process

Participants who express interest in participating in the study will sign consent after initial screening has been performed. Enrollment in the study will depend on signing the consent and meeting clinical and laboratory criteria. The consent form (see Appendix VII) will be reviewed with the woman being consented by a staff member in the language the participant is most comfortable with. In addition, a summary document including risks, benefits, and the schedule of follow-up visits (Key Facts Sheet) will be reviewed and given to the potential participant. She will have the opportunity to have any questions answered. Her understanding of the study will be assessed through questions asked by the staff consenting her. When ready, the volunteer, in the presence of a witness, will sign the document, giving her consent and understanding to voluntarily participate in the trial with the knowledge that she may terminate her participation at any time.

If the woman is less than 18 years old but at least 15 years and she wants to participate in the study, she will be advised to come to appointments with a parent or guardian. The parent or guardian will be required to give consent for her participation and the minor must give her assent.

With the woman's agreement, the verbal consent of the husband will be sought. Compliance with the study may be better if the spouse is informed, however his consent will not be required. In regard to the infant there is no legal requirement to have paternal consent in Kenya or in the United States when there is clear benefit to the infant. Requiring paternal consent would also compromise the participant's confidentiality

7.4.14 Toxicity Management of Mothers and Infants

General Guidance on Management of an Adverse Event

The women and their infants will all live in the Kisumu area and are anticipated to be able to access study personnel if medical problems arise outside of their scheduled visits. Criteria for toxicity are based upon the National Institute of Health/Division of AIDS (NIH/DAIDS) Toxicity Tables for neonates, children and adults. Normal ranges for laboratory values will be the institutional values at the CDC/KEMRI laboratory at Kisian. Abnormal test values must be repeated to confirm results (preferably within 72 hours, but no more than one week).
Management of the adverse event will be according to best clinical practice and the judgment of the on site physician. All clinical and laboratory adverse events will be followed closely by study staff. Follow-up of clinical adverse events will depend on the seriousness and severity of the event. All patients requiring hospitalization will be visited daily by study staff in order to document progression/resolution of the adverse event as well as to provide any clinical services/pharmaceutical support deemed necessary. Less serious adverse events will be monitored by study staff in the research center with outpatient management as deemed appropriate by the study physician. Abnormal laboratory evaluations (any grade) determined by onsite staff not to be clinically significant will be repeated at the next clinic visit to verify resolution. For any clinically significant abnormal laboratory evaluations, the mother and/or infant will be traced by members of the study staff and will be brought back to the clinic for further evaluation, treatment (if indicated), and repeat laboratory evaluation.

Alternative explanations for clinical or laboratory abnormalities must be sought. If study drug is discontinued due to Grade 3 toxicities that are subsequently attributed to underlying infections, other processes, or to medications other than the study drugs, the study drug can be restarted if done so within

14 days of the last dose, after discussion between the PI and the Atlanta Medical Officer.

For subjects receiving NVP, dose escalation to 200 mg BID at the beginning of week 3 must not occur if any toxicity is detected or suspected at the week 2 study visit.

General Guidelines for decisions regarding drug toxicity

Grade 1: Continue study drug.

Grade 2: Continue study drug.

Grade 3: Continue study drug while confirming abnormal test results preferably within 72 hours but no more than one week . If repeat assessment confirms Grade 3 toxicity possibly related to the study drug, hold study drug for up to 7 days and reassess.

- *If repeat assessment or value is Grade 2 or less; Study drug may be restarted. If Grade 3 or higher toxicity recurs, the study drug should be permanently discontinued.*
- *If repeat assessment or value is Grade 3 or more; Study drug should continue to be held and reassessed within another 7 days (for a total of 14 days). If toxicity is Grade 2 or less, restart study drug; if toxicity at 14 days remains or recurs at Grade 3 or higher, study drug should be permanently discontinued and the woman should be switched to another highly active combination regimen based on her physician's recommendation.*

Grade 4: Hold study drug pending confirmatory test preferably within 72 hours but no more than one week. If repeat assessment confirms Grade 4 toxicity, the study drug should be permanently discontinued; and the woman should be switched to another highly active combination regimen based on her physician's recommendation.

Management of Maternal adverse events other than Hepatic Toxicity or Rash

Follow NIH/DAIDS Adult Toxicity Tables to determine grading of toxicity. See Appendix IV for specific toxicities. The action taken will be as per the general guidelines above.

Grading of Maternal Laboratory and /or Clinical Hepatic Toxicity late gestation or postpartum

For women on NVP, if grade 2 or higher hepatic toxicity is found at any visit, NVP study drug will be discontinued immediately and if grade 2 or higher hepatic toxicity is confirmed at 72 hours, NVP will be permanently discontinued. In addition, if grade 1 hepatic toxicity occurs in a patient with any clinical symptoms suggestive of hepatic toxicity (including malaise, jaundice, pale stool, fatigue, fatigue, nausea and vomiting, or liver enlargement or tenderness) or hypersensitivity reactions (new or worsening rash, irritation of mouth, eyes or other membranes, study drug NVP will be permanently stopped. Subjects with normal LFT's who develop symptomatic clinical hepatitis must also permanently stop study medication NVP immediately.

Grading and Management of Maternal Rash late gestation or postpartum

Use Toxicity Table II-D to grade rash. Stop maternal NVP drug permanently for Grade II B or above rash.

Maternal rash should be graded and the woman managed as stated below (see Appendix IV D for grading criteria):

♦ Grade 1 (erythema with or without pruritus) to Grade 2A (diffuse erythematous macular or maculopapular rash or dry desquamation with or without pruritus but without constitutional findings, or target lesions without blister/vesicle or ulceration in lesion): Prompt evaluation as soon as toxicity is suspected. Study drug should be continued. Antihistamines may be used to try to prevent progression of the rash. Laboratory studies including liver function tests, CBC with differential, and Creatinine will be obtained. If rash occurs during first 2 weeks of study treatment, protocol-specified increase in study drug dose may not occur until the cutaneous eruption has resolved. If the rash does not resolve within 14 days of onset, the Atlanta Medical Officer will be notified. NVP study drug will be discontinued permanently if a grade 2B urticarial rash develops or if a rash is associated with a documented grade 2 or higher hepatic toxicity.

♦ Grade 2B or higher (urticaria): Study drug should be permanently discontinued.

All Grade 2B, 3 and 4 skin rashes will be reported by the study team to the PI or designee within 48 hours of receiving information on the event. An adverse experience report will then be sent to the CDC Atlanta database within 48 hours of the event with the appropriate case report form. Appendix IVD

(Supplemental Toxicity Table for Grading Severity of Cutaneous/Skin Rash/Dermatitis Adverse Experiences and the Rash Management Guidelines) MUST be used for grading these toxicities.

Toxicity Management in the Infant

No Maternal Dose Modification

In most cases maternal doses of antiretrovirals will not be modified for potential infant toxicities related to exposure through breast milk, because the relatively low drug concentrations in breast milk are unlikely to cause anything but an idiosyncratic reaction in terms of infant toxicity. Infant dosages will not be readily determinable. If a maternal drug (s) is judged probably or definitely related to infant toxicity, the clinician may switch the maternal drug(s) included as part of the maternal HAART regimen based on best clinical judgment.

General Criteria for Management of Infant Toxicity

Criteria for toxicity grading are based upon the age-related DAIDS Toxicity Tables for infants ≤3 months and children >3 months of age (SSP manual) with the exception of rash and hepatotoxicity (see specific sections for grading and management).

The normal range for laboratory values will be those used by the CDC/KEMRI lab. Abnormal test values must be repeated preferably within 72 hours but no more than one week to confirm the result. Management of any adverse event will be according to the best clinical practice available and the judgment of the site investigator.

Alternative explanations for clinical or laboratory abnormalities must be sought. If maternal study drug is

discontinued due to infant Grade 3 toxicities (grade 2B or above for skin rash); and in the unlikely case that infant toxicity is judged to be probably or definitely related to maternal study drug, then the maternal study drug regimen should be changed based on the clinical judgment of the study physician AFTER discussion with the Protocol Team and Atlanta Medical Officer.

Infants whose mothers are on study drugs should be managed as follows:

- Grade 1: Continue maternal study drug.

- Grade 2: Continue maternal study drug.

- Grade 3: Study drug can be continued while awaiting confirmation of abnormal test preferably within 72 hours, but no more than one week. If repeat assessment confirms Grade 3 toxicity and in the rare occasion this is judged by the clinician, Kisumu PI and the Atlanta Medical Officer as likely related to maternal study drug, then the maternal study drug will be changed according to the clinician's judgment. If the toxicity is not deemed probably or likely related to maternal study drug then the mother continues study drug and the infant is managed clinically

Follow-up

Any infant whose mother has received study drug will be followed for 24 months as scheduled irrespective of any changes or premature discontinuation of the mother's drug regimen.

Grading of Infant Laboratory or Clinical Hepatic Toxicity

For the purposes of this study, the following grading system for ALT will be used for all infants (regardless of age):

♦ Grade 1: ≤4.9 times the upper limit of normal for ALT

♦ Grade 2 and above: ≥5 times the upper limit of normal for ALT

Clinical hepatitis is defined as clinical signs and symptoms of clinical hepatic dysfunction, regardless of ALT values, including enlarged liver (>4 cm below right costal margin), hepatic tenderness, ascites, portal hypertension (e.g., varices, splenomegaly, caput medusae), or hepatic encephalopathy (e.g., asterixis, changes in level of consciousness).

Management of infant laboratory or clinical hepatic toxicity should be as follows:

ALT Toxicity

If result of the infant laboratory ALT drawn is Grade 2 or above (i.e. ≥5 times the upper limit of normal) study staff should notify the clinician, and PI in Kisumu within 2 working days. If the PI or their designee judge the laboratory result may be possibly, probably or definitely related to study drug, they will forward a report, by fax or email, to the Atlanta Medical Officer for determination as to relation to study drug within 2 working days (or ASAP). In the unlikely event the laboratory toxicity is judged related to maternal study drug, then the mother's study drugs should be switched and the infant's laboratory and clinical status reassessed after one week.

Clinical hepatitis

If suspected clinical hepatitis in the infant is confirmed, the study staff should notify the study physician and the PI or designee in Kisumu within 2 working days. If the physician and PI judge the clinical finding

or laboratory result may be possibly, probably or definitely related to study drug, the PI or designee will forward a report within 2 working days of receipt of notification both by fax and email to the Atlanta Medical Officer for determination as to relation to study drug. In the unlikely event the infant hepatic toxicity is judged probably or definitely related to maternal drug, then the mother's study drugs should be switched according to the clinical judgment of the study physician.

If clinical hepatitis is ruled out, no change to maternal study drug is needed.

Follow-up

Any infant that has been enrolled in the study irrespective of changes to maternal antiretroviral drug regimen will be followed up for 24 months as scheduled.

Management and Grading for Infant Cutaneous/Skin Rash/Dermatitis Adverse Experiences

General Guidance for Grading Severity of Infant Rash

Appendix IVD, the Supplemental Toxicity Table for Grading Severity of Cutaneous/Skin Rash/Dermatitis Adverse Experiences and the Rash Management Guidelines from the standard AIDS Clinical Trial Group Toxicity Tables and are to be followed for grading any cutaneous adverse event.

Severe rash is a known side effect of NVP and generally not associated with 3TC/ZDV. In this study, the clinical team (medical officer, clinical officer, and PI) will assume that all severe rashes are a result of infant NVP exposure unless clinical findings indicate otherwise.

For any grade rash, treatment should be provided. Pruritus or rash with pruritus and minor accompanying symptoms should be managed with antihistamines, antipyretics and/or non-steroidal anti-inflammatory medications according to the judgment of the on-site clinician. These will be supplied through the study.

A prodromal syndrome of new onset consisting of clinical symptom complex manifested as fever (> 39°C oral), malaise, cough, lymphadenopathy, edema, myalgia, and/or arthralgia may occur before the development of cutaneous manifestations of Stevens Johnson Syndrome (SJS), Toxic Epidermal Necrolysis (TEN), or the SJS/TEN Overlap Syndrome. Therefore the presence of constitutional symptoms makes the rash Grade 3 or 4 (see Appendix IVD).

<u>Grading and Management of Infant Rash</u>

Rash should be graded and the patient be managed as stated below (see Appendix IV D for grading criteria). All references to maternal study drug in this section refer to NVP, unless otherwise indicated.

♦ Grade 1 (erythema with or without pruritus) to Grade 2A (diffuse erythematous macular or maculopapular rash or dry desquamation with or without pruritus but without constitutional findings, or target lesions without blister/vesicle or ulceration in lesion): Prompt evaluation as soon as toxicity is suspected. Study drug should be continued. Antihistamines may be used to try to prevent progression of the rash. Laboratory studies including liver function tests, CBC with differential, and Creatinine will be obtained. If rash occurs during first 2 weeks of study treatment, protocol-specified increase in study drug dose may not occur until the cutaneous eruption has resolved. If rash does not resolve within 14 days of onset, the Atlanta Medical Officer will be notified. NVP study drug will be discontinued permanently if a grade 2B urticarial rash develops or if a rash is associated with a documented grade 2 or higher hepatic toxicity.

- o Grade 2B (urticaria), or higher: Maternal study drug, NVP, should be permanently discontinued. The clinical team will consider switching the mother to Nelfinavir (NLF).

All Grade 2B, 3 and 4 skin rashes will be reported by the study team to the PI or his designee within 2 working days of receiving information on the event. The PI in Kisumu will then send an adverse experience report to the Atlanta CDC database and the Atlanta Medical Officer by both email and fax within 2 working days of notification of the event with the appropriate case report form. Appendix IVD (Supplemental Toxicity Table for Grading Severity of Cutaneous/Skin Rash/Dermatitis Adverse Experiences and the Rash Management Guidelines) MUST be used for grading these toxicities.

Follow-up

Any infant enrolled in the study who has been exposed to maternal antiretrovirals drugs will be followed for 24 months as scheduled.

Concomitant Medications

Medications used for the treatment of adverse events that occur during study participation must recorded on applicable study case report forms.

7.4.15 Criteria for Discontinuation of ARVs (Mother and Neonate)

Combivir and/or either NLF or NVP may be switched to an alternative highly active regimen or discontinued based on intolerance or adverse reaction as described above upon the recommendation of the study physician and after consultation with the Kisumu PI and the Atlanta Medical Officer and

discussion with the mother. Management guidelines described in the previous section should be followed.

Treatment discontinuation

Mothers for whom the study drug (s) is/are prematurely discontinued due to toxicity or intolerance will continue to be followed in the study and be offered an alternative regimen of highly active combination antiretrovirals. They will be followed for all the clinical and laboratory evaluations scheduled for the study through 24 months postpartum. In the event that no ARVs combinations are tolerated, the mother will be advised to discontinue breastfeeding and to switch to replacement foods.

8. DATA MANAGEMENT

Customized Case Report Forms (CRF) will be developed and used for recording data on each enrolled study subject. Subjects will not be identified by name on any study documents. Subjects will be identified by a screening identification number upon study screening and subsequently identified upon enrollment by a unique Study Identification Number (SID) assigned on site by the Data Coordinating Center. Upon assignment all future patient identification will be through the use of self-adhesive pre-printed number labels to insure confidentiality and reduce transcription errors. The mother and infant identification numbers will contain the same root identification number with the addition of an identifier digit corresponding to the mother (0), the first born baby (1), second born baby (2) etc and a check digit. (Analysis of multiple births will be based on the first born only, and subsequent pregnancies will not be eligible for the study).

All data on the CRF must be legibly recorded in ink (or typed). Subsequent corrections will be made only by marking through the incorrect entry with a single line, entering the correct information, initials of

writer, and date of correction adjacent to the incorrect entry on the original. Any required information that is not obtained as specified in the protocol should have an explanation noted on the CRF as to why the required information was not obtained.

CRFs will be entered using an automated teleforms system. This data management system utilizes optical character recognition (OCR) software to interpret hand printed, machine printed, mark sense fields and bar codes from completed forms, reducing the need for direct data entry of the CRFs. All forms will be visually scanned after character recognition to identify any discrepant results between hard-copy form and the character-recognized fields. The teleforms system will reduce CRF processing time during day to day management of the trial by providing immediate fax processing by computer, computer assisted review of CRFs, integrated document imaging and database management systems, standardized quality control reports (including flagging and tracking CRF problems like missing data, apparent recording errors, ambiguous entries, overdue visits etc.), and automated work flow management. The entire database and CRF images will be automatically stored on backup tapes that will be kept at the CDC clinic and CDC Epidemiology Branch, DHAP, NCHSTP, Atlanta. GA.

Instructions concerning the recording of study data will be provided in the Manual of Operations.

8.1 Quality Control

Site Monitoring

In addition to performing quality control at the CDC field station in Kisumu, site visits by Atlanta CDC staff will be made in order to monitor the quality of data collected in the research records, the accuracy of the data entered in the database, and to determine that all regulatory requirements surrounding the trial are

met. The PI and staff will allow independent monitors supported by CDC to review consent, charts, laboratory procedures, standard operating procedures, and upon request the Kenyan Ministry of Health, to inspect study documents (e.g., consent forms, drug distribution forms, case report forms) and pertinent hospital or clinic records for confirmation of the study data. In addition, Good Clinical Practice (GCP) and Good Laboratory Practices (GLP) training will be done for site staff and they will likewise be instructed on approaches to ongoing internal monitoring of data collection and study procedures.

Data Management

Interviewer training will stress the need for accurate reporting. The questionnaires themselves have internal consistency checks. Key pieces of information will be verified at every follow-up visit. Data entry will incorporate consistency, range and other checks. The data manager will run regular checks and any queries will be sent to the field. There will be a general training for all study personnel prior to beginning the protocol. Study personnel will be given an operation manual to which they can refer for further operational details in running the study. We will pilot the study forms before initiating data collection. Community health workers, study coordinators, and laboratory personnel will all be appropriately trained regarding the protocol requirements and a standard operations manual will be available.

Laboratory

The Kisian laboratory quality assurance (QA) will encompass all measures taken at the pre-analytic, analytic and post-analytic stages of the testing process. This will ensure reliability of the investigation by selecting and obtaining satisfactory samples from the client or patient, analyzing the samples and recording test results promptly and correctly, and appropriately interpreting and reporting results with all procedures documented. The essential elements of the laboratory QA will be internal quality control (IQC) and external quality assessment (EQA) or proficiency testing (PT). IQC controls for reproducibility

of test results will be generated in an individual laboratory by assessing, in real time, the precision of test results. IQC will first provide training to the laboratory staff to acquaint them with the relevant technical skills tailored to meet specific requirements of the investigative process. Secondly, it will provide Standard Operating Procedures (SOP) succinctly detailing technical procedures involved (e.g. sample collection, processing and storage, assay procedures and how to interpret test results), all geared to limiting errors due to technician bias. In order to further minimize technician bias, there will also be independent interpretation of test results by more than one technician. The other component of IQC will be proper maintenance of records relating to a given project. The records will include test requisition forms, questionnaires, specimen logbooks, laboratory workbooks/sheets, technical procedure documents, double entry of test results, sample tracking sheets, shipment details, equipment maintenance and calibration records, and personnel and quality improvement plans. Finally, IQC will include evaluation of laboratory personnel competency on a regular basis regarding laboratory technical procedures, quality control measures and safety precautions. In addition, the Kisian Laboratory QA program will include EQA, allowing comparison of results obtained in the Kisian laboratory to those of an external reference laboratory. One way of achieving this will be a system where a central laboratory sends out challenge specimens for testing to other laboratories participating in the EQA exercise. Results obtained by different laboratories will be evaluated; scores will be sent to the individual laboratories. An alternative approach will be a system whereby specimens submitted to an individual laboratory will be split and exchanged with a reference laboratory. The reference laboratory will perform a similar test on the specimen, and the results will be compared.

8.2 Data Analysis

Mothers and infants will be followed through 24 months of age, unless otherwise determined by the Safety Review Group. Children for whom no HIV test result is available will be excluded from the

analysis of transmission risk and will also be excluded from the analysis of HIV-free survival, if not known dead. Pregnancies that yield multiple births will be considered as one unit when evaluating for transmission, i.e., if any one of the infants becomes infected we will consider that to be a transmission. All the infants will be included in the study. Fetal deaths in utero and stillbirths will be excluded from the analysis of the cumulative risk of infection. Stillbirths will be included in the analysis of HIV-free survival. Statistical significance is set at $p<0.05$.

Cumulative risk of infection and HIV-free survival probability will be estimated using the Kaplan-Meier method or other survival analysis methods, such as COX proportional hazards regression, as appropriate. For estimating the cumulative infection risk, the age at event for infected children will be the midpoint between the ages at last negative and first positive tests, or, if no negative test, half the age at first positive test. Children not known to be infected will be censored at the age of last negative test. For estimating HIV-free survival, the age at event for children who die without having a positive test will be the midpoint between the ages at last negative test and death, or, if no negative test, half the age at death. Stillborn children will be assumed to die at age 0 days. The same method will be used to estimate cumulative infection risk and HIV-free survival for the HIVNET 012 trial.

We will use a two-sample Z-statistic to test the efficacy of the study regimen against the single dose NVP regimen used in HIVNET 012. If p_1 and v_1 are the estimates of cumulative infection risk and its variance, respectively, in this study, and p_0 and v_0 are the corresponding estimates from HIVNET 012, then the test statistic is

$$Z = \frac{(p_1 - p_0)}{\sqrt{(v_1 + v_0)}}$$

Under the null hypothesis of the same cumulative risk in the two studies, the probability distribution of Z is approximately that of a standard normal distribution. We will also perform one-sample tests to compare the cumulative infection risk in this trial to theoretical thresholds of interests. In addition, we will use exact binomial methods to calculate more precise versions of these z-statistic tests, which are based on the normal approximation to the binomial distribution, when appropriate.

Use of the Kaplan-Meier method allows us to use standard methods (such as the Lan-DeMets procedure) for interim data analysis while preserving the overall Type I error ($(p_1 - p_0) / (v_1 + v_0)$ satisfies the necessary hypothesis for use of this procedure). The efficacy endpoint data are actually interval-censored, because the exact age at which a child would first test positive for HIV is not known unless there is a positive test at birth. Experience with other similar studies shows that this Kaplan-Meier procedure (ignoring the interval-censoring) should provide correct point estimates and confidence intervals. However, we will use Turnbull's extension[50] of the Kaplan-Meier procedure to interval-censored data to verify the point estimates and standard errors for both cumulative infection risk and for HIV-free survival.

In addition, age at weaning should be considered to be a competing risk for HIV infection, because a weaned child should test positive at most 60 days after weaning. Failure to account for weaning will affect point estimates and standard errors if some weaned children are not tested by the end of the study or if some children without a positive test are lost to follow-up before weaning. Again, experience with other similar studies shows that the simple Kaplan-Meier procedure (ignoring both interval-censoring and weaning as a competing risk) should provide correct point estimates and confidence intervals. Therefore we will also use competing risk methods[51] to verify point estimates and estimates of standard errors. Cox Proportional Hazards models will be used to adjust for potential confounders in a multivariable model, while censoring follow-up time and evaluating the primary endpoint by type of intervention. Weighted Kaplan-Meier or time-varying Cox models will be used if the hazards are not proportional.

Analyses for secondary endpoints will be performed at the appropriate times based on the endpoint. Rates of immunologic and virologic progression (CD4 cell and RNA changes) by type of intervention will be assessed by both absolute change and percentage change from baseline to subsequent measures using basic statistics, as well as repeated measures designed to control inter-subject variability.

Amendment considerations of sample size given the change in study design

1. Sample size and power calculations.

Historically, the cumulative rates of HIV transmission in the HIVNET 012 study in the NVP arm were 16% at 18 months of age. We first wished to be able to detect a 50% reduction in the cumulative perinatal HIV transmission rate at 18 months with the new regimen of either NVP-HAART or NLF-HAART when compared to the HIVNET 012 transmission rate.

We performed exact binomial one-sided power calculations with a variety of total sample sizes (n) in the new regimen, using a significance level of $\alpha = 0.025$. We computed the lower critical values (m) of observed transmission events in the new regimen, below which we reject the null hypothesis of equality in favor of the alternative hypothesis that the new regimen significantly reduced the transmission rate. For example, for a total sample size of $n = 350$ in the new regimen, observing $m = 42$ or fewer transmissions would be significant evidence in favor of the alternative hypothesis.

We also recognized the importance of inflating the calculated sample sizes to account for losses in evaluable subjects, due to drop-out (estimated at 10%) and changes in treatment, among other causes. Likewise, we recognized that the binomial assumption does not account for the significant variability due to heterogeneity among the subjects and in actual treatment over time. Therefore, it is necessary to inflate the calculated sample sizes by a factor of $1/(1 - d)$, where d is the discounted proportion of subjects who are not directly evaluable. For example, a calculated sample size of $n = 350$ would need to be increased to $n = 389$ or 437 with a discounted

proportion of d = 0.1 or 0.2, respectively. Thus, the intended sample size of n = 480 should allow for this hypothesis to be tested successfully, even with drop-out and the additional variability in the study.

Table 1. Power to reject the null hypothesis for various sample sizes and alternative transmission rates, with a one-sided significance level of α = 0.025.

	d = 0.2	n = 250	n = 313	n = 375	n = 437
	d = 0.1	n = 223	n = 278	n = 334	n = 389
	d = 0.0	n = 200	n = 250	n = 300	n = 350
		m = 21	m = 28	m = 35	m = 42
π_A = 0.14		0.089	0.116	0.139	0.158
π_A = 0.12		0.300	0.394	0.474	0.541
π_A = 0.10		0.648	0.774	0.855	0.907
π_A = 0.08		0.920	0.971	0.990	0.996
π_A = 0.06		0.995	0.999	1.000	1.000

Second, we wished to be able to compare the rates of hepatic, rash, and other severe adverse events (SAEs) requiring changes in treatment among those subjects treated with NVP-HAART and NLF-HAART in the current study. The time-frame for these SAEs to occur is from the start of treatment to six months post-partum, for a total of about 7 months on a particular regimen. Specifically, we wished to be able to detect a difference in the rates of SAEs in the NLF-HAART regimen, thought to be about 1%-2%, and in the NVP-HAART regimen, thought to be about 8%. This comparison will be made between subjects on the two regimens with CD4 counts greater than 250/mm^3 (approximately 75% of subjects).

We used the standard Normal approximation formula for these power calculations using a two-sided significance level of α = 0.05. For example, with 1% and 8% SAE rates in the two arms, a sample size of n = 175 in *each* arm would give 89.3% power to reject the null hypothesis of equality in SAE rates between both arms. Thus, a total of 350 subjects with CD4 counts

greater than 250/mm³ would be necessary, which reflects a total of 468 subjects in the whole study (regardless of CD4 count).

We also recognized the importance of inflating the calculated sample sizes to allow for losses in evaluable subjects and additional variability in the study, as described above. For example, a calculated sample size of n = 175 in each arm would need to be increased to n = 195 or 219 with a discount rate of d = 0.1 or 0.2, respectively. Thus, a total of 390 or 438 subjects with CD4 counts greater than 250/mm³ would be necessary, which reflects a total of 520 or 584 subjects in the whole study (regardless of CD4 count). We have chosen a sample size based on the current loss to follow up of less than 10%.

Table 2. Powers to detect differences between the rates of hepatic, rash, and other SAEs for the NVP and NLF treatments for various sample sizes in *each* arm ($n_1 = n_2 = n$).

	d = 0.2	n = 213	n = 219	n = 250	n = 282	n = 313
	d = 0.1	n = 189	n = 195	n = 223	n = 250	n = 278
	d = 0.0	n = 170	n = 175	n = 200	n = 225	n = 250
$\pi_1 = 0.01,\ \pi_2 = 0.08$		0.879	0.893	0.925	0.950	0.967
$\pi_1 = 0.01,\ \pi_2 = 0.07$		0.809	0.826	0.868	0.904	0.931
$\pi_1 = 0.02,\ \pi_2 = 0.08$		0.720	0.739	0.788	0.834	0.870
$\pi_1 = 0.02,\ \pi_2 = 0.07$		0.605	0.623	0.676	0.727	0.771

[Calculations based on Fleiss (1981), p. 41, eqtn (3.12).]

It should be noted that these sample size and power calculations are based on standard simplifying assumptions, such as that the transmission rate is well-modeled by a binomial proportion at particular times of interest. Nevertheless, the actual analyses of the study data will use survival analysis methods, which account for the censoring of subjects and adjusts for the inclusion of covariates of interest (e.g., baseline CD4 count or viral load, either as categorical or continuous variables). Furthermore we will present results regarding the estimated risk of the outcomes of transmission or infant HIV free survival by co-variates of interest, such as CD4 stratum categories, and assigned maternal drug (NLF or NVP based HAART).

8.3 Data Monitoring

Treatment toxicity will be monitored on an on-going basis as per protocol. A look at safety and tolerance will be done after the first 50 women have been followed through 6 weeks postpartum. Follow-up safety analyses and measures of loss to follow-up will be reviewed every 6 months by a CDC appointed Safety Review Group which will include the CDC-Atlanta medical officer assigned to the protocol, an independent statistician, the PI in Kisumu, a medical representative from the Provincial MOH, an independent KEMRI scientist, and 1-2 other experts appointed by the CDC branch chief. Information on the number of women screened, number found to be eligible, number enrolled, and rate of follow-up, will be reviewed by the Safety Review Group every 6 months or more often if required by the group.

One formal interim analysis of the transmission endpoints is planned. This will be done when the first 200 infants enrolled have reached nine months of age. Recommendations for early termination of positive or negative results will be guided by the symmetric group sequential O'Brien-Fleming boundary method. The O'Brien-Fleming design allows for early termination if extreme initial results are observed, while preserving the standard single one-sided .025 level of the Kaplan-Meier statistic at the final analysis with no increase in sample size. The Lan-DeMets spending implementation of the O'Brien-Fleming use function will be employed to define proper significance levels at the time of the formal interim analysis.

If analyses demonstrate unexpected safety concerns or lack of efficacy of the HAART regimen, the study would be concluded prematurely and mothers and infants would only be seen at the next scheduled visit and at the 24 month visit at which time plasma would be obtained for the appropriate diagnosis of HIV infection status.

<u>Guidelines for modifying the trial drug regimen or stopping the trial for safety concerns</u>:

The protocol team considers that a doubling of the rate of maternal serious adverse events (>20%) compared to that reported in other recent trials (8-12%) using the ZDV/3TC/NVP regimen in the U.S., Europe and Africa would be of sufficient concern to warrant the Safety Review Group recommending either modifying the study drug regimen being used in the KiBS trial or recommending stopping of the trial. Likewise documentation of a maternal death rate due to fulminant liver failure that exceeds the rates reported in these other studies (range 0.4%-0.04%) should also be considered sufficient reason for the Safety Review Group to recommend either modifying the study drug regimen in KiBS or recommending stopping of the trial.

Given the January 19th 2005 FDA advisory and change to the BI label for NVP, the KiBS protocol team had modified the protocol so that any women who are enrolled for the remainder of the KiBS study who have CD4 counts greater than or equal to 250/mm³ at screening will be started on NLF instead of NVP. There are no longer any women in KiBS with CD4 ≥250 who fall within the first 6 weeks of NVP, the highest risk period for hypersensitivity reaction. Any enrolled women already on NVP for greater than 6 weeks who later demonstrate any symptoms or signs of clinical hepatitis, hepatic grade II asymptomatic liver elevation, or urticarial 2B rash will be switched to NLF according to the hepatic and rash toxicity management section of the protocol (7.4.14). Given the June 2007 Roche global recall of Nelfinavir, all remaining participants were placed on either Nelfinavir obtained from Pfizer Pharmaceuticals in the United States or Lopinavir/ritonavir.

8.4 Adverse Event Reporting

An adverse event (AE) is defined as any health-related reaction, effect, toxicity or abnormal laboratory result that a study participant experiences during the course of a study irrespective of relationship to the study treatment intervention. This includes changes in a participant's condition or laboratory results that

have or could have a deleterious effect on a participant's health or well-being. A serious adverse event (SAE) is defined as any experience that is fatal or life-threatening, requires in-patient hospitalization or prolongation of an existing hospitalization, results in a persistent or significant disability or incapacity, is a congenital anomaly or brain defect, or cancer (except AIDS-associated malignancies). The severity of adverse experiences will be graded using standard DAIDS toxicity tables (see appendix IV). All grade 3-4 toxicities are considered serious. In addition, rash grade $\geq$2B and hepatotoxicity grade $\geq$2 will be considered serious. Reporting procedures for SAEs are detailed in the Study Operations Manual and the procedures are summarized below. Appendix V contains a flow diagram for adverse event reporting.

Unanticipated SAE's (except those judged definitely unrelated) will be <u>reported</u> by the PI or designee in an expedited manner to the Atlanta Medical Officer. The PI or designee will prepare an SAE Site Summary Form and complete a draft of the CDC "Human Subjects Adverse Event Report" form (Appendix VI). These two forms will be sent electronically within 2 working days of the initial notification using email and/or a secure data network (SDN) set up by the Epidemiology Branch, NCHSTP**.** The Atlanta Medical Officer will then make the final independent judgment as to the severity, relatedness, and anticipated or unanticipated nature of the SAE and finalize the "Human Subjects Adverse Event Report" form. If the event is determined to be serious, unanticipated, and the relationship is anything except "unrelated", then the Atlanta medical officer will <u>report</u> the event with 2 working days of notification directly to the Human Subjects Contact and CDC IRB with a copy to the Epidemiology Branch Chief and Division ADS. If the event is reported to the CDC IRB, then the Kenyan IRB will be simultaneously notified. This expedited reporting will occur if the adverse event occurs during the period the mother is on drug and also during the 8 week period following the last dose of study treatment. SAEs meeting the requirements for expedited testing will be reported to the IRBs as soon as possible with the goal of doing this within 10 working days. In all cases of SAEs, the information (irrespective of relation to drug or unanticipated or anticipated nature of event) will also be recorded as a diagnosis or lab value on the relevant case report form (CRF) which goes into the study database and which will be sent as a

summary report to the CDC IRB every 4-6 months along with the safety review group comments/recommendations based on the safety data summary.

Any new SAEs among mothers or their infants that occur more than 8 weeks after the last dose of study drug (generally at 8-9 months postpartum) will be considered unrelated to study drug unless otherwise judged by the PI and Atlanta Medical Officer.

Summaries of adverse events for mothers and their infants (both serious adverse events through 24 months) will be generated by the study team statistician from both the case report forms entered into the protocol data base and the SAE data recorded in the SAE reporting system. These summary reports of SAEs will be submitted to the Kenya Medical Research Institute (KEMRI) Ethical Review Committee and the U.S. CDC IRB and the CDC Safety Review Group according to their individual requirements (CDC Atlanta IRB and Safety Review Group at least every 4- 6 months, but more often if requested) but no less frequently than every 12 months.

Participants requiring emergency medical care will be referred to the Kisumu Provincial or District Hospital, or other medical facility if appropriate care is not available at these hospitals. The study clinic will also provide basic medications, immunizations, and routine and urgent care services during working hours to manage intercurrent minor illnesses.
About 30% of all KiBS infant hospitalizations are due to malaria. We will be offering women impregnated bed nets and assessing their use in the homes during follow-up visits.

9. TIME FRAME/DURATION OF PROJECT

Enrollment is estimated to occur over 24-30 months from both hospitals until the sample size of 520

mother-infant pairs is reached. Each mother-infant pair will be followed for 24 months postpartum. The entire length of the study will therefore span approximately 54 months. Prior to enrollment of participants, the focus group studies will be held to get direct feedback from the community. The entire focus group study including the analysis will take about 2 months. (See Appendix X for Focus Group Discussion Protocol) Additional time will be needed before enrollment for training staff and interviewers. There will be a period to pilot test the consent and questionnaires. There will be a gradual roll-in period with enrollment of a few participants per week initially. This will help further test the instruments, evaluate staff and identify as well as correct weak points. A brief evaluation will be done after the first 20 have been enrolled. Amendments to the protocol will be made at that time if necessary, before continuing with any enrollment of study participants.

10. ETHICAL CONSIDERATIONS

In the development of this phase II open label protocol, we have relied heavily on the International Ethical Guidelines for Biomedical Research Involving Human Subjects (CIOMS) with respect to its guidance concerning the basic principles of biomedical research, informed consent, special considerations of pregnant or nursing women, and obligations of sponsoring and host countries.

With respect to the basic principles of biomedical research outlined in the CIOMS document, we have carefully considered the current research on perinatal HIV transmission as well as the ongoing planning by international agencies, and ministries of health to make antiretroviral treatment more widely available in resource-limited settings.

At present most HIV-infected women in resource-limited settings choose to breast feed, and there are no interventions known to reduce their risk of transmitting HIV to their infant through breastfeeding. This

study utilizes highly active maternal antiretrovirals widely used in the US and available in Kenya to try to reduce the risk of transmission during the first 4-6 months of lactation, followed by infant weaning. The protocol addresses the interface of perinatal HIV prevention with adult antiretroviral treatment and focuses both on the effectiveness of the intervention in reducing transmission during lactation, and also on careful assessment of the safety and tolerance of combination antiretroviral drugs given to pregnant and breastfeeding HIV-infected women volunteers in western Kenya.

The foreseeable benefits of the study far outweigh the known predictable risks of giving antiretrovirals to the participants. If successful, the intervention should help advance perinatal HIV prevention research efforts in reducing the risk of breastfeeding HIV transmission for those HIV-infected women who opt to breastfeed. It should also guide public health policies on both treatment and prevention in resource-limited settings. Serious efforts are currently underway to expand the PMCT program in Kisumu to provide ARVs to pregnant women with severe disease through the PMCT Plus initiative. Findings from this study will help guide this program and thus benefit the community that participates in the study. If found to be feasible and effective, results from the study will also help guide local and international GAP PMCT programs.

The confidentiality of the study participants will be carefully maintained; staff will be trained regarding the importance of maintaining patient confidentiality. This will be monitored and reinforced by the study PI and Co-PI throughout the study. Only staff directly involved in the women and infant’s care will be aware of the mother’s HIV status. Staff will advise, assist and encourage women who may wish to disclose their status to family members. Women who choose not to disclose their HIV status to family members will be supported in their decision. Approaches to addressing questions from family members concerning medication usage will be discussed.

Informed consent will be obtained after explanation of the study aims and methods, risks and hazards, benefits, and the patient's right to freely withdraw from the study at any time without penalty as outlined in section 6.0 of the protocol and addressed in the consent document. Women's understanding of the study and the consent written in their language will be assessed by a check list of questions after the study has been explained to them in their own language [see Appendix VII (vi)]. Pregnant women under the age of 18 will be allowed to participate in the study if there is consent from the pregnant woman and a parent or legal guardian.

Risk-Benefit assessment*:*

Benefit to Infant:

Mother-to-Child-Transmission of HIV has reversed the gains in infant and child survival achieved over the last two decades through infant immunization and oral rehydration programs. Current perinatal HIV prevention interventions available in resource poor countries where breast-feeding is predominant, achieve less than 50% efficacy so there is a need for more effective interventions. It is anticipated that the HAART regimen given during the antepartum, intrapartum and for 6 months postpartum while breastfeeding will substantially reduce the viral load of the mothers and therefore reduce the risk of HIV transmission to the infant. HIV-positive infants will benefit from access to ARVs for care. Infants who test positive during the study would be treated according to UNAIDS guidelines and followed till the end of the study through age 24 months and then be referred to services through PMCT-Plus. This program provides ARVs for women and members of their family who meet criteria for treatment. Funding has been approved to offer this program through the Provincial Hospital. Kenya is also one of the countries that receives substantial funding through President Bush's International Mother and Child HIV Prevention Initiative and the related Presidential Emergency Plan for AIDS Relief (PEPFAR) . Part of this initiative includes provision of combination antiretroviral therapy to women and infants who meet WHO criteria for treatment. As infants and women complete the KiBS study, those who meet treatment

criteria, will be referred for treatment to the GAP Care program. If at the time the trial is completed treatment programs were no longer available through PMCT-Plus, the PEPFAR/GAP Care program, the study clinic would continue to provide ARVs to infants and women who met criteria during the study until referral treatment programs again became available

Risks to Infant:

In pediatric patients receiving NVP the most common adverse events were similar to those seen in adults, i.e. rash, anemia and abnormal liver function, with the exception that granulocytopenia was more common in children. ZDV, 3TC, and NVP are detected in breast milk at concentrations that are 10-20 times lower than pediatric therapeutic levels[53]. The potential toxicity in exposed infants is therefore expected to be very small. Clinical and laboratory evaluations will be performed on the infant to monitor for the common adverse events during the period that the mother is on medication and breastfeeding.

Benefits to Mother:

The benefits of 7 months of antiretrovirals followed by interruption are unknown but would most likely depend on the level of immuno-deficiency of the woman. For women with CD4 count $>500/mm^3$, the maternal health benefits are probably minimal, other than the anticipated reduced risk of having an HIV infected baby.

Those that meet the current UNAIDS criteria for treatment at any point in the study (either with CD4 count $<200\ mm^3$, or with clinical symptoms of AIDS and CD4 $<350\ mm^3$) will benefit from access to ARVs for care. At these stages of HIV infection the health benefits far outweigh the risks related to drug toxicity. The chosen regimens are currently being used for treatment in countries where these drugs are easily available. Women who meet treatment criteria during the study would be treated and followed till the end of the study and then referred for longer-term treatment through the PMCT-Plus. If no program becomes available either through PMCT-Plus or the President Bush Initiative, the study clinic will

continue to provide ARVs to women who met criteria during the study until such programs become available.

Risk to the Mother:

Regardless of CD4 count the potential treatment risks include drug side effects, emergence of resistance through poor adherence and social discrimination through participating in the study. The most common adverse effects due to drugs expected for the mother taking the study regimen include rash, anemia and abnormal liver function. The combination ZDV/3TC has an excellent safety profile in non-malarial areas. In western Kenya where malaria is endemic, the background rates of anemia are high. Women will be excluded if they have hemoglobin less than 7gm/dl until resolved and study participants will be monitored closely for anemia. The most common side effects for NLF are gastrointestinal including diarrhea; and most symptoms are mild. The most common adverse reactions to NVP include rash and hepatic toxicity. Careful clinical and laboratory evaluations will be maintained throughout the intervention period to monitor for abnormal liver function and study staff will be specifically trained on detection of rash. Women will also be given information on how to recognize these side effects and advised to return to the study clinic or seek medical attention at any time if they feel there is a problem.

Women in the study will be followed at frequent set intervals in the clinic to monitor them while on the combination antiretrovirals, to assess safety and to counsel them repeatedly on adherence to the regimen in order to minimize the emergence of resistance. Monitoring for decline in clinical status and decline in CD4 counts will continue throughout the study period; treatment regimens will be altered in the event of apparent treatment failure, toxicity or intolerance.

In order to reduce social discrimination, women will be counseled to share their serostatus with other close family members. Focus group discussions will be held with the women to determine the

acceptability of home visits and whom they would feel most appropriate to do home visits. Efforts will be made with the staff and the community to reduce the stigmatization of this disease. The ability of women to continue breastfeeding instead of artificial feeding to reduce mother to child transmission will help reduce the visibility of the intervention. Additionally, the PMCT program evaluation cohort will be taking place at the same time and will involve the same follow-up efforts and staff as the HAART cohort. The PMCT study design includes HIV-uninfected women. The community will be aware that simply because a woman has a home visitor following up on her and her child does not mean that she is HIV-infected.

For most women in the study, the ARVs will be stopped after 6 months postpartum. The impact of treatment interruption for these women is expected to be similar to the impact of Structured Treatment Interruption in US studies where transient viral rebound to levels above pretreatment have been reported. This could theoretically increase the risk of transmission to the infant if the mother does not wean as counseled and also to the sexual partner. Women will be counseled to strictly adhere to the regimen and avoid interrupting the intervention prior to weaning. They will be advised not to breastfeed after the end of the intervention (6 months postpartum) and condoms will be promoted and provided to prevent transmission to her partner (s). The effects of 7 months of therapy on future clinical, immune and virologic status will be determined at the end of the study, one year after the HAART intervention.

Justice Considerations. This research will be giving short term highly active antiretrovirals to pregnant and nursing women volunteers in the study; at a time when there is only very limited availability of ARVs for treatment in the Kisumu area. The justification for this approach is based on the urgent and compelling need to test strategies which could substantially reduce the risk of HIV transmission through breastfeeding for the majority of HIV-infected women who opt to breast feed. Also the choice of pregnant women and mothers post partum is consistent with prioritization of maternal-child health which acknowledges the importance of mothers as the primary care providers in families, and that the death of a

mother is known to be the major risk factor for death of the infant. Thus in many settings women are considered one of the high priority groups to first receive interventions. As a recent example, endorsed by various donor groups, the PMCT-Plus initiative builds on this philosophic approach. There is also the importance of including pregnant and lactating women in a careful trial design to thoroughly assess and ensure the safety of these combination antiretrovirals before they are used more widely in provincial and national treatment programs and given to HIV-infected women of child bearing age in resource limited settings.

As mentioned above serious efforts are being made to establish a program in this community that will provide ARVs to pregnant women with advanced disease. There are also some treatment options available to others in settings close to Kisumu, and it is anticipated that other programs will be started up in the Kisumu area within the time of the study.

Distributive Justice: The government of Kenya is committed to obtaining ARVs for treatment and for PMCT. It has submitted an application to the Global Fund for AIDS, most of which is to be used to purchase ARVs and to provide mechanisms for treatment. A pilot treatment program is currently underway at the Coast Provincial Hospital in Mombasa. In addition the Ministry of Health frequently reviews its PMCT policies. If this study regimen is found to be more effective than the current national PMCT program regimen using NVP, the government would seriously consider adopting the new regimen for PMCT at the national, provincial and local level in Kisumu (See Appendix XII for letters from the Head of the National AIDS and STD Control Program [NASCOP] and the Provincial Medical Officer of Nyanza Province). As mentioned above serious efforts are also being made to establish a program in this community that will provide ARVs to pregnant women with advanced disease through the PMCT-Plus initiative.

Concerns regarding coercion: There are ethical concerns that HIV-infected women might feel undue coercion to breast-feed and to take part in this trial particularly if entry into the trial were their only means of securing antiretroviral drugs. We believe that undue coercion will not be a factor in a woman's decision to participate in this trial for several reasons. First, the decision to breastfeed will have been made before the study is introduced to the woman. The HIV-infected women who will be approached for this study receive counseling on infant feeding according to UNAIDS guidelines. Almost all choose to breast feed in this setting based on an unsafe water supply and cultural norms. Second, other treatment options are likely to become available in Kisumu during the study period as a result of the PMCT Plus initiative and President Bush's new initiative – both of which will provide antiretrovirals to the small subgroup of HIV-infected pregnant women who require them based on WHO/UNAIDS criteria. Several care and treatment programs have been initiated in and around Kisumu within the last 2 years and ARVs are being given at little or no cost to the patients.

It is our plan to communicate clearly to women considering volunteering for this study that the drugs in the trial are not for treatment (unless the mother's immune status necessitates treatment for her own health) but for prevention of transmission to the child and that they will only receive drug for a limited time period during the trial for the purposes of testing a perinatal intervention. If they were to meet criteria for initiation of ARV treatment, however, we would provide for their care for the duration of the study and then refer them in programs that we anticipate will be in place following their participation in the study.

In agreeing to take part in a clinical trial these women as volunteers are accepting the extra burden and time commitment of being in a trial including the risk of known predictable adverse reactions to the antiretrovirals used in the trial. The situation is somewhat analogous to early antiretroviral treatment trials in the U.S. where only a small group received new drugs during the study, but where the anticipation was that wider access would be available within a reasonable time frame if the drug regimen

proved efficacious. Based on all of the above discussion, we believe that the women who agree to take part in this study will have done so without undue influence or coercion and the alternative of not doing the research would a detriment for the community and public health.

Protection of human subjects

Institutional Review and Informed Consent

This protocol, the informed consent document (Appendix) and any subsequent modifications will be reviewed and approved by the Institutional Review Boards or Ethical Review Committees responsible for oversight of the study. Written informed consent will be obtained from the subject (or parent or legal guardian of subjects who are minors and cannot consent for themselves, e.g., mothers between 15 and 18 years and infants). The informed consent will describe the purpose of the study, the procedures to be followed, and the risks and benefits of participation. A copy of the consent form will be given to the subject (or parent or legal guardian) if she is willing to receive it.

Subject Confidentiality

All laboratory specimens, reports, study data collection, process, and administrative forms will be identified by a coded number only, to maintain participant confidentiality. All records that contain names or other personal identifiers, such as locator forms and informed consent forms, will be stored separately from study records. All local databases must be secured with password-protected access systems. Forms, lists, logbooks, appointment books, and any listings that link participant ID numbers to other identifying information must be stored in a separate, locked file in an area with limited access.

Clinical information will not be released without the written permission of the participant, except as necessary for monitoring by the Food and Drug Administration, the Centers for Disease Control and Prevention, and/or the Kenyan Ministry of Health.

Study Discontinuation

The study may be discontinued at any time by CDC, the Kenyan government, or by the Protocol Team upon the recommendation of the Safety Review Group or the IRBs.

Dissemination of Results

Feedback, updates, and study results will be presented to the study participants and the communities from which they come after the study ends and before publication of the findings. Interim updates and feedback to the community will be given as needed or requested by the community.

Anticipated outcomes/interventions from study results

If this trial demonstrates safety and efficacy of HAART as an intervention during lactation to safely prevent HIV transmission from mother to child, it will change policy in resource-poor countries and be an intervention that will allow neonates and infants the benefits of being breastfed with a much-reduced risk of HIV-infection through breast milk. Positive results from this trial can also guide HIV care in resource-poor countries and within the community of Kisumu, with respect to monitoring of the safety and toxicity of HAART and anticipating potential obstacles to adherence. The trial will also provide data on the relatively safety profile of NVP vs NLF among HIV infected pregnant and post partum women in Western Kenya which should help inform the degree of monitoring that would be needed in programmatic use.

11. EXPECTED APPLICATIONS OF THE RESULTS

If the combination maternal ZDV/3TC and NVP regimen is shown to be highly effective in preventing mother-to-child transmission of HIV both during the peripartum and lactation periods, it is anticipated that the regimen would likely be supported and subsidized as part of planned treatment initiatives being coordinated by international agencies. In addition, continuing ARVs for the subset of women requiring treatment for their own health after the period of the study, should serve as a useful model for further expansion of antiretroviral treatment to HIV-affected families. Maintaining better health and prolonging survival for HIV-infected mothers will result in improved infant survival, as loss of a mother is a major risk factor for infant and child mortality.

The proposed research study interfaces well with the GAP initiative. The information gathered on ARV adherence and monitoring will be useful for guiding GAP programs involving HIV/AIDS care. The presence of CDC's ongoing GAP activities and the CDC/KEMRI Program in Kisumu, Kenya, both with a long-standing relationship with the Provincial General Hospital, make CDC the ideal organization to conduct this study.

Publication of the results of this trial will be governed by CD/KEMRI and CDC-Atlanta publication policies. Any presentation, abstract, or manuscript will be submitted to CDC/KEMRI in Kenya and CDC-Atlanta for clearance prior to submission for journal publication or presentation at a conference; and to the drug company sponsors.

12. REFERENCES

1. De Cock KM, Fowler, Mercier E, et al. Prevention of mother-to-child HIV transmission in resource-poor countries: Translating research into policy and practice. JAMA 2000; 283 (9): 1175-1182.

2. The Working Group on Mother-to-Child Transmission of HIV. Rates of mother-to-child transmission of HIV-1 in Africa, America and Europe: results from 13 perinatal studies. J Acquir Immune Defic Syndr Hum Retrovirol 1995;8: 506-10.

3. Bulterys M, Fowler MG. Prevention of HIV infection in children. Pediatr Clin North Am 2000; 47: 241-260.

4. Fowler MG, Simonds RJ, Roongpisuthipong A. Update on perinatal HIV Transmission. Pediatr Clin North Am 2000; 47: 21-38.

5. Joint United Nations Programme on HIV/AIDS. World Health Organization. Report on the global HIV/AIDS epidemic. December 2003.

6. DeMartino M, Tovo PA, Tozzi AE, et al. HIV-1 transmission through breast milk; appraisal of risk according to duration of feeding. AIDS 1992; 6: 991-997.

7. Datta P, Embree JE, Kreiss JK, et al. Mother-to-child transmission through of human immunodeficiency virus type 1: Report from the Nairobi Study. J. Infect Dis 1994; 170: 1134-1140.

8. Bertolli J, St. Louis ME, Simonds RJ et al. Estimating the timing of mother–to-child transmission of human immunodeficiency virus in breast-feeding populations in Kinshasa, Zaire. J Infect Dis 1996; 174 772-776.

9. Ekpini ER, Wiktor SZ, Satten GA, et al. Late postnatal mother-to-child transmission of HIV-1 Abidjan, Cote d'Ivoire. Lancet 1997;349: 1054-1085.

10. Connor EM, Sperling RS, Gelber R, et al. Reduction of maternal-infant transmission of human immunodeficiency virus type 1 with zidovudine treatment. Pediatric AIDS Clinical Trails Group Protocol 076 Study Group. N Engl J Med 1994; 331: 1173-1180.

11. Shaffer N, Chuachoowong R, Mock PA, et al. Short-course zidovudine for perinatal HIV-1 transmission in Bangkok, Thailand: a randomized controlled trial. Lancet 1999; 353: 773-780.

12. Leroy V, Karon JM, Alioum A, et al. Twenty-four month efficacy of a maternal short-course zidovudine regimen to prevent mother-to-child transmission of HIV-1 in West Africa. AIDS 2002; 16: 631-641.

13. Lange J for the PETRA team. The PETRA study: early and late efficacy of 3 short ZDV/3TC combination regimens to prevent MTCT of HIV-1. Presentation, Third Conference on Global Strategies for the Prevention of HIV Transmission from Mothers to Infants. 2001, Kampala.

14. Guay LA, Musoke P, Fleming T, et al. Intrapartum and neonatal single-dose nevirapine compared with zidovudine for prevention of mother-to-child transmission of HIV-1 in Kampala, Uganda. HIVNET 012 randomised trial. Lancet 1999; 354: 795-802.

15. Owor M, Deseyve M, Ducar C, et al. The one year safety and efficacy data of the HIVNET 012 trial. 13th int AIDS Conference, Durban, July 2000 (Ab LB Or1).

16. Leroy V, Newell ML, Dabis F, et al. International multicentre pooled analysis of late postnatal mother-to-child transmission of HIV-1 infection. Lancet 1998; 352; 597-600.

17. Nduati R, John G, Mbori-Ngacha D, et al. Effect of breastfeeding and formula feeding on transmission of HIV-1. JAMA 2000; 283:1167-1174.

18. Moodley D, on behalf of the SAINT Investigators Team. The SAINT trial: nevirapine versus zidovudine + lamivudine in prevention of peripartum HIV transmission. 13th In AIDS Conference, Durban (ab LBOr2), 2000.

19. Miotti PG, Taha T, Kimwenda NI, et al. HIV transmission from breastfeeding. A study in Malawi. JAMA 1999; 282: 744-749.

20. World Health Organization. A manual for the treatment of acute diarrhea. WHO/CDD Series/80. 2 Geneva: WHO 1984.

21. Coutsoudis A, Pillay K, Kuhn L, et al. Method of feeding and transmission of HIV-1 from mothers to children by 15 months of age: prospective cohort study from Durban, South Africa. AIDS 2001; 15: 379-387. AIDS 2001; 15;379-387.

22. Joint United Nations Program on HIV/AIDS. HIV and infant feeding. Wkly Epidemiolog Rec 1996; 71: 289-91.

23. WHO-UNAIDS-UNICEF Technical consultation of HIV and Infant Feeding. Geneva April 20-22, 1998. Conclusions.

24. World Health Organization. Technical consultation on behalf of the UNFPA/UNICEF/WHO/UNAIDS Interagency Task Team on Mother–to-child Transmission of HIV. New data on the prevention of mother to child transmission of HIV and their policy implications. Conclusions and recommendations. Geneva, Switzerland: World Health Organization; October 11-23, 2000.

25. Mofenson L, Munderi P. Safety of antiretroviral prophylaxis of perinatal transmission on HIV-infected pregnant women and their infant. Background paper prepared for Technical Consultation on New Data on the Prevention of Mother to Child transmission of HIV and their policy implications. Geneva, 11-13 Oct. 2000.

26. Mandelbrot L, Landreau-Mascaro A, Rekacewicz C, et al. Lamivudine-zidovudine combination fro prevention of maternal-infant transmission of HIV-1. JAMA 2001; 285;2083-2093.

27. Dorenbaum A. Report of results of PACTG 316: an international phase III trial of standard antiretroviral prophylaxis plus nevirapine for prevention of perinatal HIV transmission. Presented at the 8th Conference on Retroviruses and Opportunistic Infections. Chicago, Feb 2001 (Abstract LB7).

28. Investigator's Brochure: Nevrapine (BI-RG-587). HIV-1 reverse transcriptase inhibitor for use in moderate to severe HIV-1 related disease. Boehringer Ingelheim Pharmaceuticals, Inc. January 11, 2005.

29. FDA Public Health Advisory for Nevirapine (Viramune). January 19, 2005.

30. Mirochnick M, Fenton T, Gagnier P, et al. Pharmacokinetics of nevirapine in human immunodeficiency virus type 1 infected pregnant women and their neonates. J Infect Dis 1998;178: 368-74.

31. Cheeseman SH, Havlir D, McLaughlin M, et al. Phase I/II evaluation of nevirapine alone and in combination with zidovudine for infection with human immunodeficiency virus. JAIDS 1994;141-41.

32. Bardsley-Elliot A, Perry CM. Nevirapine: a review of its use in the prevention and treatment of paediatric HIV infection. Paediatr Drugs 2000; 39:281-93.

33. Luzuriaga K, Bryson Y, McSherry G, et al. Pharmacokinetics, Safety, and Activity of Nevirapine in HIV-1 infected Children. J Infect Dis 1996; 174; 713-721.

34. Burchett SK, Carey V, Yong F, et al. Virologic activity of didanosine (ddI) zidovudine (ZDV) and nevirapine (NVP) in pediatric patients with advanced HIV disease (PACTG 245). 5th Conference on Retroviruses and Opportunistic Infections 1998; Feb 1-5 Chicago.

35. Stek A, Jasuja S, Fassett M. et al. A review of nevirapine use in 99 pregnancies. The 3rd conference on Global Strategies for the Prevention of HIV Transmission from Mothers to Infants. Kampala Sept 9-13, 2001, poster 212a, p42.

36. Thomas T, Amornkul, Mwidau J, et al . Preliminary findings : Incidence of serious adverse events attributed to Nevirapine among women enrolled in an ongoing trial using HAART to prevent mother -to-child transmission. 12th Conference on Retroviruses and Opportunistic Infections 2005; Feb 22-25, Boston. Abstract 809, p. 364.

37. Palombi L, Germano P, Liotta G et al. HAART in pregnancy : Safety, effectiveness, and protection from viral resistance: Results from the DREAM Cohort. 12th Conference on Retroviruses and Opportunistic Infections 2005; Feb 22-25, Boston. Abstract 67, p. 90.

38. Phanuphak N, Apornpong T, Intarasuk S, et al. Toxicities from Nevirapine in HIV-infected males and females including pregnant females with various CD4 cell counts. 12th Conference on Retroviruses and Opportunistic Infections 2005; Feb 22-25, Boston. Abstract 21, p. 72.

39. Musoke P, Guay L, Bagenda D, et al. A phase I/II study of the safety and pharmacokinetics of nevirapine in HIV-1 infected pregnant women and their neonates. JID 1998; 178: 368-374.

40. Havlir D, Cheeseman SH, McLaughlin M, et al. High dose NVP: safety, pharmacokinetics and antiviral effect in patients with human immunodeficiency virus infection. J Infect Dis; 171: 534k-45, 1995.

41. Eshleman SH, Mracna M, Guay LA, et al. Selection and fading of resistance mutations in women and infants receiving nevirapine to prevent HIV-1 vertical transmission (HINVET 012). AIDS 2001; 14: 1951-1957.

42. Cunningham CK, Britto P, Gelber R, et al. Genotypic resistance analysis in women participating in PACTG 316 with HIV-1 RNA>400 copies/ml. 8th Conference on Retroviruses and Opportunistic Infections, Chicago, IL, Feb 2001 (Abstract 712).

43. Chaaowanachan T, Chotpitayasunondh T, Vanprapar N, et al. Resistance mutations following a single-dose intrapartum administration of nevirapine to HIV-infected Thai women and their infants receiving short-course zidovudine. 10th Conference on Retroviruses and Opportunistic Infections 2003; Feb 10-14, abstract 855.

44. Jourdain G, Ngo-Giang-Huong N, Le Coeur S, et al. Intrapartum exposure to nevirapine and subsequent maternal responses to nevirapine-based antiretroviral theapy. N Engl J Med, 2004; 351: 220-40.

45. Chaix ML, Dabis F. Ekeouvi D, et al. Addition of 3 days of ZDV+3TC postpartum to a short course of ZDV+3TC and single dose NVP provides low rate of NVP resistance mutations and high efficacy in preventing peripartum HIV-1 transmission : ANRS DITRAME Plus, Abidjan, Cote d'Ivoire. 12th Conference on Retroviruses and Opportunistic Infections 2005; Feb 22-25, Boston. Abstract 72LB, p. 92.

46. Investigator's Brochure: Nelfinavir mesylate (Viracept). Inhibitor of the human immunodeficiency virus (HIV-1) protease. For use in moderate to severe HIV-1 related disease. Agouron Pharmaceuticals, Inc. April 2004.

47. Bryson Y, Stek A, Mirochnick M, et al. For the PACTG 353 team. Pharmacokinetics, antiviral activity and safety of nelfinavir (NFV) in combination with ZDV/3TC in pregnant HIV-infected women and their infants : PACTG 353 Cohort 2. 9th Conference on Retroviruses and Opportunistic Infections 2005; Feb 24-28, Seattle, . Abstract 795-W.

48. Hitti J, Frenkel LM, Stek AM, et al. Maternal toxicity with continuous Nevirapine in Pregnancy. Results from PACTG 1022. J Acquir Immune Defic Syndr 2004; 36:772-776.

49. John GC, Nduati RW, Mbori-Ngacha DA, et al. Correlates of mother-to-child Human

Immunodeficiency Viru7s Type 1 (HIV-1) Transmission: Association with maternal plasma HIV-1 RNA load, genital HIV-1 DNA shedding, and breast infections. J Infect Dis 2001; 183:

50. Turnbull B. The empirical distribution function with arbitrarily, grouped, censored and truncated data. *Journal of the Royal Statistical Society, Series B* 1976; **38**:290-5

51. Hudgens MG, Satten GA, Longini IM. Non-parametric maximum likelihood estimation for competing risks of survival data subject to interval censoring and truncation. *Biometrics* 2001; **57**:74-80

52. Fleiss, JL. (1981). *Statistical Methods for Rates and Proportions*, 2nd edition. John Wiley and Sons, Inc.: New York.

53. USPHS Perinatal HIV Guidelines Working Group Members. Recommendations for Use of Antiretroviral Drugs in Pregnant HIV-1-Infected Women for Maternal Health and Interventions to reduce perinatal HIV-1 Transmission in the United States. www.hivatis.org Feb, 2005)

Appendices

Kisumu Breastfeeding Study (KiBS)
Key Facts Sheet
(English)

What is it?

A research study by CDC, KEMRI, and MOH that gives 3 drugs: Zidovudine/Lamivudine and Nevirapine or Nefinavir (ZDV and 3TC, and either NVP or NLF) to pregnant women in the last month of pregnancy and for up to 6 months after the baby is born. This study will also give one dose of NVP to babies within 7 days after they are born. If you and your baby join, you'll be in the study until your baby is 24 months old.

Who can be in the study?

Women who:
- Have HIV
- Are in their last month of pregnancy
- Are planning to breastfeed their new baby

Why is the study being done?

To look at four things:
- if the 3 drugs will lower the risk of passing HIV from infected mothers to their babies
- if the drugs are safe for pregnant women and women who breastfeed
- if the drugs in the mother's breast milk are safe for the baby
- how many babies when they are 24 months old will not have HIV

What happens during the study?

If you join this study, we will ask you to:
- Only give your baby breast milk until it is 6 months old. Do not give the baby anything else to eat or drink.
- Attend a group that will give you ideas about how to only feed your baby breast milk.
- Take pills two times each day. You will do this till you deliver, and for up to 6 months while you breastfeed.
- Allow us to draw a small amount of your blood and your baby's blood at certain times.
- Allow someone from the study to go home with you and draw a map to your house.
- Allow someone from the study to come to your house to check on you and your baby.
- Tell a study nurse or doctor before taking any drugs or herbals that are not part of the study.
- Tell a study nurse or doctor before joining other studies.

Why should you only feed your baby breast milk until it is 6 months old?
- Studies show that it lowers the risk of your baby getting sick from diarrhea and other illnesses.
- There may be a lower risk of the baby getting HIV.

What do you get for being in the study?
- You and your baby may or may not benefit directly from this study.
- But, taking all 3 of the study drugs for a short amount of time may decrease the risk of passing HIV to your baby.
- All study drugs, study clinic visits, physical exams, transport to study clinic visits, lab tests for the study, and your hospital delivery fees will be provided by the study.

- Neither you nor your baby will be paid for being in this study. But, you will receive money to pay for your transport.
- You and your baby may help us find ways to stop babies from getting HIV from breast milk. This may help other mothers and babies in Kenya in the future.

What bad things can happen to you and your baby if you join the study?

- The drugs may make you sick. They may cause you to have a skin rash, fever, nausea, headaches, sleeplessness, muscle aches, and liver problems. They may cause you to have low blood (anemia).
- The study drugs may make your baby sick. He/she may have a skin rash, fever, nausea, headache, and liver problems. It could also cause your baby to have low blood (anemia).
- When we draw your blood and your baby's blood, there may be some pain, bleeding, bruising, swelling, or infection where the needle goes through the skin.

When can you leave the study?

- You can leave the study at any time. If you want to leave the study, just tell a study nurse or doctor.

Kisumu Breastfeeding Study

Scheduled Visits For Mother

Clinic visits	Counseling and Questions	Exam	Blood	Home visits	Comments
**Recruitment 26-32 weeks**	X				Assess eligibility
**CRC Screening 33 weeks**	X	X	X	**Home Location Visit**	Assess eligibility and consent
**Enrollment** 34 weeks	X	X	X		Start medication
**End week 1**	X	X	X	**Week 1**	
**End week 2**	X	X		**Week 2**	
**End week 3**	X	X	X	**Week 3**	
**End week 4**	X	X			
**End week 5**	X	X	X	**Week 5**	
**End week 6**	X	X			
**End week 7**	X	X	X	**Week 7**	
**Delivery**	X	X	X		
**End week 2**	X	X	X	**Week 1**	
**End week 6**	X	X	X	**Week 4**	
**End week 10**	X			**Week 8**	
**End week 14**	X	X	X	**Week 12**	
**End month 5**	X			**Month 4**	Start weanig
**End month 6**	X	X	X	**Month 6**	Stop medicines and breastfeeding
**End month 9**	X	X	X	**Month 7 and 8**	
**End month 12**	X	X	X	**Month 10 and 11**	
**End month 15**				**Month 14**	
**End month 18**	X	X	X	**Month 17**	
**End month 24**	X	X	X	**Month 20 and 22**	

Kisumu Breastfeeding Study

Scheduled Visits For Baby

Clinic visits	Counseling and Questions	Exam	Blood	Home visits	Comments
**Delivery**	X	X	X		Nevirapine
**End week 2**	X	X	X	**Week 1**	
**End week 6**	X	X	X	**Week 4**	Immunizations
**End week 10**	X			**Week 8**	Immunizations
**End week 14**	X	X	X	**Week 12**	Immunizations
**End month 5**	X			**Month 4**	Start weaning
**End month 6**	X	X	X	**Month 6**	Stop medicines and breastfeeding
**End month 9**	X	X	X	**Month 7 and 8**	
**End month 12**	X	X	X	**Month 10 and 11**	
**End month 15**	X			**Month 14**	Immunizations
**End month 18**	X	X	X	**Month 17**	
**End month 24**	X	X	X	**Month 20 and 22**	

MAIN STUDY CONSENT FORM

(Consent 01A)

This form covers the Mother, fetus and infant. It must either be signed by the mother if she is aged 18 or older, OR by a legal guardian of the mother if she is under 18 years of age. If the mother is under 18 years of age she must sign the assent form.

Flesch-Kincaid Score: 6.7

TITLE OF STUDY: Kisumu Breastfeeding Study (KiBS)

INFORMED CONSENT
Because you are pregnant and have the Human Immunodeficiency Virus (HIV), the virus that causes Acquired Immunodeficiency Syndrome (AIDS), you and your baby are being asked to be in a research study. This study is from the U.S. Centers for Disease Control and Prevention (CDC), the Kenya Medical Research Institute (KEMRI), and the Kenya Ministry of Health (MOH). This study is being done with about 520 mother/baby pairs.

This study will give 3 drugs that fight HIV to infected women in the last month of pregnancy and for 6 months while they breastfeed their infants. The study will look at three things:
(1) the effects of the drugs on the mother;
(2) the effects of the drugs on the baby;
(3) if the drugs will stop an infected mother from passing the virus to her baby.

Before you decide if you wish to be in this study, you need to know about any good or bad things that could happen if you decide to join. This form tells you about the study. You can ask any questions you have at any time.

BEING IN THE STUDY IS YOUR CHOICE
This consent form tells you about the study. This information will also be discussed with you. Once you understand and decide that you and your baby should take part in this study, you will be asked to sign this consent form. We will also give you a copy to keep.

You are here because you have decided that you will breastfeed your baby. You have been told about good and bad things about breastfeeding. You have also been told that there are other feeding methods and that there are good and bad things about each of them.

Before we tell you more about the study, you should know that:
1. It is your choice to join and allow your baby to join this study.
2. You may decide not to join the study.
3. If you choose to be in the study, you and your baby can withdraw at any time.
- If you choose to be in the study, you don't have to answer any questions that you don't want to.

<u>The goals of this study are:</u>
1. To see if three drugs called zidovudine (ZDV), and lamivudine (3TC) used with either nevirapine (NVP) or nelfinavir (NLF) can lower the risk of your baby getting HIV. You

will take the drugs from the last month you are pregnant to 6 months while you breastfeed.
2. To look at the safety of these drugs for pregnant women and women who breastfeed. The study will see if the women are able to take the drugs. It will see what side effects they have.
3. To look at the safety of these drugs for the baby while it is breastfed. The study will see what side effects these drugs have on the baby.
4. To see how many babies in the study at 24 months don't have HIV.

If you choose to be in the study, you and your baby will be in the study for 24 months after your baby is born.

WHAT HAPPENS DURING THE STUDY

- As soon as you join the study, you will have a physical exam. A member of the study staff will ask you questions about your past health. About 3 teaspoons of blood will be taken from a vein in your arm to do tests. These tests will be done to confirm your HIV status and to tell us if it is safe for you to be in the study. If it is safe for you and your baby to be in the study and you agree to join, you will be in this study from the last 4-6 weeks of your pregnancy until 24 months after your baby is born. Your baby will be in this study for the first 24 months of his/her life. So that we can follow your and your baby's health during this time, you must live in or around Kisumu for the whole time you are in the study.

- During the time in the study we will be doing tests on your blood to make sure it is safe for you to continue to get the study drugs. We will also be monitoring how the drugs are affecting the HIV virus and the effect of the virus on your body. At each visit we will be asking you questions and filling forms. All this information is to help us understand more about the women in the study. All the information will be kept private.

- We would like to send a member of the study team to your home to check on you and your baby from time to time. In order to do this, a study staff member will go home with you and draw a map to your home in Kisumu. They will also ask you other places where you might be staying if you are not at home.

- You will be given ZDV, 3TC and either NVP or NLF to take every day. We will decide if you should get NVP or NLF based on your CD4 count in your blood and tests to see if you have any liver infection. The CD4 count is a test we can do on the blood, which gives us some information about how well a person's body is able to fight infections. HIV infection makes people's CD4 counts go down. If your CD4 count when you join the study is less than 250 cells you will be given NVP unless you have a liver infection in which case you will be given NLF. If your CD4 count when you join the study is equal to or more than 250 we will give you the drug NLF instead of NVP. You will be taking these pills twice a day from the last month that you are pregnant until 6 months after your baby is born. These drugs will lower the amount of HIV in you. They should also lower the chance of your baby getting HIV. NVP will also be given to your baby by mouth one time usually before it is 3 days old (but no later then 7 days after birth). NVP has already been shown to lower the risk of babies getting HIV during pregnancy, delivery, or breastfeeding. It is offered to all mothers with

HIV at the New Nyanza Provincial Hospital. After you deliver, you will still take all three drugs the same way you did before you delivered. You will take these drugs until you stop breastfeeding or until the baby is 6 months old. If you have been taking NVP and the drugs are to be stopped, then the NVP will be stopped at 6 months and the other drugs will be continued for a further 21 days.

- While you are taking the study drugs and breastfeeding, you will need to feed your baby only breast milk. Do not add cereal or water to your baby's diet for the first 6 months. We will ask you to be part of a group that will give you ideas about how to do this.

- These medicines are also commonly used to treat people who have become sick with AIDS. They are not a cure, but do help to decrease the amount of HIV in the body. This helps people feel better and they are able to fight other infections. The decision to put people on these drugs is based on how sick they are and a blood test called a CD4 count. If you get sick with AIDS, you will not be taken off the drugs. You may be asked to take them longer. If you still need to take the drugs after the study ends, you will either continue to get medicines through our clinic or through other programs in Kisumu, which are now being set up.

- You may choose to tell or not to tell the baby's father about being in the study. If you do tell the baby's father and he does not want you and your baby to be in this study, we will help you tell him again about the study and your role in it. If he still does not want you to be in the study, you may choose to leave the study. You should make your choice based on what is best for you and your baby.

Mothers

If you agree to join and allow your baby to join this study:

- While you are pregnant, you will need to come to the clinic every week. Every 2 weeks about 3 teaspoons of blood will be taken. .

- **When you come to the hospital to deliver, you will be examined by a study doctor. About 3 teaspoons of blood will be taken from you while you are in the hospital.**

- If you deliver at home, you will need to come to the clinic within 7 days of delivery. We will examine you at that visit. We also will take 3 teaspoons of blood like we would have done if you delivered at the hospital.

- At 2 weeks, 6 weeks, 14 weeks, 6 months, 9 months, 12 months, 18 months and 24 months after you give birth, we will draw your blood and give you a physical exam. Some of this blood will be stored. The blood will be stored at the clinic or CDC/KEMRI without your name on it. Instead, it will have a study code number on it. Study staff will know which code number belongs to you and your baby. No one else will be able to know that it is your blood.

- You should tell a study nurse or doctor before taking any drugs or herbals that are not part of the study.

- You may be asked to join a breast milk substudy. 150 women will be recruited into that study. If you agree, some blood and breast milk will also be taken at birth, and then 2 weeks, 6 weeks, and 6 months after the birth and kept for future tests. We will collect about 4 teaspoons of breast milk. We will also advise you to stop breastfeeding at 6 months. (See separate consent form). You can agree for you and your baby to only take part in the main study or in both the main study and the breast milk study.

Infants

- After birth, ideally within 2 to 3 days, but within the first 7 days, your baby will be given one dose of liquid NVP.

- At birth, 2 weeks, 6 weeks, 14 weeks, 6 months, 9 months, 12 months, 18 months and 24 months of age, your baby will be examined. We will also draw about a teaspoon of blood from a vein your baby's arm. Some of this blood will be stored at the clinic or CDC/KEMRI. The blood will be stored without your or your baby's name on it. Instead, it will have a study code number on it. . Study staff will know which code number belongs to your baby. No one else will be able to know that it is your baby's blood.

- If we cannot draw your baby's blood from a vein at these visits, we will prick his/her finger or heel to get a small amount of blood.

- We will also test your baby's blood for HIV. We will test your baby for HIV 3 and 6 months after delivery and then at 24 months when you end your time in the study. When we have the test results, we will let you know your baby's HIV test results.

- **If your baby gets sick with AIDS while in the study, we will provide medicines used** to treat people who have become sick with AIDS. They are not a cure, but do help to decrease the amount of HIV in the body. This helps people feel better and they are able to fight other infections. If your baby **gets sick with AIDS while in the study** and still needs to take the drugs after the study ends, he/she will either continue to get medicines through other programs in Kisumu, which are now being set up, or our clinic.

IF YOU CHOOSE TO LEAVE THE STUDY

- You can choose to leave the study at any time. This will not affect the health care you received before you joined the study.

- If you choose to leave this study for any reason before you have your baby, you will be asked to take one dose of NVP when your labor starts. You will also be asked to allow your baby to receive one dose of NVP after birth (between 3 and 7 days). This is what the Kenyan government promotes to protect infants from getting HIV from their mothers.

- If you choose to leave the study and not take even one dose of NVP during labor, you will be asked to allow your child to receive on dose of NVP as soon as possible after birth. This one time dose is to protect your baby from becoming infected with HIV. Your baby would have a lower chance of getting HIV if you take a dose as well.

- You should tell a study nurse or doctor before joining other studies.

RISKS TO YOU
We will decide if you should get NVP or NLF based on your CD4 count and tests to see if you have any liver infection. If your CD4 count when you join the study is less than 250 cells you will be given NVP, unless you have a liver infection in which case you will be given NLF. The most common side effects after many doses of NVP are skin rash, fever, nausea, headache, and blood tests showing a liver that is not normal. 1 out of 6 adult patients have rashes from NVP. Some patients have also had problems in the mouth and eyes. Severe rash happens in about 1 out of 16 patients taking NVP. This type of rash can make patients very ill. If this happens, we will tell you to stop taking NVP.

The most serious skin rash from NVP is called Stevens Johnson Syndrome. It is rare, but people with Stevens-Johnson Syndrome have bumps and blisters on their mouth and eyes. The skin and wet areas of their mouth and eyes may peel off. They may also have a fever, chills, and headache. In severe cases, the kidneys, lungs, and stomach may also have bumps and blisters. Rarely, death has been reported.

We do not know what causes this severe rash but it occurs more often when a woman's CD4 count is higher than 250. We do know that most of the severe rashes happened within the first 42 days (6 weeks) of taking NVP. To lower the chance that you will have a severe rash, we will start you with a lower dose of NVP for the first two weeks.

Severe liver damage has been found in people on chronic (long time) treatment with NVP. It most commonly occurs within the first 18 weeks after a person begins taking the drug. If you have other liver infections such as hepatitis or drink alcohol while taking the study drugs, the risk to your liver is higher. You should not drink alcohol while taking NVP. Very few patients taking NVP have symptoms of liver damage. It has been reported in pregnant women as well. In very rare cases, death due to liver problems has been reported in pregnant women taking NVP. So it is important that we take your blood to check your liver while you are taking NVP.

If your CD4 count when you join the study is equal to or more than 250 we will give you the drug NLF instead of NVP. NLF has been used among pregnant women in the U.S. very often and usually has few side effects. Some women get loose stools, gas or other mild symptoms but most have no problems. Some have had changes in blood sugar and levels of fats in their blood; and some changes to body fat.
Also some have had changes in blood sugar and levels of fats in their blood; and some changes to body fat. Most women tolerate NLF well without problems.

The drugs 3TC and ZDV are safe and very effective when taken at the same time. There is a small chance that 3TC or ZDV may cause damage in the liver or blood such as low blood (anemia) or lower your cells that fight infections. They may also cause headache, sleeplessness, and muscle aches. Many studies in Africa, Europe, and the U.S.A. have shown that 3TC and ZDV are generally safe in pregnant women. We will do blood tests to check for these side effects on you and your baby during the study.

If you decide to enter the study and are found to be eligible, we will tell you what to do in the event that you have one of these side effects.

RISKS TO THE FETUS/YOUR BABY
The most common side effects from NVP in infants and children are similar to those in adults. But some children also develop blood changes that are not common in adults taking NVP. Serious side effects are rare. They include Stevens Johnson Syndrome (described above) and severe allergic reactions. The most common side effect in babies is a mild rash. You should tell your doctor and a study staff member about any rash on your baby as soon as possible.

Studies have shown that early breast milk can have NVP in it. As stated above, a rash can occur when taking NVP. Babies taking milk from mothers on NVP may develop a rash. Again, you should tell your doctor or other study staff about any rash on your baby as soon as possible.

Other studies have shown that there are small amounts of 3TC and ZDV in breast milk. These doses are small, so the chances of the drugs causing low blood (anemia) or other health problems in your baby are very low. We will check your baby's blood to make sure that these drugs are not causing any health problems.

All the drugs; ZDV, 3TC, NVP and NLF have been used a lot during pregnancy. No major problems have been noticed in the babies whose mothers took these drugs while pregnant.

Blood drawing may cause some pain, bleeding, or bruising where the needle goes through the skin. It may also cause some lightheadedness or dizziness. A small blood clot may form where the needle enters the body. Swelling of the skin in the area may occur. There is also a small risk of a minor infection on the skin where the blood is drawn.

BENEFITS TO YOU AND/OR YOUR BABY
Taking NVP for a short amount of time can lower the risk of passing HIV from a pregnant mother to her baby. Taking ZDV and 3TC together for a short amount of time can also lower the risk of passing HIV from an infected mother to her infant. Because taking either ZDV/3TC and NVP or NLF for a short amount of time can decrease the risk of passing HIV to your baby, using these drugs for longer amounts of time should work in protecting your baby from HIV.

In studies from the US, few mothers with HIV passed the virus to their babies when they were taking all 3 drugs. But these studies were from mothers who did not breastfeed. Your baby should do very well if he or she is able to take the drug and not have bad side effects.

You and your baby still may or may not benefit directly from this study. But what we learn may be useful in finding a treatment to prevent other HIV-infected mothers from passing the virus to their babies.

NEW FINDINGS
You will be told of any new findings learned during the study. These findings may cause you to change your mind about you and your baby staying in the study. At the end of the study, you will be told when study results may be known and how to learn about them.

REASONS FOR TAKING YOU OUT OF THE STUDY WITHOUT YOUR CONSENT
You and your baby may be taken out of the study without your consent if:

- Your doctor or your baby's doctor decides that staying in the study may cause harm to you and/or your baby;
- Your baby is born with health problems that might cause him/her to die;
- You or your baby need(s) a treatment not allowed on this study;
- You are unable to keep clinic visits;
- You are unable to take the study drugs as told;
- The study is stopped by the CDC, KEMRI, or MOH;
- Other reasons beyond our control.

You will not be given or not allowed to keep taking ZDV, 3TC, NVP or NLF if:

- Before delivery, you have high blood pressure, severe headache, too much protein in your urine, and severe swelling of the legs and feet.
- During labor, you have seizures.
- During labor, you have problems that must be treated with drugs that stop your blood from clotting.
- You have a severe infection that may cause shock.
- After delivery, you have high blood pressure that does not get better with treatment.
- You cannot take pills by mouth.
- Your blood test shows that your liver is not normal or that you have severe low blood (anemia).

Your baby will not be given NVP if he/she has serious health problems and:

- Cannot take the study drug by mouth.
- His/her blood test shows that the liver is not normal.

COSTS TO YOU

- There is no cost to you or your baby for the study drugs, study clinic visits, physical exams, transport to study clinic visits, lab tests for the study, or for your delivery at the hospital.

- Medical care for common medical problems will be given to you and your baby at the study clinic at no cost to you. If you have to stay overnight at the New Nyanza Provincial General Hospital for health reasons, CDC/KEMRI will pay for the services.

- The health care described above will be given as long as you are in the study.

- Neither you nor your baby will be paid for being in this study.

YOUR RECORDS WILL BE PRIVATE

You and/or your baby's research records will be kept private as allowed by the law. You and your baby will be given code numbers, and any private information from your and your baby's records will not be given out unless you agree in writing. You and your baby's names will not be in any reports about this study. But, people who review the study for KEMRI and CDC may need to look at your and your baby's records from time to time. In order to give you or your baby good medical care and for study needs, we will need to make copies of your medical records from Provincial Hospital and other places where you may get medical care. All copies will be kept in a locked file cabinet. Only study staff will have access to this cabinet.

INJURY BECAUSE OF BEING IN THE STUDY

If you and/or your baby have side effects from the drugs or something bad happens because of being in this study, CDC/KEMRI will find treatment for you and/or your baby as soon as possible. CDC/KEMRI will pay for this treatment.

When you sign this consent form, you do not give up your rights to care for injury caused by being in the study.

PICTURES

We will ask to take your picture. This will help us make sure that no one else can get the medication that is meant for you. It will also help us find you in case of emergency. This picture will not have your name on it, only your study identification number. It will be kept in a secure place with your records. At the end of the study the picture will be given to you if you want it, or it will be destroyed. If you do not want your picture taken, we will not take it. If you chose not to have your picture taken, it will not affect your ability to take part in the study in any way.

- ☐ I give consent to have my picture taken.
- ☐ I DO NOT give consent to have my picture taken.

PROBLEMS OR QUESTIONS

If you have questions or concerns about this study, contact:

- Dr. Timothy Thomas at the CDC/KEMRI study clinic at New Nyanza Provincial General Hospital on Kakamega Road, Kisumu (P.O. Box 1578, Kisumu) or phone number: 057-2023200; or

- Dr. Wairimu Chege of CDC/KEMRI Kisian Office off Kisumu-Busia Road (P.O. Box 1578, Kisumu) or phone: 0572022983/2022959/2022902.

If you have any questions, including about a research subject's rights, you should talk to Margaret Odhiambo at the Provincial General Hospital or phone: 057 44278.

STATEMENT OF CONSENT:

Please sign your name below once you:

1. have read (have been explained) and know the reasons for the study,
2. know the steps to be followed in the study,
3. know the risks and benefits to you and your baby from being in the study,
4. AND choose to enroll you and your baby in this study of your own free will.

**Volunteer's Name**<br>Type or print	*Volunteer's Signature*<br>(Volunteer does **NOT** sign here if she is under age 18)	*Date*
**Volunteer's Legal Guardian or Representative**<br>(if <18 years old)<br>*Type or print*	*Legal Guardian's Signature*	*Date*
**Witness's Name**<br>Type or print	*Witness's Signature*	*Date*

I have explained the purpose of this study to the volunteer. To the best of my knowledge, she understands the purpose, procedures, risks and benefits of this study.

**Investigator/Designee Name**<br>***Type or print***	*Investigator/Designee Signature*	**Date**

NOTE: This consent form with original signatures must be retained on file by the principal investigator. A copy must be given to the volunteer. A copy should be placed in the volunteer's medical record (if applicable).

MAIN STUDY ASSENT FORM
(Assent 02A)

This form covers the Mother, fetus and infant. It must be signed by the mother if she is under 18 years of age. A legal guardian of the mother must sign the consent form.

Flesch-Kincaid Score: 6.7

TITLE OF STUDY: Kisumu Breastfeeding Study (KiBS)

INFORMED ASSENT
Because you are pregnant and have the Human Immunodeficiency Virus (HIV), the virus that causes Acquired Immunodeficiency Syndrome (AIDS), you and your baby are being asked to be in a research study. This study is from the U.S. Centers for Disease Control and Prevention (CDC), the Kenya Medical Research Institute (KEMRI), and the Kenya Ministry of Health (MOH). This study is being done with about 520 mother/baby pairs.

This study will give 3 drugs that fight HIV to infected women in the last month of pregnancy and for 6 months while they breastfeed their infants. The study will look at three things:
(1) the effects of the drugs on the mother;
(2) the effects of the drugs on the baby;
(3) if the drugs will stop an infected mother from passing the virus to her baby.

Before you decide if you wish to be in this study, you need to know about any good or bad things that could happen if you decide to join. This form tells you about the study. You can ask any questions you have at any time.

BEING IN THE STUDY IS YOUR CHOICE
This assent form tells you about the study. This information will also be discussed with you. Once you understand and decide that you and your baby should take part in this study, you will be asked to sign this assent form. We will also give you a copy to keep.

You are here because you have decided that you will breastfeed your baby. You have been told about good and bad things about breastfeeding. You have also been told that there are other feeding methods and that there are good and bad things about each of them.

Before we tell you more about the study, you should know that:
- It is your choice to join and allow your baby to join this study.
- You may decide not to join the study.
- If you choose to be in the study, you and your baby can withdraw at any time.
- If you choose to be in the study, you don't have to answer any questions that you don't want to.

The goals of this study are:
- To see if three drugs called zidovudine (ZDV), lamivudine (3TC) used with either nevirapine (NVP) or nelfinavir (NLF) can lower the risk of your baby getting HIV. You will take the drugs from the last month you are pregnant to 6 months while you breastfeed.
- To look at the safety of these drugs for pregnant women and women who breastfeed. The study will see if the women are able to take the drugs. It will see what side effects they have.

- To look at the safety of these drugs for the baby while it is breastfed. The study will see what side effects these drugs have on the baby.
- To see how many babies in the study at 24 months don't have HIV.

If you choose to be in the study, you and your baby will be in the study for 24 months after your baby is born.

WHAT HAPPENS DURING THE STUDY

- As soon as you join the study, you will have a physical exam. A member of the study staff will ask you questions about your past health. About 3 teaspoons of blood will be taken from a vein in your arm to do tests. These tests will be done to confirm your HIV status and to tell us if it is safe for you to be in the study. If it is safe for you and your baby to be in the study and you agree to join, you will be in this study from the last month of your pregnancy until 24 months after your baby is born. Your baby will be in this study for the first 24 months of his/her life. So that we can follow your and your baby's health during this time, you must live in or around Kisumu for the whole time you are in the study.

- During the time in the study we will be doing tests on your blood to make sure it is safe for you to continue to get the study drugs. We will also be monitoring how the drugs are affecting the HIV virus and the effect of the virus on your body. At each visit we will be asking you questions and filling forms. All this information is to help us understand more about the women in the study. All the information will be kept private.

- We would like to send a member of the study team to your home to check on you and your baby from time to time. In order to do this, a study staff member will go home with you and draw a map to your home in Kisumu. They will also ask you other places where you might be staying if you are not at home.

- You will be given ZDV, 3TC and either NVP or NLF to take every day. We will decide if you should get NVP or NLF based on your CD4 count in your blood and tests to see if you have any liver infection. The CD4 count is a test we can do on the blood, which gives us some information about how well a person's body is able to fight infections. HIV infection makes people's CD4 counts go down. If your CD4 count when you join the study is less than 250 cells you will be given NVP unless you have a liver infection in which case you will be given NLF. If your CD4 count when you join the study is equal to or more than 250 we will give you the drug NLF instead of NVP. You will be taking these pills twice a day from the last month that you are pregnant until 6 months after your baby is born. These drugs will lower the amount of HIV in you. They should also lower the chance of your baby getting HIV. NVP will also be given to your baby by mouth one time usually before it is 3 days old (but no later then 7 days after birth). NVP has already been shown to lower the risk of babies getting HIV during pregnancy, delivery, or breastfeeding. It is offered to all mothers with HIV at the New Nyanza Provincial Hospital. After you deliver, you will still take all three drugs the same way you did before you delivered. You will take these drugs until you stop breastfeeding or until the baby is 6 months old. If you have been taking NVP and the drugs are to be stopped, then the NVP will be stopped at 6 months and the other drugs will be continued for a further 21 days.

- While you are taking the study drugs and breastfeeding, you will need to feed your baby only breast milk. Do not add cereal or water to your baby's diet for the first 6 months. We will ask you to be part of a group that will give you ideas about how to do this.

- These medicines are also commonly used to treat people who have become sick with AIDS. They are not a cure, but do help to decrease the amount of HIV in the body. This helps people feel better and they are able to fight other infections. The decision to put people on these drugs is based on how sick they are and a blood test called a CD4 count. If you get sick with AIDS, you will not be taken off the drugs. You may be asked to take them longer. If you still need to take the drugs after the study ends, you will either continue to get medicines through our clinic or through other programs in Kisumu, which are now being set up.

- You may choose to tell or not to tell the baby's father about being in the study. If you do tell the baby's father and he does not want you and your baby to be in this study, we will help you tell him again about the study and your role in it. If he still does not want you to be in the study, you may choose to leave the study. You should make your choice based on what is best for you and your baby.

Mothers

If you agree to join and allow your baby to join this study:

- While you are pregnant, you will need to come to the clinic every week. Every 2 weeks about 3 teaspoons of blood will be taken.

- **When you come to the hospital to deliver, you will be examined by a study doctor. About 3 teaspoons of blood will be taken from you while you are in the hospital.**

- If you deliver at home, you will need to come to the clinic within 7 days of delivery. We will examine you at that visit. We also will take 3 teaspoons of blood like we would have done if you delivered at the hospital.

- At 2 weeks, 6 weeks, 14 weeks, 6 months, 9 months, 12 months, 18 months and 24 months after you give birth, we will draw your blood and give you a physical exam. Some of this blood will be stored. The blood will be stored at the clinic or CDC/KEMRI without your name on it. Instead, it will have a study code number on it. Study staff will know which code number belongs to you and your baby. No one else will be able to know that it is your blood.

- You should tell a study nurse or doctor before taking any drugs or herbals that are not part of the study.

- You will be asked to join a breast milk substudy. 150 women will be recruited into that study. If you agree, some blood and breast milk will also be taken at birth, and then 2 weeks, 6 weeks, and 6 months after the birth and kept for future tests. We will collect about 4 teaspoons of breast milk. We will also advise you to stop breastfeeding at 6 months. (See separate assent form). You can agree for you and your baby to only take part in the main study or in both the main study and the breast milk study.

Infants

- After birth, ideally within 2 to 3 days, but (within the first 7 days), your baby will be given one dose of liquid NVP.

- At birth, 2 weeks, 6 weeks, 14 weeks, 6 months, 9 months, 12 months, 18 months and 24 months of age, your baby will be examined. We will also draw about a teaspoon of blood from a vein your baby's arm. Some of this blood will be stored at the clinic or CDC/KEMRI. The blood will be stored without your or your baby's name on it. Instead, it will have a study code number on it. . Study staff will know which code number belongs to your baby. No one else will be able to know that it is your baby's blood.

- If we cannot draw your baby's blood from a vein at these visits, we will prick his/her finger or heel to get a small amount of blood.

- We will also test your baby's blood for HIV. We will test your baby for HIV 3 and 6 months after delivery and then at 24 months when you end your time in the study. When we have the test results, we will let you know your baby's HIV test results.

- If your baby gets sick with AIDS while in the study, we will provide medicines used to treat people who have become sick with AIDS. They are not a cure, but do help to decrease the amount of HIV in the body. This helps people feel better and they are able to fight other infections. If your baby gets sick with AIDS while in the study and still needs to take the drugs after the study ends, he/she will either continue to get medicines through other programs in Kisumu, which are now being set up, or our clinic.

IF YOU CHOOSE TO LEAVE THE STUDY

- You can choose to leave the study at any time. This will not affect the health care you received before you joined the study.

- If you choose to leave this study for any reason before you have your baby, you will be asked to take one dose of NVP when your labor starts. You will also be asked to allow your baby to receive one dose of NVP after birth (between 3 and 7 days). This is what the Kenyan government promotes to protect infants from getting HIV from their mothers.

- If you choose to leave the study and not take even one dose of NVP during labor, you will be asked to allow your child to receive on dose of NVP as soon as possible after birth. This one time dose is to protect your baby from becoming infected with HIV. Your baby would have a lower chance of getting HIV if you take a dose as well.

- You should tell a study nurse or doctor before joining other studies.

RISKS TO YOU

We will decide if you should get NVP or NLF based on your CD4 count and tests to see if you have any liver infection. If your CD4 count when you join the study is less than 250 cells you will be given NVP unless you have a liver infection in which case you will be given NLF. The most common side effects after many doses of NVP are skin rash, fever, nausea, headache, and blood tests showing a liver that is not normal. 1 out of 6 adult patients have rashes from NVP. Some patients have also had problems in the mouth and eyes. Severe rash happens in about 1 out of 16 patients taking NVP. This type of rash can make patients very ill. If this happens, we will tell you to stop taking NVP.

The most serious skin rash from NVP is called Stevens Johnson Syndrome. It is rare, but people with Stevens-Johnson Syndrome have bumps and blisters on their mouth and eyes. The skin and wet areas of their mouth and eyes may peel off. They may also have a fever, chills, and headache. In severe cases, the kidneys, lungs, and stomach may also have bumps and blisters. Rarely, death has been reported.

We do not know what causes this severe rash but it occurs more often when a woman's CD4 count is higher than 250. We do know that most of the severe rashes happened within the first 42 days (6 weeks) of taking NVP. To lower the chance that you will have a severe rash, we will start you with a lower dose of NVP for the first two weeks.

Severe liver damage has been found in people on chronic (long time) treatment with NVP it most commonly occurs within the first 18 weeks after a person begins taking the drug. If you have other liver infections such as hepatitis or drink alcohol while taking the study drugs, the risk to your liver is higher. You should not drink alcohol while taking NVP. Very few patients taking NVP have symptoms of liver damage. It has been reported in pregnant women as well. In very rare cases, death due to liver problems has been reported in pregnant women taking NVP. So it is important that we take your blood to check your liver while you are taking NVP.

If your CD4 count when you join the study is equal to or more than 250 we will give you the drug NLF instead of NVP. NLF has been used among pregnant women in the U.S. very often and usually has few side effects. Some women get loose stools, gas or other mild symptoms. Also some have had changes in blood sugar and levels of fats in their blood; and some changes to body fat. Most women tolerate NLF well without problems.

The drugs 3TC and ZDV are safe and very effective when taken at the same time. There is a small chance that 3TC or ZDV may cause damage in the liver or blood such as low blood (anemia) or lower your cells that fight infections. They may also cause headache, sleeplessness, and muscle aches. Many studies in Africa, Europe, and the U.S.A. have shown that 3TC and ZDV are generally safe in pregnant women. We will do blood tests to check for these side effects on you and your baby during the study.

If you decide to enter the study and are found to be eligible, we will tell you what to do in the event that you have one of these side effects**.**

RISKS TO THE FETUS/YOUR BABY

The most common side effects from NVP in infants and children are similar to those in adults. But some children also develop blood changes that are not common in adults taking NVP. Serious side effects are rare. They include Stevens Johnson Syndrome (described above) and severe allergic reactions. The most common side effect in babies is a mild rash. You should tell your doctor and a study staff member about any rash on your baby as soon as possible.

Studies have shown that early breast milk can have NVP in it. As stated above, a rash can occur when taking NVP. Babies taking milk from mothers on NVP may develop a rash. Again, you should tell your doctor or other study staff about any rash on your baby as soon as possible.

Other studies have shown that there are small amounts of 3TC and ZDV in breast milk. These doses are small, so the chances of the drugs causing low blood (anemia) or other health problems in your baby are very low. We will check your baby's blood to make sure that these drugs are not causing any health problems.

All the drugs; ZDV, 3TC, NVP and NLF have been used a lot during pregnancy. No major problems have been noticed in the babies whose mothers took these drugs while pregnant..

Blood drawing may cause some pain, bleeding, or bruising where the needle goes through the skin. It may also cause some lightheadedness or dizziness. A small blood clot may form where the needle enters the body. Swelling of the skin in the area may occur. There is also a small risk of a minor infection on the skin where the blood is drawn.

BENEFITS TO YOU AND/OR YOUR BABY

Taking NVP for a short amount of time can lower the risk of passing HIV from a pregnant mother to her baby. Taking ZDV and 3TC together for a short amount of time can also lower the risk of passing HIV from an infected mother to her infant. Because taking ZDV, 3TC and NVP or NLF for a short amount of time can decrease the risk of passing HIV to your baby, using these drugs for longer amounts of time should work in protecting your baby from HIV.

In studies from the US, few mothers with HIV passed the virus to their babies when they were taking all 3 drugs. But these studies were from mothers who did not breastfeed. Your baby should do very well if he or she is able to take the drug and not have bad side effects.

You and your baby still may or may not benefit directly from this study. But what we learn may be useful in finding a treatment to prevent other HIV-infected mothers from passing the virus to their babies.

NEW FINDINGS

You will be told of any new findings learned during the study. These findings may cause you to change your mind about you and your baby staying in the study. At the end of the study, you will be told when study results may be known and how to learn about them.

REASONS FOR TAKING YOU OUT OF THE STUDY WITHOUT YOUR ASSENT

You and your baby may be taken out of the study without your assent if:

1) Your doctor or your baby's doctor decides that staying in the study may cause harm to you and/or your baby;

- Your baby is born with health problems that might cause him/her to die;
- You or your baby need(s) a treatment not allowed on this study;
- You are unable to keep clinic visits;
- You are unable to take the study drugs as told;
- The study is stopped by the CDC, KEMRI, or MOH.
- Other reasons beyond our control.

You will not be given or not allowed to keep taking ZDV, 3TC, NVP or NLF if:

a. Before delivery, you have high blood pressure, severe headache, too much protein in your urine, and severe swelling of the legs and feet.
b. During labor, you have seizures.
c. During labor, you have problems that must be treated with drugs that stop your blood from clotting.
d. You have a severe infection that may cause shock.
e. After delivery, you have high blood pressure that does not get better with treatment.
f. You cannot take pills by mouth.

g. Your blood test shows that your liver is not normal or that you have severe low blood (anemia).

Your baby will not be given NVP if he/she has serious health problems and:
- Cannot take the study drug by mouth.
- His/her blood test shows that the liver is not normal.

COSTS TO YOU
- There is no cost to you or your baby for the study drugs, study clinic visits, physical exams, transport to study clinic visits, lab tests for the study, or for your delivery at the hospital.

- Medical care for common medical problems will be given to you and your baby at the study clinic at no cost to you. If you have to be staying overnight at the New Nyanza Provincial General Hospital for health reasons, CDC/KEMRI will pay for the services.

- The health care described above will be given as long as you are in the study.

- Neither you nor your baby will be paid for being in this study.

YOUR RECORDS WILL BE PRIVATE
You and/or your baby's research records will be kept private as allowed by the law. You and your baby will be given code numbers, and any private information from your and your baby's records will not be given out unless you agree in writing. You and your baby’s names will not be in any reports about this study. But, people who review the study for KEMRI and CDC may need to look at your and your baby’s records from time to time. In order to give you or your baby good medical care and for study needs, we will need to make copies of your medical records from Provincial Hospital and other places where you may get medical care. All copies will be kept in a locked file cabinet. Only study staff will have access to this cabinet.

INJURY BECAUSE OF BEING IN THE STUDY
If you and/or your baby have side effects from the drugs or something bad happens because of being in this study, CDC/KEMRI will find treatment for you and/or your baby as soon as possible. CDC/KEMRI will pay for this treatment.

When you sign this assent form, you do not give up your rights to care for injury caused by being in the study.

PICTURES

We will ask to take your picture. This will help us make sure that no one else can get the medication that is meant for you. It will also help us find you in case of emergency. This picture will not have your name on it, only your study identification number. It will be kept in a secure place with your records. At the end of the study the picture will be given to you if you want it, or it will be destroyed. If you do not want your picture taken, we will not take it. If you chose not to have your picture taken, it will not affect your ability to take part in the study in any way.

- ☐ I assent to have my picture taken.
- ☐ I DO NOT assent to have my picture taken.

PROBLEMS OR QUESTIONS

If you have questions or concerns about this study, contact:

- Dr. Timothy Thomas at the CDC/KEMRI study clinic at New Nyanza Provincial General Hospital on Kakamega Road, Kisumu (P.O. Box 1578, Kisumu) or phone number: 057-2023200; or
- Dr. Wairimu Chege of CDC/KEMRI Kisian Office off Kisumu-Busia Road (P.O. Box 1578, Kisumu) or phone: 0572022983/2022959/2022902.

If you have any questions, including about a research subject's rights, you should talk to Margaret Odhiambo at the Provincial General Hospital or phone: 057 44278.

STATEMENT OF ASSENT

Please sign your name below once you:

have read (have been explained) and know the reasons for the study,
know the steps to be followed in the study,
know the risks and benefits to you and your baby from being in the study,
AND choose to enroll you and your baby in this study of your own free will.

**Volunteer's Name**<br>*Type or print*	**Volunteer's Signature**<br>(Volunteer signs here if she is under age 18)	**Date**

**Witness's Name**<br>*Type or print*	**Witness's Signature**	**Date**

I have explained the purpose of this study to the volunteer. To the best of my knowledge, she understands the purpose, procedures, risks and benefits of this study.

**Investigator/Designee Name**<br>*Type or print*	**Investigator/Designee Signature**	**Date**

NOTE: This assent form with original signatures must be retained on file by the principal investigator. A copy must be given to the volunteer. A copy should be placed in the volunteer's medical record (if applicable).

BREAST MILK SUBSTUDY

Quantifying the Exposure to Antiretroviral Drugs through Breast milk among Women taking Highly-Active Antiretroviral Therapy

Concept sheet developed by:
Paul J. Weidle, Pharm.D., MPH – Senior Staff Epidemiologist, Epi Branch, DHAP, NCHSTP
Mary Glenn Fowler, MD – Chief MCT-PASS Section, Epi Branch, DHAP, NCHSTP
Mark Mirochnick, MD – Boston University

Background

Programs to provide antiretroviral drugs to persons in resource-poor settings are being developed and generally will include patients with advanced HIV disease, as evidenced by symptomatic disease or confirmed by a CD4+ cell count < 200 cells/mm^3. Some participants in these programs will be women of child bearing potential, pregnant women, and women who are breastfeeding. In resource-poor settings, the World Health Organization/UNAIDS recommends that when replacement feeding is acceptable, feasible, affordable, sustainable, and safe, that HIV infected mothers avoid all breastfeeding. However, when these conditions do not exist, exclusive breastfeeding is recommended for the first six months of life.

It is recognized that HIV can be transmitted through breast milk and hypothesized that providing antiretroviral drugs to a breastfeeding woman will decrease the risk of transmission of HIV through a decrease in viral load. A Phase II open label trial of maternal zidovudine/lamivudine and nevirapine for maximal reduction of mother-to-child HIV transmission in resource limited settings among breast feeding populations is being developed in Kisumu, Kenya to evaluate the impact of triple drug HAART regimens at decreasing transmission via breastfeeding. We propose to use some of the stored breast milk and plasma samples to quantify the exposure to antiretroviral drugs through breast milk.

Drugs in Breast Milk

The transfer of medication into breast milk is driven primarily by a concentration gradient that allows passive diffusion of non-protein bound drugs [Spencer 2001]. The concentration of drugs in breast milk is determined primarily by maternal serum drug concentrations. The serum concentration of drugs with short half-lives fluctuates greatly over the course of a dosing interval, and as such, it would be expected that the concentration in breast milk fluctuates in a manner that is roughly parallel. However, the serum concentration of drugs with long half-lives fluctuates less over a dosing interval and would be expected to produce more sustained levels of drug in breast milk.

There is evidence, from animals and humans, that antiretroviral drugs penetrate into breast milk. Table 1 summarizes information on transfer of antiretroviral drugs into breast milk [Safety and Toxicity of Antiretroviral Drugs 2002].

Table 1. Transfer of antiretroviral drugs into breast milk

	Evidence of transfer into breast milk		
Drug	Animal Data	Human Data	Comment

Abacavir	Yes		
Didanosine	Yes		
Lamivudine		Yes	
Stavudine	Yes		
Tenofovir			No data
Zalcitabine			No data
Zidovudine		Yes	
Delavirdine	Yes		
Efavirenz			No data
Nevirapine		Yes	Milk-to-plasma ratio<br>0.76 (0.54 – 1.04) - Mirochnick<br>0.61 (0.25 – 1.22) - Musoke
Amprenavir	Yes		
Indinavir	Yes		Milk-to-plasma ratio = 1.26 – 1.45
Lopinavir	Yes		
Nelfinavir	Yes		
Ritonavir	Yes		
Saquinavir	Yes		

Nevirapine

Nevirapine, a drug commonly use to prevent transmission of HIV during labor and deliver, is a drug with a half-life of ~45 hours after a single dose and 25-30 hours after repeated doses [Nevirapine Package Insert]. As reported in nevirapine product labeling, the peak serum concentration after a single 200 mg dose is 2 +/- 0.4 ug/ml. When dosed at the usual treatment dose of 200 mg twice daily, the steady-state trough concentration is 4.5 +/- 1.9 ug/ml.

Mirochnick studied the pharmacokinetics of a single 200 mg dose of nevirapine to pregnant women and their neonates [Mirochnick 1998]. Paired results of 4 breast milk and serum samples from 3 women were used to determine that the median milk-to-plasma ratio was 0.76 (range, 0.54 – 1.04).

Musoke, et al also studied the pharmacokinetics of a single 200 mg dose of nevirapine in Uganda and found a milk-to-plasma ratio of 0.61 (range, 0.25 – 1.22) [Musoke 1999]. They also found that breast milk nevirapine concentrations declined from a median of 454 ng/ml (range, 219-972) at 48 hours after birth to 103 ng/ml (range 25-309) at day 7 in a manner parallel to the decline in maternal plasma nevirapine concentrations. They estimated that the median ingestion of nevirapine from breast milk by the infant was 0.06 mg/kg on day 2 and 0.02 mg/kg on day 7.

The recommended dose for prevention of mother to child transmission of HIV is to give the mother a single 200 mg at onset of labor and to give the infant 2 mg/kg as a single dose within 72 hours of delivery. However, for chronic dosing, the recommended adult dose is 200 mg once daily for 7 days, then 200 mg twice daily thereafter. The recommended treatment dose for a child $\geq$ 2 months old is 4 mg/kg for the first 14 days, then 7 mg/kg twice daily thereafter.

Using the estimated steady-state trough concentration and milk-to-plasma ratio listed above, and considering that an infant will ingest 150 – 180 ml/Kg of breast milk per day, it is estimated that a 3 kg infant will ingest ~1.5 – 1.8 mg nevirapine/day during the first few weeks of life. By six months of age a 7 kg child will ingest ~3.5 – 4.2 mg nevirapine/day. Thus, shortly after birth the

infant would be ingesting ~13-15% of the recommended dose and by six months ~4% of a treatment dose.

Zidovudine and Lamivudine
Zidovudine and lamivudine are drugs with relatively short serum half-lives (zidovudine ~ 1 – 3 hours, lamivudine ~5-7 hours). Both have been demonstrated to be present in breast milk in humans. The product labeling for zidovudine states that the mean concentration in breast milk was similar to that in serum [Retrovir Package Insert].

Moodley reported mean concentrations of lamivudine in breast milk of 1.22 ug/ml (range, <0.5 – 6.09) and 0.9 ug/ml (<0.5-8.2) when used alone or in combination with zidovudine, respectively. This is roughly similar to the reported peak concentration of lamivudine [Epivir Package Insert]. Moodley, et al report that the amount of lamivudine a neonate would ingest is negligible relative to standard oral dosing. For instance, at a concentration of ~1 ug/ml, an infant would ingest ~ 0.09 mg during a 3 ounce feeding (range, <0.04 – 0.7 mg per feeding). Over a 24-hour period this would amount to far less than the standard dose of 4mg/kg twice daily. The wide range in breast milk concentrations is reflective of the fluctuation in serum concentrations over a dosing interval.

Nelfinavir

Nelfinavir is a protease inhibitor commonly used for pregnant women. It has a half-lifein serum of 3.5-5hours and achieves adequate concentration in pregnant women. Nelfinavir is present in breast milk of lactating rats, but it is unknown if it is excreted into human breastmilk

Implications
The implication of this exposure to antiretroviral drugs via breast milk is that a child may develop an idiosyncratic toxic reaction from low dose exposure to one or more of the drugs. This is most concerning for nevirapine, for which development of a rash or hepatotoxicity are most likely to be problematic. The current study in Kisumu is monitoring for such reactions in the children.

There is also an implication related to potential unintended antiviral activity of antiretroviral drugs. The expectation is that the amount of nucleoside RTIs the child would ingest is much lower than the amount of nevirapine. If the child is not HIV infected, and remains so during the period of breastfeeding, there is no consequence of this. However, if the child becomes HIV infected, either prior to the initiation of or during the mother's antiretroviral therapy, and does not receive antiretroviral therapy independently, there is a risk of development of resistance from chronic administration of low doses of antiretroviral drugs, especially nevirapine or lamivudine, ingested through breast milk. This could theoretically affect future treatment options for the child.

Quantification of exposure to antiretroviral drugs through breast milk has relevance to the study in question, considering that some of the children are expected to become infected with HIV either prior to delivery or during the breastfeeding period. However, it has greater implications for generalizing to other populations of HIV-infected mothers in future treatment programs who

are breastfeeding children and are then begun on antiretroviral drugs. An understanding of the risk to the child of "unintended" administration of low levels of antiretroviral drugs will help to inform programs on the need for replacement feedings, if practical, and treatment programs for the infants as well.

Proposal

We propose to use portions of the paired stored maternal plasma and breast milk and infant plasma at delivery, 2 weeks, 6 weeks, 14 weeks, and 6 months for drug concentration determination among 150 mothers and their infants from the KiBS who enroll in the breast milk substudy in Kisumu, Kenya. In addition, samples of plasma, blood, and breast milk will be collected at 9 months, 12 months, 18 months, and 24 months from women who continue to breastfeed. At each time point, a maximum of 20ml breastmilk will be obtained, if available. (The quantity available at delivery may be limited). Three to 5 ml of whole milk will be aliquoted into 1.5 – 2 ml tubes that will then be frozen and stored at -70^{0}C. Estimates of exposure will be modeled using NON-mixed effects modeling (Non-MEM) pharmacokinetic techniques.

The relevant information we need to study this issue includes height and weight of the mother and the length and weight of child at each time point of sampling. We would also need accurate timing of the last dose of each antiretroviral drug by the mother, and the timing of collection of maternal plasma and breast milk and the child's specimen. We may also need timing of the last breastfeeding. The timing of these events is crucial to constructing an accurate model of maternal plasma to breast milk transfer and antiretroviral exposure through breast milk in the child. Mechanisms to accurately collect these data include: 1) reliable, accurate self-report of the exact time of drug ingestion by the woman; 2) direct observation in the clinic of the dose of each antiretroviral drug prior to specimen collection; or 3) electronic recording of the time of ingestion of the antiretroviral drugs by use of a device such as a Medication Events Monitoring System (MEMS). There are challenges that will need to be addressed with any of these efforts. For example, need for a clock or watch in the household for #1, the need to coordinate the timing of drug ingestion and the clinic visit for #2, and management and training of MEMS caps use for #3. Support will be obtained to carry out the identified mechanism(s).

At this time, we do not propose to do further sampling of the women or children. However, if interest and resources permit, a more focused study of 10 – 20 women contributing multiple specimens of plasma and breast milk over an 8 hour period could be designed to more accurately describe the pharmacokinetics of these antiretroviral drugs in maternal plasma and breast milk.

We also propose to test for resistance of HIV to antiretroviral drugs in HIV-infected infants paired to plasma and breast milk samples from their mothers. We will test for genotypic mutations associated with resistance to HIV and possibly for phenotypic resistance as well. The genetic markers for resistance for the drugs used in this study (zidovudine, lamivudine, nevirapine and nelfinavir) are reasonably well understood. The specifics for the testing are not determined at this point, but the genotypic test will likely be based on standard sequencing methods of the *pol* gene of HIV. From the sequence we could also determine the likely subtype of HIV as well.

Objectives of the proposed study are:

- Describe steady state concentrations of zidovudine, lamivudine, and nevirapine in maternal plasma and breast milk.
- Assess if zidovudine, lamivudine, and nevirapine can be detected in samples from breastfeeding infants and model the total dose per day ingested via breast milk at shortly after birth, at 6 weeks, and at 6 months.
- Assess for possible detectable HIV-resistant mutants through genotypic or phenotypic HIV-resistance testing from HIV-infected infants paired to their mothers' plasma and breast milk.

Funding:
Funding has been secured from NIH.

References

Safety and toxicity of individual antiretroviral agents in pregnancy. Available at http://www.hivatis.org, accessed February 4, 2002.

Spencer JP, Gonzalez LS, Barnhart DJ. Medications in the breastfeeding mother. *American Family Physician* 2001;64:119-26.

Mirochnick M, Fenton, T, Gagnier P, et al. Pharmacokinetics of nevirapine in human immunodeficiency virus type 1-infected pregnant women and their neonates. *J Infect Dis* 1998;178:368-74.

Musoke P, Guay LA, Bagenda D, et al. A phase I/II study of the safety and pharmacokinetics of nevirapine in HIV-1 infected pregnant Ugandan women and their neonates (HIVNET 006). *AIDS* 1999;13:479-86.

Moodley J, Moodley D, Pillay K, et al. Pharmacokinetics and antiretroviral activity of lamivudine alone or when coadministered with zidovudine in human immunodeficiency virus type 1-infected pregnant women and their offspring. *J Infec Dis* 1998;178:1327-33.

Viramune Package Insert. Roxane Laboratories, Inc.

Retrovir Package Insert. GlaxoSmithKline.

Epivir Package Insert. GlaxoSmithKline.

BREAST MILK SUBSTUDY CONSENT FORM

(Consent 05A)

This form covers the Mother, fetus and infant. It must either be signed by the mother if she is aged 18 or older, OR by a legal guardian of the mother if she is under 18 years of age. If the mother is under 18 years of age she must sign the assent form.

Flesch-Kincaid Score: 6.9

TITLE OF SUBSTUDY: Breast milk study in Kisumu, Kenya

INFORMED CONSENT

Because you are already in the Kisumu Breastfeeding Study (KiBS), you are one of the 150 women in the study being asked to be in a smaller sub-study on breast milk. You and your baby are still a part of KiBS if you agree to be in this smaller breast milk study.

This sub-study is also from the U.S. Centers for Disease Control and Prevention (CDC), the Kenya Medical Research Institute (KEMRI), and the Kenya Ministry of Health (MOH). This study will look at how three drugs: (zidovudine (ZDV), lamivudine (3TC) and either nevirapine or nelfinavir affect blood and breast milk. We will also look at how the 3 drugs affect the parts of blood and breast milk that can reduce or increase the risk of HIV infection in babies.

If you agree to be in this study, we will test some of the blood and breast milk that you will give in KiBS study. Some of it will be stored at the study clinic or CDC/KEMRI for later testing.

Before you decide if you want to be in this study, you should know the reasons for the study. You should know how the study might help you and your baby. You should be aware of any risks to you and your baby from being in the study. You should also know what is expected of you and your baby if you are in the study. You can ask any questions you have at any time.

BEING IN THE STUDY YOUR CHOICE

This consent form tells you about the study. This information will also be discussed with you. Once you understand and want yourself and your baby to be in the study, you will be asked to sign this consent form. We will also give you a copy to keep.

Before you learn about the breast milk sub study, you should know that:

- It is your choice to join and allow your baby to join this study.
- You may decide not to join the breast milk sub study and still be part of the KiBS study.
- If you choose to be in the study, you may withdraw yourself and your baby at any time.
    - If you choose to be in the study, you don't have to answer any questions that you don't want to.

The goals of this study are:

- To see how the three drugs that you take called, zidovudine (ZDV), and lamivudine (3TC) and either nevirapine or nelfinavir affect the amount of HIV virus in your breast milk.
- To look at any changes in the virus that may make the drugs less useful against HIV.

- To measure the amount of each drug in the breast milk.
- To describe other ways that the drugs may affect the breast milk.

WHAT HAPPENS DURING THE STUDY

  - If you decide to be in this sub-study, you must sign this consent form.
  - We will test and store part of the blood that you give in KiBS at delivery, 2 weeks, 6 weeks, 14 weeks, and 6 months after delivery.
  - We will also collect about 4 teaspoons of breast milk at delivery, 2 weeks, 6 weeks, 14 weeks, and 6 months after delivery.
  - When you breast feed you will be asked to give your baby only breast milk for the first 6 months of life for the baby's overall health. Do not give the baby water, cereal, porridge, cow's milk, or anything else besides breast milk.
  - The study clinical officer or doctor will ask you questions about how, when, and what you feed your baby.
  - After 6 months, you will be advised to stop breastfeeding your baby.

- The blood and breast milk will be stored at the study clinic or CDC/KEMRI without your name on it. Instead, it will have the study code on it. The record of your name and study code number will be safely locked in a file in the study clinic. Study staff will know which code number belongs to your baby. No one else will be able to know that it is your blood or breast milk.

- The blood and breast milk will later be tested for things like: amount of HIV virus, amount of drug, changes in the virus that may make the drugs less useful in fighting the virus; and parts of the blood and breast milk that may help protect against HIV infection in babies.

- If you choose to be in this breast milk sub-study, you will be in the study until you stop breast feeding or 24 months after you give birth, whichever comes first.

- If you choose to be in this breast milk sub-study, you will be asked to use a special medicine bottle. This bottle can record every time it is opened. You will be asked to bring this bottle with you every time you come to this clinic. At the end of the study, you will be given 200 KSH if you return your bottle and its cap.

- You should tell a study nurse or doctor before taking any drugs or herbals that are not part of the study.

- You should tell a study nurse or doctor before enrolling in other studies.

- You may or may not choose to tell the baby's father about being in the sub-study. If you do tell the baby's father and he does not want you and your baby to be in this sub-study, we will help you tell him again about the sub-study and your role in it. If he still does not want you to be in the sub-study, you may choose to leave the study. If you decide not to be in the sub-study, you can still be in the larger study. You should make your choice based on what is best for you and your child.

Infants
We will look at the results of your blood tests and your baby's blood tests from the larger KiBS study as well as drug levels. No extra blood will be drawn from your baby.

If you choose to leave the study
You can choose to leave the sub-study or larger HAART study at any time. This will not have any affect on the regular health care you receive.

RISKS TO YOU AND YOUR BABY
There is very little risk to you for being in the breast milk sub-study. There may be some discomfort in expressing the breast milk into the collection tube.

There is very little risk to your baby. Your breasts should make enough milk that your baby will not miss the small amount of milk we collect.

BENEFITS TO YOU AND/OR YOUR BABY
You and your baby may or may not benefit directly from this study. But other mothers and babies should benefit from what we learn in this study. With your help, we will learn much about the affect of these drugs on breast milk. We will learn how the HIV virus acts in breast milk. We will learn what can make breast milk more likely or less likely to spread HIV to babies. What we learn may be useful in finding ways to prevent other HIV-infected mothers from passing the virus to their babies.

NEW FINDINGS
You will be told of any new findings learned during the study. At the end of the study, you will be told when study results may be known and how to learn about them.

REASONS FOR TAKING YOU OUT OF THE STUDY WITHOUT YOUR CONSENT
You may be taken out of the study without your consent if:

- You are taken out of the larger KiBS study for the reasons explained in the consent form for KiBS;
  - You are unable to give the breast milk;
  - The study staff believe that being in the breast milk sub-study is harmful to you or your baby;
- Other reasons beyond our control.

COSTS TO YOU

There is no cost to you for being in the sub-study.

- Containers for taking the breast milk and all lab tests will be paid for by CDC/KEMRI.
- You will not be paid for being in this sub-study.
- You will be given money to pay for transportation to and from the study clinic.

YOUR RECORDS WILL BE PRIVATE

Your research records will be kept private as allowed by the law. You will be given a unique code, and any private information from your records will not be given out unless you agree in writing. Your name will not be in any reports written about this study. But , people who review the study for KEMRI and CDC may need to look at your and your baby's record from time to time.

INJURY BECAUSE OF BEING IN THE SUB-STUDY

CDC/KEMRI will find treatment for you if you need medical care because of being in the sub-study and giving breast milk. CDC/KEMRI will pay for this treatment. The same policy will be in place as is for the larger study.

When you sign this consent form, you do not give up your rights to care for injury caused by being in the study.

PROBLEMS OR QUESTIONS

If you have questions or concerns about this study, contact:

- Dr. Timothy Thomas at the CDC/KEMRI study clinic at New Nyanza Provincial Hospital on Kakamega Road, Kisumu (P.O. Box 1578, Kisumu) or phone number: 035-23200; or
- Dr. Wairimu Chege of CDC/KEMRI Kisian Office off Kisumu-Busia Road (P.O. Box 1578, Kisumu) or phone: 0572022983/2022959/2022902.

If you have questions, including those about a research subject's rights, you should talk to Margaret Odhiambo at the Provincial General Hospital or phone: 035 44278.

STATEMENT OF CONSENT

Please sign your name below once you:

- have read (have been explained) and know the reasons for the sub-study,
- know the steps to be followed in the sub-study,
- know the risks and benefits to you and your baby from being in the sub-study,
- AND choose to enroll in this study of your own free will.

____________________	____________________	____________________
Volunteer's Name<br>*Type or print*	Volunteer's Signature<br>(Volunteer does NOT sign here if she is under age 18)	Date

____________________	____________________	____________________
Volunteer's Legal Guardian or Representative (if <18 years old)<br>*Type or print*	Legal Guardian's Signature	Date

____________________	____________________	____________________
Witness's Name<br>*Type or print*	Witness's Signature	Date

I have explained the purpose of this sub-study to the volunteer and/or her legal guardian. To the best of my knowledge, she or he understands the purpose, procedures, risks and benefits of this study.

____________________	____________________	____________________
Investigator's Name<br>*Type or print*	Investigator's Signature	Date

NOTE: This consent form with original signatures must be retained on file by the principal investigator. A copy must be given to the volunteer. A copy should be placed in the volunteer's medical record (if applicable).

BREAST MILK SUBSTUDY ASSENT FORM
(Assent 06A)

This form covers the Mother, fetus and infant. It must be signed by the mother if she is under 18 years of age. A legal guardian of the mother must sign the consent form.

Flesch-Kincaid Score: 6.9

TITLE OF SUBSTUDY: Breast milk study in Kisumu, Kenya

INFORMED CONSENT

Because you are already in the Kisumu Breastfeeding Study (KiBS), you are one of the 150 women in the study being asked to be in a smaller sub-study on breast milk. You and your baby are still a part of KiBS if you agree to be in this smaller breast milk study.

This sub-study is also from the U.S. Centers for Disease Control and Prevention (CDC), the Kenya Medical Research Institute (KEMRI), and the Kenya Ministry of Health (MOH). This study will look at how three drugs: (zidovudine (ZDV), lamivudine (3TC) and either nevirapine or nelfinavir affect blood and breast milk. We will also look at how the 3 drugs affect the parts of blood and breast milk that can reduce or increase the risk of HIV infection in babies.

If you agree to be in this study, we will test some of the blood and breast milk that you will give in KiBS study. Some of it will be stored at the study clinic or CDC/KEMRI for later testing.

Before you decide if you want to be in this study, you should know the reasons for the study. You should know how the study might help you and your baby. You should be aware of any risks to you and your baby from being in the study. You should also know what is expected of you and your baby if you are in the study. You can ask any questions you have at any time.

BEING IN THE STUDY YOUR CHOICE

This consent form tells you about the study. This information will also be discussed with you. Once you understand and want yourself and your baby to be in the study, you will be asked to sign this consent form. We will also give you a copy to keep.

Before you learn about the breast milk sub study, you should know that:

- It is your choice to join and allow your baby to join this study.
- You may decide not to join the breast milk sub study and still be part of the KiBS study.
- If you choose to be in the study, you may withdraw yourself and your baby at any time.
    - If you choose to be in the study, you don’t have to answer any questions that you don’t want to.

The goals of this study are:

- To see how the three drugs that you take called, zidovudine (ZDV), and lamivudine (3TC) and either nevirapine or nelfinavir affect the amount of HIV virus in your breast milk.
- To look at any changes in the virus that may make the drugs less useful against HIV.

- To measure the amount of each drug in the breast milk.
- To describe other ways that the drugs may affect the breast milk.

WHAT HAPPENS DURING THE STUDY

- If you decide to be in this sub-study, you must sign this consent form.
- We will test and store part of the blood that you give in KiBS at delivery, 2 weeks, 6 weeks, 14 weeks, and 6 months after delivery.
- We will also collect about 4 teaspoons of breast milk at delivery, 2 weeks, 6 weeks, 14 weeks, and 6 months after delivery.
- When you breast feed you will be asked to give your baby only breast milk for the first 6 months of life for the baby's overall health. Do not give the baby water, cereal, porridge, cow's milk, or anything else besides breast milk.
- The study clinical officer or doctor will ask you questions about how, when, and what you feed your baby.
- After 6 months, you will be advised to stop breastfeeding your baby.

- The blood and breast milk will be stored at the study clinic or CDC/KEMRI without your name on it. Instead, it will have the study code on it. The record of your name and study code number will be safely locked in a file in the study clinic. Study staff will know which code number belongs to your baby. No one else will be able to know that it is your blood or breast milk.
- The blood and breast milk will later be tested for things like: amount of HIV virus, amount of drug, changes in the virus that may make the drugs less useful in fighting the virus; and parts of the blood and breast milk that may help protect against HIV infection in babies.
- If you choose to be in this breast milk sub-study, you will be in the study until you stop breast feeding or 24 months after you give birth, whichever comes first.
- If you choose to be in this breast milk sub-study, you will be asked to use a special medicine bottle. This bottle can record every time it is opened. You will be asked to bring this bottle with you every time you come to this clinic. At the end of the study, you will be given 200 KSH if you return your bottle and its cap.
- You should tell a study nurse or doctor before taking any drugs or herbals that are not part of the study.
- You should tell a study nurse or doctor before enrolling in other studies.
- You may or may not choose to tell the baby's father about being in the sub-study. If you do tell the baby's father and he does not want you and your baby to be in this sub-study, we will

help you tell him again about the sub-study and your role in it. If he still does not want you to be in the sub-study, you may choose to leave the study. If you decide not to be in the sub-study, you can still be in the larger study. You should make your choice based on what is best for you and your child.

Infants
We will look at the results of your blood tests and your baby's blood tests from the larger KiBS study as well as drug levels. No extra blood will be drawn from your baby.

If you choose to leave the study
You can choose to leave the sub-study or larger HAART study at any time. This will not have any affect on the regular health care you receive.

RISKS TO YOU AND YOUR BABY
There is very little risk to you for being in the breast milk sub-study. There may be some discomfort in expressing the breast milk into the collection tube.

There is very little risk to your baby. Your breasts should make enough milk that your baby will not miss the small amount of milk we collect.

BENEFITS TO YOU AND/OR YOUR BABY
You and your baby may or may not benefit directly from this study. But other mothers and babies should benefit from what we learn in this study. With your help, we will learn much about the affect of these drugs on breast milk. We will learn how the HIV virus acts in breast milk. We will learn what can make breast milk more likely or less likely to spread HIV to babies. What we learn may be useful in finding ways to prevent other HIV-infected mothers from passing the virus to their babies.

NEW FINDINGS
You will be told of any new findings learned during the study. At the end of the study, you will be told when study results may be known and how to learn about them.

REASONS FOR TAKING YOU OUT OF THE STUDY WITHOUT YOUR CONSENT
You may be taken out of the study without your consent if:
- You are taken out of the larger KiBS study for the reasons explained in the consent form for KiBS;
  - You are unable to give the breast milk;
  - The study staff believe that being in the breast milk sub-study is harmful to you or your baby;
- Other reasons beyond our control.

COSTS TO YOU
There is no cost to you for being in the sub-study.
- Containers for taking the breast milk and all lab tests will be paid for by CDC/KEMRI.
- You will not be paid for being in this sub-study.

- You will be given money to pay for transportation to and from the study clinic.

YOUR RECORDS WILL BE PRIVATE
Your research records will be kept private as allowed by the law. You will be given a unique code, and any private information from your records will not be given out unless you agree in writing. Your name will not be in any reports written about this study. But , people who review the study for KEMRI and CDC may need to look at your and your baby's record from time to time.

INJURY BECAUSE OF BEING IN THE SUB-STUDY
CDC/KEMRI will find treatment for you if you need medical care because of being in the sub-study and giving breast milk. CDC/KEMRI will pay for this treatment. The same policy will be in place as is for the larger study.

When you sign this consent form, you do not give up your rights to care for injury caused by being in the study.

PROBLEMS OR QUESTIONS
If you have questions or concerns about this study, contact:

- Dr. Timothy Thomas at the CDC/KEMRI study clinic at New Nyanza Provincial Hospital on Kakamega Road, Kisumu (P.O. Box 1578, Kisumu) or phone number: 035-23200; or
- Dr. Wairimu Chege of CDC/KEMRI Kisian Office off Kisumu-Busia Road (P.O. Box 1578, Kisumu) or phone: 0572022983/2022959/2022902.

If you have questions, including those about a research subject's rights, you should talk to Margaret Odhiambo at the Provincial General Hospital or phone: 035 44278.

STATEMENT OF CONSENT

Please sign your name below once you:

- have read (have been explained) and know the reasons for the sub-study,
- know the steps to be followed in the sub-study,
- know the risks and benefits to you and your baby from being in the sub-study,
- AND choose to enroll in this study of your own free will.

____________________	____________________	________________
Volunteer's Name<br>*Type or print*	Volunteer's Signature<br>(Volunteer signs here if she is under age 18)	Date

____________________	____________________	________________
Witness's Name<br>*Type or print*	Witness's Signature	Date

I have explained the purpose of storing specimens to the volunteer. To the best of my knowledge, s/he understands the purpose of the storage of specimens.

____________________	____________________	________________
Investigator's Name<br>*Type or print*	Investigator's Signature	Date

NOTE: This assent form with original signatures must be retained on file by the principal investigator. A copy must be given to the volunteer. A copy should be placed in the volunteer's medical record (if applicable).

GUIDE TO HYGIENE DISCUSSION WITH KiBS MOTHERS

This document provides a guide for the topics that must be covered by the nutritionist or other member of the study team and either in the clinic or during home visits. These topics will be covered during visits both before and after delivery with particular emphasis during the peri-weaning visits. Topics may be repeated.

The discussions will cover the following topics:

Clean safe preparation of feeds
The nutritionist discusses the following in details (when, why and how)

- Proper and frequent hand washing with soap
- Clean utensils
- Safe water and food
- Safe storage of food

Feeding techniques

- Cup feeding verses bottle feeding – discourage bottle feeding
- Making transition from exclusive breastfeeding to complementary feeding – gradually reduce breastfeeding frequency while increasing frequency of other fluids.
- Breast milk substitutes; gradually introduce new foods one by one starting with fluids then mashed foods
- Use of locally available cereals for porridge is encouraged with tips given on how to enrich the porridge. Mixing more than 3 different types of cereals is discouraged

Water treatment

- Boiling water – keep water at rolling boil for 2 minutes
- Chemical disinfection – a bottle of dilute locally produced sodium hypochlorite solution (water guard) is provided to each mother and demonstration done on its use.
- Water storage container is also provided to each mother
- Store water safely to prevent recontamination

Psychosocial stimulation

- Spend time with the baby
- Cradle the baby during feeding
- Take your time when feeding the baby
- Snuggle with the baby for a few minutes after each feed
- Sleep with the baby after weaning has been completed
- Comfort and stimulate the baby with massage

Other components of hygiene

- Having and using toilets properly
- Good drainage
- Proper waste disposal
- General clean home environment
- Other kids holding the baby should wash hands

STUDY DRUG INFORMATION SHEET

WHAT YOU NEED TO KNOW ABOUT YOUR STUDY DRUGS AND TAKING THEM AS INSTRUCTED

1 The study drugs (Combivir, nevirapine, and nelfinavir) are also called Antiretrovirals (ARVs). ARVs are drugs taken for treatment of HIV/ AIDS. ARVs are NOT a cure for HIV/AIDS.

However, they can help to control HIV and make people live longer and healthier lives.

2 ARVs may decrease the amount of HIV in the body, but people on ARVs can still transmit HIV to sex partners.

Not having sex at all is the only way to guarantee that HIV is not spread through sex. There are other ways to reduce spreading HIV through sex, such as the use of condoms. Please ask a member of the study staff if you would like more information on this.

3 ARVs ONLY work if they are taken as you are told by the study doctor.

If you miss doses the drugs will become less effective against the virus. To avoid this it is important to take every single dose of medicine, as instructed by the study doctor.

Try to take your drugs at the same time each day.

---

4 If you lose or run out of either of your ARVs, you should stop taking all of them and come to the clinic immediately. Be sure to bring your medicine bottles and study drug calendar.

5 All ARVs have side effects that more commonly occur during the first few weeks of therapy. Side effects may be mild or severe, and may sometimes require attention.

If you think you are having bad side effects from the ARVs, you should come to the study clinic as soon as possible. The medicines may have to be stopped and you may be started on a different ARV.

6 The study doctor may give you other drugs to prevent infections that occur as a result of HIV infection. These medicines should be taken every day as instructed by the doctor. These medicines include **septrin** to prevent some types of pneumonia and diarrhoea, and/or **Isoniazid** to prevent Tuberculosis.

7 ARVs given to you are meant for you alone. **DO NOT SHARE** your drugs with another person. This can lead to the virus becoming resistant to the ARVs, and make the drugs less effective.

PARTICIPANT INFORMATION ON STUDY DRUGS

One month before you deliver and up to six months after delivery, you will be taking ARVs, either nevirapine and Combivir or nelfinavir and Combivir.

- These drugs interfere with the way the virus tries to reproduce itself in your cells.
- The drugs do not kill the virus but reduce the chances of infecting more cells.
- The purpose of taking these drugs is to reduce the chances of infecting your baby with the HIV virus.
- The drugs work more effectively if taken correctly at the same time everyday.
- Like all other drugs they should be kept out of reach of children.

NEVIRAPINE (NVP)
Nevirapine is given as one tablet once a day for the first 14 days, then one tablet twice a day for the rest of the study. This is done to reduce the chance of rash. Rash is the most common side effect related to use of Nevirapine. If you develop a rash report this to the study staff immediately or report to the clinic. If rash is worsening or if any hives appear, please stop taking Nevirapine immediately and report to the clinic.

Nevirapine may have some effect on the liver. Your blood will be taken to check for any effect on the liver. It is most common in the first weeks of treatment. The other side effects, which may occur, are: nausea, fever and headache. These are usually mild and may wear off soon but in rare instances they can worsen rapidly. If your symptoms are getting worse, you should come to the clinic immediately.

Nevirapine may be taken with or without food. It should not be taken with **rifampicin,** a drug used to treat tuberculosis and **ketoconazole,** a drug used to treat some kinds of infection such as thrush. It also may affect how well **family planning pills** work.

If you have any questions about taking other medications while taking your study drugs, please talk to a study doctor or nurse.

COMBIVIR (3TC/ZDV) This is a combination of two medicines. It contains lamivudine (otherwise known as 3TC) and zidovudine (otherwise known as ZDV or AZT). Combivir is given as one tablet in the morning and one tablet in the evening. It may be taken with or without food.

The most common side effects are nausea, headache, muscle pain and lack of sleep. The most common serious events are anaemia (low blood) and liver problems. During the time you will be on these medicines your blood will be checked regularly to see if the Combivir is causing you to have anaemia or liver problems. The study doctor will treat you if needed.

NELFINAVIR (NLF)

Nelfinavir is given as five tablets in the morning and five tablets in the evening. The most common side effects, which may occur, are nausea, mild to moderate diarrhea, stomach upset. These are usually mild, tolerable, and may wear off soon. If it is too bothersome report to the clinic or follow up staff. Nelfinavir may also have some effects on the liver and may cause changes in blood sugar levels and fat levels. Your blood will be tested regularly to check any of these effects.

Nelfinavir should be taken with a meal or a light snack with a full glass of water; your body absorbs the drug better with food in your stomach. It should not be taken with **rifampicin,** a drug used to treat tuberculosis

It may affect how well **family planning pills** work. Ask the study nurse or other study staff on other Family Planning options.

Some drugs must not be taken with nelfinavir. Always talk to the study doctor or any other doctor before taking other drugs. It is okay to take the other study drugs with nelfinavir.

If you have any questions about taking other medications while taking your study drugs, please talk to a study doctor or nurse.

To get the best results with these medicines:

- You should take them as told by the study doctor
- It is important to fix a timetable of taking medicines as a daily routine
- You should take your drugs at the same time every day
- Have your medicines at your reach all the time to avoid missed doses
- **Do not share** the medicines with anybody
- Report any problem to the clinic or to the follow up staff

If the medicines are not taken well as instructed they will become less effective against the virus and will not work to reduce the amount of virus in the body. The chances of your baby getting the virus will therefore be higher.

For any questions or problems regarding your health and ARVs, please contact a member of the study staff:

8am to 5pm Monday – Friday: Call 057 23200 or 0721 653270 or come to the clinic.

Weekends, holidays, and non-working hours please call 0721 653270, or go to Provincial General Hospital.

If unable to do any of the above, go to your nearest health center.

Please carry your Study ID card.

PARTICIPANT INFORMATION ON USE OF LAMIVUDINE, STAVUDINE AND EFAVIRENZ

(This is only used if participant has a reaction to the routine study drugs)

As you were told before Combivir is a combined pill of 2 drugs, 3TC and AZT. Your doctor may decide that one of these drugs is giving you a problem. In this case you may be continued on one of them as a separate tablet, and the one causing the problem will replaced by a new drug. The most likely drug to be causing a problem is AZT. In this case you will continue with 3TC and one called Stavudine will replace AZT.
In case you will be on anti TB therapy Nevirapine (NVP) will be stopped and you will be given a drug called efavirenz.

LAMIVUDINE (3TC OR EPIVIR)

Lamivudine is a tablet of 150mg. This tablet is taken as one tablet in the morning and one tablet in the evening. It can be taken with or without food. There is less stomach upset if it is taken with food.

The most frequent side effects of lamivudine include headache, nausea, abdominal pains and diarrhea. It can also cause liver problems

During the time you will be on these medicines your blood will be checked regularly to see if the
Lamiviudine is causing you any problems. The study doctor will treat you if necessary.

STAVUDINE (ZERIT)

Stavudine is taken as one tablet in the morning and one tablet in the evening. It can be taken with or without food.

The most common side effects of Stavudine include numbness, tingling or pain in the feet and hands, stomach upset, headache, problems sleeping. Some of these side effects may improve within a few weeks of starting Stavudine. Consult the Study doctor before taking other drugs while you are on stavudine

EFAVIRENZ (STOCRIN)

Efavirenz is a tablet that comes in 600mg. It can be taken with or without food but high fat meals should be avoided. Efavirenz should be taken just before bedtime.

The most common side effects of Efavienz include rash, dizziness, nausea, headache, fatigue, lack of sleep, drowsiness, impaired concentration and abnormal dreaming.
Some of these side effects tend to occur when treatment is just started, but decrease in the first few weeks.

In case of any of the side effects inform the study doctor or any of the study staff.

You should never take efavirenz if you are pregnant or intend to become pregnant. You should use a reliable family planning method.

Certain medications may not be taken with efavirenz consult the study doctor before taking any other medications.

LOPINAVIR/RITONAVIR (KALETRA, ALUVIA)

Kaletra is a capsule containing a combination of 200mg of lopinavir and 50mg of ritonavir. This drug is taken as 2 tablets in the morning and 2 tablets in the evening. It is important that Kaletra is taken with food.

The most common side effects, which may occur, are nausea, mild to moderate diarrhea, stomach upset. These are usually mild, tolerable, and may wear off soon. If it is too bothersome report to the clinic or follow up staff. Kaletra may also have some effects on the liver and may cause changes in blood sugar levels and fat levels. Your blood will be tested regularly to check any of these effects.

Certain medications may not be taken with Kaletra consult the study doctor before taking any other medications.

Avoid exposing this drug to excessive heat; store away from direct sunlight

Flesch-Kincaid Score = 7.3

NELFINAVIR RECALL INFORMATION

INFORMATION TO PARTICIPANTS IN THE KISUMU BREASTFEEDING STUDY: WHY NELFINAVIR (VIRACEPT) WAS RETURNED

Flesch Kincaid 6.9

Since you are, or were, a participant in the Kisumu Breastfeeding Study (KiBS) who took Nelfinavir (also known as Viracept) we want to tell you that the drug was recalled or returned to the company who made it, in June 2007. A recall is a system that removes medicine from the market.

There was a complaint about the smell of some pills. The company, Roche, analyzed the tablets. The tablets had an unexpected substance. The substance is called ethyl mesylate, or EMS. It has been shown that a certain amount of this contaminant can cause cancer in rats. The effects of EMS in humans are not known. There have been no human cases of cancer which were thought to be caused by EMS.

The Nelfinavir that has been used in KiBS was made by a company called Roche. Roche asked for all the Nelfinavir tablets and powder that they had made, to be returned from all countries. The chemical accidentally got into the Nelfinavir while it was being made and a lot of the Nelfinavir made by Roche.

KiBS participants who were on Nelfinavir at the time of the recall were told of the recall. They were asked to return the Nelfinavir they had. The KiBS study got some new Nelfinavir from the United States within one week. The Nelfinavir in the United States is made by a different company, Pfizer and was not returned. All participants who were taking Nelfinavir were either switched to the new Nelfinavir from Pfizer or to a different drug.

Since you took Nelfinavir while in the study, there is a chance that you may have taken the contaminated medicine. There is a chance that your baby was exposed during pregnancy and through breast milk. We don’t know what risk, if any, there is for you or your baby from the EMS. We don’t know which examinations need to be done on you and your baby. We do not know how long examinations need to be performed.

At this time we are working with the company, Roche, to decide what to do to check on your health in the future after KiBS is finished. We want to let you know the status at this time. We would like to contact you in the future about how we will follow-up with you.

If you move in the future please let us know so we may contact you.

Please ask any questions about this issue or any other. We will answer to the best of our knowledge at this time.

Consent for Continued Follow Up Form

Consent for Continued Follow Up after Completing Participation in the Kisumu Breast Milk Study

Flesch Kincaid score: 8.4

For:
Women and their child/children who are current KiBS participants and who are exiting the study
Women and their child/children who have already exited the study and are encountered elsewhere (e.g. at Patient Support Centers)

INTRODUCTION

You and your child/children are now exiting/or have exited the Kisumu Breastfeeding Study (KiBS). You have been involved in KiBS and have taken antiretrovirals to prevent transmission of HIV from mother to child. Not many women or children have gone through a similar type of intervention. It is possible that there will be other studies or other reasons related to your and your child's/childrens' participation in KiBS for which we would want to contact you and your child/children in the future.

At this time we do not know exactly what studies or other reasons these could be or what might be required of you or your child/children, but we are seeking permission from you to contact you and your child/children at some point within the next five years.

We could contact you through whatever mechanism you prefer or give us permission to use. This could include:

P.O. Box
Mobile Phone
Coming to your house where you live now
Contacting your relatives at home
Other mechanism that you suggest

If you agree for us to contact you we will ask you to let us know by which method or methods we can contact you and to provide us with up to date contact information as you did during the study. If this information changes we would ask you to come in to provide us with the new contact information.

AGREEING TO BE CONTACTED IS YOUR CHOICE

1. No one can make you agree to be contacted if you do not want to.
2. To make a choice, you will need information about what it means to provide us with the contact information.
3. Remember that this document is only for you to agree to be contacted. You are NOT

signing an agreement to be in any study.
4. We will not contact your child/children without your permission.

We would like you to ask us any questions you have. We also would like you to tell us when you do not understand something. You may take whatever time you need to make your choice. It is important that you fully understand what we are asking

It is important for you to know that even if you choose to allow us to contact you in the future you may choose to have us not follow up with you at any time

IF YOU AGREE TO BE CONTACTED
We will collect the information that you are willing to provide at this time. We will use this information once a year to contact you to confirm that the information is still valid, however if your contact information changes it would be good for you to inform us at that time.

PRIVACY
The contact information that you give us will not be given to anyone else that is not related to KiBS.

RISKS
The risks to agreeing to us contacting you in the future are minimal. Members of the KiBS staff have already been visiting you at your home, so this would be no different. Those who contact you in the future will not discuss reasons for needing to contact you with anyone else without your consent. They will not disclose your participation in KiBS or your HIV status. We will abide by your wishes on the best way to contact you to minimize any risks to you and your child/children.

COSTS TO YOU
There is no cost to you for allowing us to contact you in the future.

BENEFITS
There will be no direct benefits to agreeing to have us contact you in the future. However, you and your child/children may be contacted in the future to participate in another study which may directly benefit you or your child/children.

YOUR RIGHTS TO REFUSE
You can inform us at any time that you would prefer not to be contacted in the future.

PROBLEMS OR QUESTIONS

If you have questions about being contacted in the future or feel you have been harmed, please contact Dr. Timothy Thomas, Dr. Rose Masaba or Dr. Wairimu Chege at the KEMRI/CDC Research Field station at Kisian, Kisumu. (P.O. Box 1578, Kisumu) at telephone 057-20-22902/59/83.

If you have questions about your rights, you can contact Dr. John Vulule, KEMRI director, at the KEMRI/CDC Research Field Station, (P.O Box 1578, Kisumu) at telephone 057-20-22924/22940 or through 057-20-22902/59/83.

Do you have any questions?

STATEMENT OF CONSENT:

I have read and/or had this form read to me. I understand the purpose of this consent for continued follow up after the end of KiBS.

☐ I agree to allow KiBS staff to contact me in the future

- Through Post office
- Through Mobile phone
- Coming to my house
- Contacting my relatives
- Other method (specify)____

☐ I do not agree to being contacted in the future by KiBS staff

Participant's Name: (Print)		Participant's signature:		Date :	
Witness's Name: (Print)		Witness's Signature:		Date :	

I have explained the purpose of this consent for follow up to the volunteer. To the best my knowledge, he/she understands the purpose, procedures, risks and benefits

Investigator's or designee's Name: (Print) ________________ Investigator's or designee's signature: ________________ Date : _________

Repeat Pregnancy Sub-Study

Factors that influence HIV positive women's decision to become pregnant while participating in prevention to mother-to-child transmission study in Kisumu, Kenya

Funded and Sponsored by:

Division of HIV/AIDS Prevention
National Center for HIV/AIDS, Viral Hepatitis, STD, and TB Prevention
Coordinating Center for Infectious Diseases
U.S. Centers for Disease Control and Prevention
1600 Clifton Rd, Mailstop E-45
Atlanta, GA 30333
U.S.A.

Conducted by:

KEMRI/CDC Program
CDC Research Field Station
P.O. Box 1578
Kisumu
Kenya

Investigators

Dr. Timothy Thomas - MD
Principal Investigator
Kisumu Breastfeeding Study (KiBS)
Chief- HIV-R Branch
Tthomas@ke.cdc.gov

Mary Nyikuri M.A
Behavioral Scientist
HIV-Research Branch
mnyikuri@ke.cdc.gov

Dr. Rose Masaba -MD
Study Coordinator
Kisumu Breastfeeding Study (KiBS)
HIV-Research Branch
Rmasaba@ke.cdc.gov

CDC Atlanta

Eleanor McLellan-Lemal, MA
Behavioral Scientist
Division of HIV/AIDS Prevention
egm4@cdc.gov

Abstract

Roles and Responsibilities:

The study has been collaboratively developed by researchers at CDC/KEMRI with support from CDC/Atlanta. Researchers at CDC/KEMRI will be responsible for data collection and analysis. CDC /Atlanta will provide technical assistance in performing qualitative management, and analysis.

Abstract

Title: Factors that influence HIV positive women's decision to become pregnant while participating in prevention of mother-to-child transmission study in Kisumu, Western Kenya

Justification:

High rates of HIV infection in Nyanza Province in Western Kenya and the relative availability of prevention of mother to child interventions has added complexity in an environment of difficult reproductive decision making for women who are HIV positive and for couples where both or one partner is HIV infected. There is a need to understand factors that influence HIV positive women's decision to conceive a subsequent pregnancy while participating in the Kisumu breastfeeding study- a prevention of mother-to-child transmission study in Kisumu, Western Kenya. This knowledge may help guide interventions and counseling strategies to better inform and support HIV positive women and their families.

Study Objectives:

1. To understand the role of a woman's/partner's demographic characteristics on her decision to conceive a subsequent pregnancy
2. To describe the role of family expectations, parity, disclosure of HIV status, religious belief, and contraceptive usage on subsequent pregnancies/conception

3. To understand the role of information received at KiBS and from the community on pregnancy and being HIV positive on the women's decision making process as regards conception/becoming pregnant

Design: Qualitative research design

Setting: CRC, located at the Nyanza Provincial General Hospital (PGH)

Methodology: Qualitative data will be collected via audio taped individual in-depth interviews. In addition to tape recoding interviews, notes will be taken. A purposive sampling method will be used to identify participants

Sample Size: 30-40 women will be invited to participate in these interviews. Half of these women will be those who have/had a subsequent pregnancy while half will be those who did not have a subsequent pregnancy.

Participants: Participants for this sub study will be drawn from the KiBS main study participants.

Background:

According to the National AIDS Control Council, HIV prevalence in Kenya was 14% in 2000 with rates ranging from less than 2% to 15% for different provinces of the country. Recent estimates by the 2003 Kenya Demographic Health Survey (KDHS) [1] indicate that 7% of Kenyan adults are infected with HIV. The decline, however, is not uniform. In the north eastern part of the country it is less than 1%, while in Nyanza, prevalence remains the highest in the country at 15%. Heterosexual contacts accounts for 90% of HIV infections. Mother-to-child transmission (MTCT) and those infected through contaminated blood account for the remaining 10%. HIV infection varies significantly with age and by sex. HIV prevalence in women and men of reproductive age are roughly 9% and 5% respectively. Young women are especially vulnerable to HIV infection; 4.9% of women aged 15–24 are HIV-infected, compared with 0.9% of men in the same age group [2]

Challenges for HIV prevention arise from societal expectations that men should have sexual intercourse with many women and exercise sexual authority over women. These expectations, among others, sometimes lead men to force sex on unwilling female partners and to reject condom use [3]. Consequently, lack of social and economic power result in the inability of many women to negotiate relationships based on abstinence, faithfulness and use of condoms [3]. Given the high prevalence of HIV among women and their vulnerable status in their relationships with men, a major focus of HIV intervention efforts should then be changing the attitudes and behaviors of men.

Studies that have explored the relationship between an HIV-positive diagnosis and subsequent fertility behavior have found that known HIV status has little association with childbearing. For example, interventions with HIV-positive women in Africa have not been found to result in a significant change in pregnancy rates. Couples appeared to be cognizant of the risk of HIV infection and AIDS—particularly for their children—and sometimes reported changes in contraceptive behavior, yet fertility levels were not affected greatly [4].

Reproductive decision-making is a challenging and complex gender issue involving a wide range of individual, social and biomedical factors. This decision is even more particularly complex for women with HIV and couples where one or both partners are HIV infected because of the added risk of infection to the child. Traditional customs and practices associated with male and female roles and sexuality in many societies compromise the rights and freedoms of reproductive choice among women and sometimes men.

Perinatal transmission and reproductive decisions of HIV-infected women can be categorized in statistical and epidemiological terms. These reports and figures, however, do little to fully explain the complexities of human relationships, life experiences, personal and cultural influences, and situational and environmental variables that impact on the HIV-infected woman regarding reproductive decision-making. It is only with genuine attempts to understand the woman's perspective and the dynamic and unique variables that influence reproductive decision-making, as well as maintaining a non-judgmental and culturally sensitive perspective, can we hope to assist women, and society as a whole, in coming to terms with the complexities of HIV and reproductive decision-making [5].

The Luo are the third largest ethnic group (11%) in Kenya [6]. A study carried out by Angela Ruth Reynar to understand fertility decision-making among Luo couples in Nyanza found out that the husband’s fertility intention had more influence than the wife’s fertility intention on family planning discussions [7]. These social norms and familial obligations influence reproductive health choices of married HIV positive women. Understanding these factors will guide interventions and counseling strategies to better inform and support HIV positive women.

Most HIV-infected women do not know their HIV status before they conceive [8]. Some may only find out when they receive antenatal services, if testing is available. Still, other HIV-infected women who know their HIV status before they conceive, either get pregnant willingly or have no control over this decision, regardless of whether they have disclosed their status to their partner. In a Zimbabwe study, 16 out of 52 HIV-positive women interviewed became pregnant after their diagnosis, and with seven of the 16 pregnancies desired [9].

The Kisumu Breastfeeding Study (KiBS) is assessing the use of maternal HAART from 34 weeks gestation through 6 months postpartum as a Prevention of Mother to Child Transmission (PMTCT) intervention. Women are enrolled and receive highly active antiretroviral therapy (HAART) regardless of CD4 count. The study is an open label Phase II trial of Zidovudine/Lamivudine and either Nevirapine or Nelfinavir (depending on maternal CD4 count) to assess the safety, tolerance and activity of maternal HAART to reduce the risk of transmission among breastfeeding HIV infected women in Kisumu, Kenya. In this study, participants are encouraged to exclusively breastfeed and wean at 6 months. Mother and infant are followed for 2 years post partum.

Between March 2003 and November 2006 KiBS enrolled 522 mothers through antenatal clinics at the Kisumu District and the Nyanza Provincial Hospitals. As part of clinic procedures, participants received counseling on the risk of HIV transmission to the fetus and were encouraged to seriously consider any future pregnancies while still in the stud***y***. Women were also encouraged to disclose their HIV status to their partners and to participate in couple counseling.

In line with the Government of Kenya guidelines and practice, participants are encouraged to use a family planning method starting six weeks post delivery. Injectable progesterone, Depo provera, is the most common form of family planning chosen by women in Kisumu (*Unpublished – Family Health Options*) as well as by KiBS participants. For women, who opted for family planning the study provided counseling, initiated the first dose of Depo provera, others opted for lactation amenorrhea (LAM). Participants who opted for other Family planning methods not available at the study clinic were counseled and referred to the Provincial general hospital within whose grounds the study clinic is located.

However, it was discovered one year into the study that participants were not keeping their Depo provera appointment dates at the referral facilities. 34% of the women returned to the KiBS

clinic anywhere from 5 months post natal to 15 months postnatal for pregnancy screening. To date, 34 participants have become pregnant while participating in KiBS.

KiBS discontinued initiation of Depo provera injections due to lack of continuity by the women at other health facilities. Participants, however, continued to receive, sexual and reproductive health counseling that centers on family planning choices, including how to prevent unwanted pregnancy.

The purpose of this sub-study

HIV infection compromises a woman's body's immune system leaving her more vulnerable to infections. Pregnancy can add stress to this delicate immune system. The purpose of this study is to try and explore factors associated with subsequent pregnancies among women participating in a prevention of mother-to-child transmission study in Kisumu, Western Kenya. Understanding these factors may guide interventions and counseling strategies to better inform and support HIV positive women and their families.

Research Questions

1. What are the socio behavioral (such as cultural norms, ethnic identities, gender norms, religious beliefs and stigma) and socioeconomic factors associated with subsequent pregnancies among KiBS participants

2. Among HIV-positive women, what characteristics differentiate women who become pregnant within 18 months of delivery versus those who did not get pregnant within this same time period?

Sub-study objectives

1. To understand the role of a woman's/partner's demographic characteristics on her conception/ becoming pregnant?
2. To describe the role of family influence, infant/child death, parity, non-disclosure/dislosure of HIV status, strength religious beliefs, and contraceptive usage on subsequent pregnancies
3. To understand the role of information received at KiBS and from the community on pregnancy and being HIV positive on the women's subsequent conception / pregnancy

Methodology

Sub-study Population

The study will include 30-40 women participating in KiBS. Approximately half of the sample will include women who have had subsequent pregnancies and half will include women who have not had subsequent pregnancies. The actual sample size will be determined using a theoretical saturation approach characteristic of constant comparative approaches, such as grounded theory, whereby subsequent interviewing is terminated when it has been analytically determined that no new information is being provided by participants.

Sampling

The purposive sampling method will be utilized in this sub-study. Women who have been identified by study clinicians as either having had or not had subsequent pregnancies while taking part in KiBS will be approached when they come for study clinic visits and invited to take part in an hour-long interview conducted in their language preference.

For the purpose of this study, a subsequent pregnancy is defined as one in which a study participant has completed a 'study' pregnancy and has a second one within the study period. Pregnancy refers to the actual state of being pregnant as assessed by a positive pregnancy test whether or not the fetus is carried to term.

KiBS participants are eligible to take part in this sub-study if they:

1. Are ≥18 years of age
2. Willing to share their experiences and attitudes on pregnancy and HIV testing

Study Exclusion Criteria

Women will be excluded from participation in this study have completed or voluntarily discontinued KiBS participation

Data Collection and Management Methods:

All participants will be interviewed using an open-ended, semi-structured interview guide. This guide will be translated into Kiswahili and Dholuo, and back translated into English by teachers at a local commercial language and translation center. All interviews will be tape recorded, transcribed verbatim, translated into English, and analyzed using a framework approach [10]. Data collected for this sub-study will not be linked to KiBS data collection. Consequently, basic demographic data will be collected from all women who agree to take part in the sub-study to describe general characteristics of the sub-study sample. Demographic questions will be asked about the woman's and/ partner's age, highest level of education, religious beliefs, marital status, income, employment status, partner's HIV status, and her partner's knowledge about her HIV status. Open-ended questions will be used to explore how gender, relationship status, family ties (including her mother –in law), and the number and outcome of previous pregnancies influence reproduction decision-making. In addition, questions will examine sources of emotional support and cultural influences. Women will be encouraged to describe personal pregnancy and HIV testing experiences in their own words.

Recruitment, Enrollment and Interview Procedures

All KiBS participants who have not exited the study are eligible to participate in this sub study. Recruitment will be done both at home and the clinic. During the home and clinic visits, they will receive basic information about the sub-study. Those who indicate interest in participation in the sub-study will be referred to a sub-study interviewer who is also a qualified counselor.The interviewer/counselor will present her with the option of selecting an on-the-spot interview or scheduling a future appointment. The consenting and interviewing will take place in a quiet location either in/near the participant's home or clinic, depending on the participant's preference. Before data collection is initiated, informed consent will be obtained from all participants. A participant opting to complete an interview at a future date will be reminded of their appointment in advance through the follow up team and when they come to the clinic for scheduled and unscheduled visits. If a woman fails to show up for a scheduled appointment or does not initiate rescheduling, no attempts will be made to contact her to include her in the sub-study..

Quality Control

Interview transcripts will be read 3-5 times by the PI and the interviewer in order to ensure (1) that English translations are consistent with meanings presented in Kiswahili and Dholuo. Systematic data analysis will be undertaken to ensure that the thematic framework is authentic to the experiences described by participants and that all data has been included.

Data Analysis

The interviewer will participate in data analysis. A qualitative data analysis framework approach [10], which involves 3-5 passes of the data, is comprised of five steps:

1. Familiarization – as the transcripts are reviewed, a list major ideas and recurrent themes is generated
2. Identification of a thematic framework – major themes and issues by which data can be examined are created

3. Indexing – the thematic framework generated is applied to all of the data using a numerical annotation coding scheme
4. Charting – charts (display tables) containing distilled summaries of key participants' views and experiences are prepared
5. Mapping and interpretation – charts are examine to identify associations between themes and generate explanatory models for key finding

Participant Privacy and Protection of Sub-study Records

The names of individual study participants will not be required at any point in the interview process. All individuals will be assigned a number in place of their name. Consequently, no names will be used in any written or spoken reporting of the results. All interview notes and tapes will be secured safely in locked cabinets, and accessed and used only by authorized study personnel without revealing the identities of individual participants. Audio files, interviewer notes and prepared transcripts will be destroyed once the analysis has been completed and results published.

Incentives

There is no cost to KiBS participants to take part in this sub-study. Participants will be reimbursed transport according to standard KiBS guidelines already described in the main study protocol. For their time, sub-study participants, like all KEMRI/CDC participants will receive a bar of soap.

Informed Consent

Participation in this sub-study does not involve collection of identifying information and presents no more than minimal risk to human subjects. All participants will be required to undergo a standard informed consent procedure consistent with international recommendations - Helsinki Declaration 2000, CIOMS 2002 [11]. The informed consent, which will be translated into the two predominant languages in Kisumu (Kiswahili and Dholuo), will describe the purpose of the study, the procedures to be followed, and the possible benefits and risks of participation. The informed consent document will be read to all prospective participants by the interviewer. Participants will have the opportunity to ask questions and receive clarification on anything they do not understand regarding participation in this sub-study. An independent witness will observe the consenting procedures and provide a witnessed signature. A copy of the consent will be provided to the participant.

Each participant will be asked to provide signed documentation. The participant can provide her signature, thumbprint, or an X mark on the signature line.

Time Frame/Duration of study

The study will begin in within one month of IRB approval and will continue until for 6 months.

Key Milestones	Duration
Translation of consent document and interview guides into Kiswahili and Dholuo	5 days
Purposive sampling	6 days
Recruitment of women for interviews	6 days
Conducting the interviews	15 days

Transcription and translation of interview data	28 days
Data analysis	21 days
Writing of abstract	5 days
Editing and proofreading of abstract and submission for clearance	21-30 days
Submission of abstract to conference/ journal	1 day
Writing of manuscript	28- 50 days

REFERENCES

1. Central Bureau of statistics (CBS) [Kenya], ministry of Health (MOH) [Kenya], and ORC Maacro. 2004. *Kenya Demographic and Health Survey 2003*. Calcerton, Maryland: CBS, MOH, and ORC Macro.

2. AIDS in Kenya: Trends, Interventions and Impact. Seventh Edition, 2005

3. *IPAR Policy Brief Volume 10, Issue 12, 2004)* Gender Aspects in HIV/AIDS Infection and Prevention in Kenya

4. Williams HA, Watkins CD, Rsiby Ja. Reproductive decision making and determinants of contraceptive use in HIV infected women. Clin Obstet Gynecol. 1996 Jun; 39 (2): 333-43

5. Feldman R, Maposhere C. Voices and choices: a participatory research and advocacy study of reproductive health and rights of HIV positive women in Zimbabwe. *The XIII International AIDS Conference*, Durban, South Africa, July 9-14, 2000.

6. Ogot, Bethwell A., *History of the Southern Luo: Volume I, Migration and Settlement, 1500-1900*, (Series: *Peoples of East Africa*), East African Publishing House, Nairobi, 1967

7. Angela R.R. 2000 Fertility Decision Making amongst the Luo of Kenya; University of Pennyslavania

8. Otieno F, Munene F, Marum L, Cross A, Muttunga J, Chebet K AIDS awareness and knowledge of HIV serostatus in the Kenya Demographic and Health Survey 2003 (KDHS+).*Int Conf AIDS.* 2004 Jul 11-16; 15: abstract no. MoPeC3499.

9. Naomi Rutenberg, Ann E. Biddlecom and Frederick A. D. Kaona Reproductive Decision-Making in the Context of HIV And AIDS: A qualitative Study in Ndola, Zambia *International Family Planning Perspectives Volume 26, Number 3, September 2000*

10. Pope C, Mays N, editors. Qualitative Research in Health Care. 2nd ed. London: BMJ Books; 2000.

11 .Council for International Organizations of Medical Sciences (CIOMS) in collaboration with the World Health Organization (WHO) (2002). International Ethical Guidelines for Biomedical Research Involving Human Subjects. Geneva: Switzerland.

APPENDICES to Repeat Pregnancy Sub-study

Appendix A.   Consent Form

English Version of the subsequent pregnancy consent to take part in research
Flesch-Kincaid Grade level 7.1

Informed Consent:
We would like to invite you to participate in a study called the 'Subsequent Pregnancy Study'

Purpose: This is a study to to look at reasons why women in the Kisumu Breastfeeding Study (KiBS) have become pregnant again during the study.

Who is doing the study: The study is being done by KEMRI/CDC staff.

What will happen in the study?
About 40 women will be asked to take part in the study. We want to talk to both women who have had a subsequent pregnancy and women who have not, to see if there are any differences. Half of the women who participate will have become pregnant during the study period, while the other half will not have become pregnant during the study period. Participants will be asked to share their stories about factors that influenced them to become pregnant/not to become pregnant while still in the KiBS.

Taking part in this study is up to you. If you choose to take part, you will be given an appointment for a discussion/interview either in the clinic or a quiet place of your choice. The interview will be done by one of the KiBS staff who is a counselor. If you choose to take part in the study, you can leave the interview and you can choose not to answer certain questions.
Are there any benefits from participating in this study
You may not benefit directly from being in this study. However, the information we collect during this study may help us to better understand family planning issues among women who have HIV. Results may help design future family planning counseling for women
Are there any risks of participating in the study
The risks of being in this study are low. Some of the questions we ask you during the interview may make you feel uncomfortable. We can skip any questions that you do not want to answer. All the information obtained will be kept private. We will not disclose your status or any information to others not involved in this study.
Is there compensation for time and effort
There are no costs to you to take part in this study. It will take about an hour of your time to complete the interview. After you finish the interview, we will give you a bar of soap in appreciation for your time. We will also re-imburse your transport in-line with KiBS rates if the interview is done at the clinic.
How long will the study last?
The study will take about six months before a summary of the findings are shared with you. However, the interviews with the women will be conducted over about a month. It is anticipated that each interview will last about an hour.

Who can take part?
Any KiBS participant, but we must have some who have a subsequent pregnancy and some who have not had a subsequent pregnancy during the study.
Voluntary Participation
Being in this study is up to you. If you agree to take part, you do not have to answer any question that you do not want to. You are free to stop taking part in this study at any time. If you decide to stop the interview at any time, this will not affect your participation in KiBS.
Who will see the information you provide?
We will keep all your information private. During the interview we will not use your name. We will assign your interview a study identification number. All of your records will be kept in a locked filing cabinet at CDC/KEMRI. Only people working directly on the study will be able to look at these records. Any reports that come out of this study will not have your name or anything that could identify you.

Who can you contact about the study or your rights as a volunteer in this research study?
If you have questions about this research or feel you have been harmed by the research, please contact Ms.Mary Nyikuri at the KEMRI/CDC Research Field station at CRC, Kisumu. (P.O. Box 1578, Kisumu) at telephone 057-20-22902/59/83.

If you have questions about your rights, you can contact Mr. Bryson Ndenga, KEMRI Kisumu SSC Secretary at the Center for Global Health Research –Kisian Field Station, (P.O Box 1578, Kisumu) at telephone 057-20-22924/22940. Email: Bdenga@kisian.mimcom.net

Use of tape recorder
The interviewer will ask you if s/he can record the interview. You can either agree or disagree. If you agree, the interview will be tape-recorded. If you don't agree, then the interviewer will not record the interview.

If there is any portion of this consent agreement that you do not understand, please ask the study team.

Statement of Consent:
I understand the purpose of the study and my questions about it have been answered. I agree to take part by my own choice.

______________________	______________________	__________
Volunteer's Name<br>*Type or print*	Volunteer's Signature	Date
______________________	______________________	__________
Witness's Name<br>*Type or print*	Witness's Signature	Date

I have explained the purpose of this study to the volunteer. To the best of my knowledge, she understands the purpose, procedures, risks and benefits of this study.

____________________________	______________________________	_____________
Investigator/Designee Name<br>Type or print	Investigator/Designee<br>Signature	Date

Repeat Pregnancy Sub-study Appendices

Appendix A. Consent Form

Luo Version of the subsequent pregnancy consent to take part in research

Flesch-Kincaid Grade level 7.1

Informed Consent: ***(this luo version has been back translated and found to be a true reflection of the original English version)***

Wahero mar rwaki enonro mang'iyo gigoma miyo mine man kod kute mag ayaki bedo mayach ekinde ma pod gin e nonro mar dhodho nyithindo (KiBS)

Nonro ni itimo gi Migawo mar Jo KEMRI/CDC. Mine manyalo romo 40 ibiro kwayo mondo odonji e nonroni. Nus mar minegi biro bedoni yach kapodi gin e nonro to nus ma odong nobedi ni ok oyach.

Donjo en yiero mari

Donjo e nonroni en yiero mari. Ok ochuni mondo idonji e nonro ni ka ok idwar mondo wakonyi chopo e paro mar yiero, wabiro nyisi gik ma biro timre e nonro. Yie ibed thuolo mar penjo penjo maingo. Dawaher mondo inyiswa kama ok oleroreni maber. Yie ikaw thuolo mari eka itim yiero.

Owinjore mondo ing'e chutho gik ma nonro biro dwaro ni itim. Bende ber mondo ing'e ni kata ka iyiero donjo e nonro to inyalo wuok enonro saa saya, bende ok ochuni ni nyaka iduok penjo ma ok idwar duoko.

Kidonjo enonro to wabiro penji gik ma ne dimi ibed mayach kendo ka pod in e nonro kata ma ne omoni mako ich kendo.

Rach manyalo bedoe

Rach manyalo bedoe nonro ni tin. Penjo moko nyalo bedo matek ni duoko wanyalo weyo maok wapenji penjo maok idwar duoko.

Ber manyalo betie

Dipo ka ok iyudo ber kuom bedo e nonro kata kamano weche ma inyisowa biro konyowa ng'eyo matut gima omiyo mine moko bedo mayach kendo kapod gin e nonro. Duoko nyalo konyo loso chenro mabiro mar chano nyuol, wuoyo mar hocho to kod gengo ne nyithindo yudo kute mag ayaki koa kuom minegi.

Keno Apanda

Wabiro kano weche ma inyisowa duto eyo ma Opondo. Kendo ok wabitiyo gi nyingi ka wapenji penjo wabiro miyi namba manyiso ni in e nonro. Weche ma inyisowa duto ibiro kan e kar keno ma olor makmana jo ma tiyo e nonro emabiro ng'iyo weche ni ma okan.

Duoko ni duto moa e nonroni ok no ketie nyingi kata gima nyiso ni en mari.

Chudo

Ongee chudo ma ibiro duar kuomi e nonroni. Biro kawo thuolo madirom saa achiel mondo watiek penji penjogo, bang'e wabiro miyi sabun miti achiel mar erokamano kuom thuolo ni. Bende wabiro miyi pesa wuoth kaluwore kaka KiBS chiwo.

Chandruok kata penjo

Ka in gi penjo modok kor ka nonro ni to inyalo tudri gi:

Ms. Mary Nyikuri manie CDC/KEMRI study clinic at New Nyanza Provincial General Hospital on Kakamega Road, Kisumu (P O Box 1578 Kisumu) e namba simu: 05720530117/8

Kata

Mr. Bryson Ndenga, KEMRI Kisumu SSC jandiko mar center no Global Health Research –Kisian Field Station, (P.O Box 1578, Kisumu) .*Namba mar simu: 057-20-22924/22940. Email: Bdenga@kisian.mimcom.net*

Yiero donjo e nonro

Bedo e nonro ni en yiero mari. Kiyiero bedo e nonroni ok ochuni duoko penjo ma ok idwar duoko inyalo wuok e nonro saa saya mihero.

Tiyo gi nyakalondo ma mako wach

Ja tim nonro biro penji kiyie mondo omak weche minyise e nyakalondo. Inyalo yie kata dagi. Kiyie to ibiro mak weche go e nyakalondo ka idagi to ok no mak weche go.

Ayie mar donjo e nonro

Awinjo gima omiyo itimo nonroni kendo penjo na bende ose duoki. Ayie kendo en yiero na mondo adonji e nonroni.

________________________ ________________________ ______________________

Nying jachiwre Luet jachiwre Tarik

________________________ ________________________ ______________________

Nying janeno Luet janeno Tarik

I have explained the purpose of this study to the volunteer. To the best of my knowledge, she understands the purpose, procedures, risks and benefits of this study.

Jatim nonro<br>(kata ng'ama nie loye)	Luet jatim nonro<br>(kata ng'ama nie loye)	Tarik

APPENDIX B

IN-DEPTH INTERVIEW GUIDE (*To be translated into both Kiswahili and dholuo and back-translated for quality check)*

Factors that influence HIV positive women's decisions to become pregnant while participating the Kisumu Breastfeeding Study (KiBS) in Kisumu, Kenya

Sub-study ID

Time: From _________To ____________

Date: ☐☐/☐☐ /☐☐

Venue: ______________________________

INTRODUCTION

Introduction of Interviewer:
Hello, my name is ________. I am (describe affiliation with the study). We are conducting individual interviews to find out what factors influence women's decisions to become pregnant or not to become pregnancy while taking part in KiBS.

Everything we discuss will be kept private. What we learn from this study will be used to improve future Mother to child Transmission research interventions. This summary of the findings will be shared with staff at the CDC Clinical Research Center, KEMRI and other organizations working on future trials. No personal information will be shared and your name will not be included in any of these reports.

Because there will be a lot of information that I will not be able to remember or write down, I would like to tape record this interview. The audiotape will be typed word-for-word. Your name or any other personal information that you share with me that may identify you will not be included in the typed record of the interview. Once the typed record has been reviewed for accuracy, the audiotape will be destroyed.

The interview will take about one hour to complete. There is no right or wrong answer to any of the questions I ask you. I may ask follow-up questions so that I can better understand your responses.

We really want you to speak from you own experiences. We also want you to be comfortable during the interview. If you don't understand a question, please feel free to ask me to explain. Also, if you would prefer to skip a question, please let me know. I will then move to the next question. Because your experiences and points of view are important, we ask that you wait until the end of the interview to ask for information.

Do I have your permission to tape record this interview?

(TURN ON TAPE RECORDER)

Demographic Characteristics

1. How old are you?
2. What is your ethnic group?
3. What is your marital status?
    a. If married, how long have you been married?
    b. Does your husband/partner have another wife?
        i. If yes, how many children does your co-wife have?
    c. If not married, does the father of your child have a wife? (wives)
        i. How many children does his wife (wives) have?
4. Do you live with your husband/partner?
5. How old is your partner/husband?
6. What is the level of his education?
7. What is his occupation?
8. What is his ethnic group?
9. How many times have you been pregnant?
    a. How many sons do you have?
    b. How many daughters do you have?
10. Have you ever had child/children who died?
    a. How many were boys?
    b. How many were girls?
11. What is your desired number of children?

Socio-economic status

1. What is the highest level of education that you have completed?
2. Do you do anything to earn a living? If yes, what? How much money do you earn/make per month?
3. Does your spouse/father of your child/ren support you financially?

Religious Belief
Do you have any religion? If yes, which one?
Does your religion have any teachings about pregnancy and child bearing? Which are these teachings?
Does your religion hold any views regarding conception out of marriage? What are these views?
Does your religion have particular views on a woman that does not have children? Please share these views?

Perceived efficacy of disease:
Does HIV have any effect on the health of a pregnant woman? What are these effects? How can these effects be dealt with? Re there steps that can be taken to ensure one stays healthy? What are these steps? How would describe your health now? Are there treatments that one can get for HIV? What are these treatments? What treatment (s) are you getting for HIV/AIDS? Were you on any other treatment at the time of conception? Which one? Did/do you have any concerns during pregnancy? Please share with me. Do you know the HIV status of your baby (referring to the main KiBS baby) What about your other children (if she had others before joining KiBS or is she has already delivered the subsequent baby). Would you like to tell me? What is the status?

Family expectations
Are there expectations a family places upon a newly married couple? What are these expectations?
Does your family/community place any value on children? What value? How is this value demonstrated? (What does having children mean in their culture)
Are women who do not have children treated differently? Please explain.

Disclosure
How would you describe your relationship with your husband/partner?
Do you discuss family matters, including the welfare of your children? If yes, what do you discuss? Are there some matters that you would rather not discuss with him? If yes, which matters and why?

Contraceptive use
Are you aware of any methods of contraceptives? Please name them. Have you used any? If yes, which ones? Do you have any concerns about contraceptives? If yes, please explain.

Value of information provided regarding prevention of mother-to-child transmission of HIV at KiBS.

Did you make plan to become pregnant? If so, what influenced you?
Did you use any contraceptives? What prevailing factors contributed to your use/none use of contraceptives? What else would you like to talk about in relation to contraceptives?

This ends our interview. Thank you for your thoughtful responses. Do you have any questions for me?

Appendix C

BUDGET

<u>Participant Costs, Travel and Reimbursement</u>

Travel and time compensation for research participants (1 interview x 300 shillings for 40 people	12,000/-
Supplies	10,000
Communication (air time and transport by the follow up staff to contact the participants)	5,000
Translation costs	10,000
Training interviewer	00
**Subtotal**	**KSh 37,000**

<u>Equipment</u>

Tape recorders (2)	10,000
Recording supplies	10,000
**Subtotal**	**20,000**

TOTAL in Kenyan Shillings (based on rate of 70 KSh/1 US$_________ KSh 57,000

Budget notes:

Most of the sub-study costs will be absorbed by the KiBS study.
